# Transcriptome-informed identification and characterization of *Planococcus citri cis*- and *trans*-isoprenyl diphosphate synthase genes

**DOI:** 10.1016/j.isci.2024.109441

**Published:** 2024-03-06

**Authors:** Mojca Juteršek, Iryna M. Gerasymenko, Marko Petek, Elisabeth Haumann, Sandra Vacas, Kalyani Kallam, Silvia Gianoglio, Vicente Navarro-Llopis, Michael Heethoff, Ismael Navarro Fuertes, Nicola Patron, Diego Orzáez, Kristina Gruden, Heribert Warzecha, Špela Baebler

**Affiliations:** 1 National Institute of Biology, Department of Biotechnology and Systems Biology, Večna pot 111, 1000 Ljubljana, Slovenia; 2 Jožef Stefan International Postgraduate School, Jamova 39, 1000 Ljubljana, Slovenia; 3 Plant Biotechnology and Metabolic Engineering, Department of Biology, Technical University of Darmstadt, Schnittspahnstrasse 4, 64287 Darmstadt, Germany; 4 Centre for Synthetic Biology, Technical University of Darmstadt, Schnittspahnstrasse 4, 64287 Darmstadt, Germany; 5 Instituto Agroforestal del Mediterráneo-CEQA, Universitat Politècnica de València, Camino de Vera s/n, Valencia, Spain; 6 Engineering Biology, Earlham Institute, Norwich Research Park, Norwich, Norfolk NR4 7UZ, UK; 7 Institute for Plant Molecular and Cell Biology (IBMCP), Consejo Superior de Investigaciones Científicas (CSIC) - Universitat Politècnica de València (UPV), Valencia, Spain; 8 Animal Evolutionary Ecology, Department of Biology, Technical University of Darmstadt, Schnittspahnstrasse 4, 64287 Darmstadt, Germany; 9 Department of Organic Chemistry, University of Valencia, Burjassot, València, Spain

**Keywords:** Biochemistry, Entomology, Omics, Phylogenetics

## Abstract

Insect physiology and reproduction depend on several terpenoid compounds, whose biosynthesis is mainly unknown. One enigmatic group of insect monoterpenoids are mealybug sex pheromones, presumably resulting from the irregular coupling activity of unidentified isoprenyl diphosphate synthases (IDSs). Here, we performed a comprehensive search for IDS coding sequences of the pest mealybug *Planococcus citri*. We queried the available genomic and newly generated short- and long-read *P. citri* transcriptomic data and identified 18 putative IDS genes, whose phylogenetic analysis indicates several gene family expansion events. *In vitro* testing confirmed regular short-chain coupling activity with five gene products. With the candidate with highest IDS activity, we also detected low amounts of irregular coupling products, and determined amino acid residues important for chain-length preference and irregular coupling activity. This work therefore provides an important foundation for deciphering terpenoid biosynthesis in mealybugs, including the sex pheromone biosynthesis in *P. citri*.

## Highlights

• Long- and short-read transcriptome sequence resource of *Planococcus citri* was built
• Potential *P. citri* IDS coding sequences were identified
• Five *P. citri* IDSs produced regular short polyprenyl chains
• *Trans*IDS5 produced low amounts of irregular polyprenyl chains

## Graphical abstract

**Figure:**
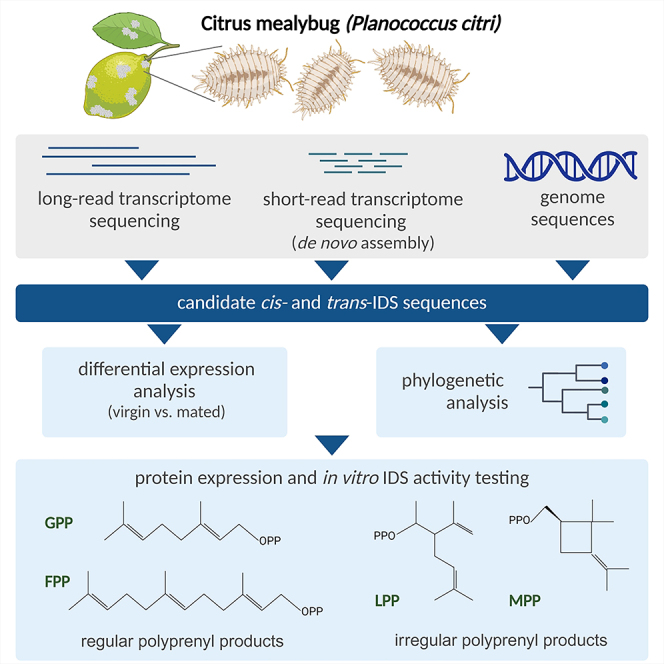

## Introduction

Terpenoids are a large class of metabolites produced by organisms in every branch of life. In insects, terpenoids have important roles in development (e.g., juvenile and moulting hormones) and as semiochemicals in intra- as well as interspecific interactions (e.g., sex, aggregating, alarm, dispersal, maturation, anti-aphrodisiac or trail pheromones, and defense compounds).^1^^,^^2^ Of special interest are the terpenoid sex pheromones produced by some mealybug and scale insect species (order Hemiptera, Superfamily Coccoidea). Sex pheromones are chemical signals that mediate communication between males and females of a species and are important for mating. In Coccoidea, they are produced by females to facilitate male navigation during mating and are thus emitted by virgin females, with cessation of production after mating.^3^ The majority of mealybug (family Pseudococcidae) and armoured scale insect (family Diaspididae) sex pheromones identified to date are irregular terpenoid compounds, mostly esterified mono- or sesquiterpenoid alcohols and carboxylic acids.^3^^,^^4^ Chemically synthesized mealybug and scale insect pheromones have been successfully used for pest control, providing a sustainable measure in climate change-challenged agriculture.^3^^,^^5^^,^^6^^,^^7^^,^^8^ Although an extensive body of research has been dedicated to the identification and study of mealybug and scale insect sex pheromones, the biosynthesis of their irregular backbone remains elusive.

The first step of terpenoid biosynthesis is catalyzed by isoprenyl diphosphate synthases (IDSs or prenyltransferases), which couple the C5 terpene building blocks, isopentenyl diphosphate (IPP) and dimethylallyl diphosphate (DMAPP) into C10 (geranyl diphosphate, GPP), C15 (farnesyl diphosphate, FPP), C20 (geranylgeranyl diphosphate, GGPP), and longer prenyl diphosphate precursor chains by sequential condensation steps.^9^ The prenyl diphosphate chains are then transformed into terpenoid compounds through the activity of terpene synthases (TPSs).

IDSs can be classified as either *cis*- or *trans*-, depending on the stereochemistry of the double bond in the reaction product. The two classes are evolutionarily and structurally distinct. *Trans*-IDSs adopt an α-fold and contain two conserved aspartate-rich motifs known as FARM (first aspartate-rich motif) and SARM (second aspartate-rich motif), which mainly occur as “DDxx(x)D”. *Cis*-IDSs, however, lack distinct conserved motifs and adopt the ζ-fold.^9^ Aspartate-rich motifs in *trans*-IDSs coordinate the Mg^2+^ ions important for the generation of the carbocation in the allylic substrate (DMAPP, GPP, FPP, or other), which can be attacked by IPP to form the new carbon-carbon (C–C) bond in the alkylation reaction. The coupling step catalyzed by IDSs can be also classified based on the orientation of the alkylation reaction. Most IDS enzymes catalyze the formation of regular 1′-4 (head-to-tail) C–C bond between the allylic substrate and IPP, while irregular head-to-middle reactions have been described for some *cis*- and *trans*-IDS enzymes, coupling two DMAPP units or DMAPP with an allylic substrate, forming branched or cyclic polyprenyl chains.^10^ To date, only a few enzymes with irregular coupling activity have been identified from plants (e.g.,^11^^,^^12^^,^^13^^,^^14^), bacteria (e.g.,^15^^,^^16^), and archaea (e.g.,^17^^,^^18^).

*Planococcus citri* (Risso, 1813), the citrus mealybug, is a small sucking insect. It is a widespread pest of numerous crops and ornamental plants, causing serious damage and economic losses.^19^ Its sex pheromone, planococcyl acetate, has a cyclobutane backbone, presumably resulting from irregular IDS coupling. It has been successfully chemically synthesized and deployed in integrated pest management via trap-based management strategies, which require only milligram quantities.^20^^,^^21^^,^^22^^,^^23^ Alternative management strategies, such as mating disruption, demand larger quantities and, therefore, require low-cost, scalable production methods.^3^ Biotechnological production of sex pheromones has been demonstrated recently for lepidopteran pheromones^24^^,^^25^ and biotechnological synthesis has been proposed as a sustainable route for large-scale, low-cost pheromone production. However, these methods require knowledge of the biosynthetic pathways.

To that end, we performed a comprehensive search of the *P. citri* genome, supported by newly generated short- and long-read sequence datasets of *P. citri* females, to identify candidate *cis*- and *trans*-IDSs. Eleven of the identified IDS sequences were tested for regular and irregular coupling activity, with a focus on the production of short-chain backbones. We confirmed regular coupling activity for five *trans*-IDS candidate enzymes, one of which also produced small amounts of irregular prenyl diphosphates, lavandulyl and maconellyl diphosphates. Our work contributes to the understanding of mealybug terpenoid biosynthesis providing a foundation for its biotechnological exploitation.

## Results

### *Planococcus citri* short- and long-read transcriptome resource

To complement the available *P. citri* genomic sequence data (*Pcitri*.v1 genome assembly, deposited at MealyBugBase^26^) we generated short and long transcriptome reads by performing RNA-seq and Iso-seq. With the transcriptome data, we aimed to obtain reliable coding sequence information at high depths, enabling a comprehensive search for IDS candidates expressed in *P. citri* or symbiotic organisms. Additionally, virgin and mated female-specific transcriptomic data were generated to perform differential expression analysis and detect possible pheromone synthesis-related changes in gene expression and therefore support the characterization of putative IDSs involved in sex pheromone biosynthesis. This was based on the hypothesis, that sex pheromone biosynthesis genes are expressed in virgin females only, as they are also the ones producing the pheromone compounds.^27^

Short Illumina reads from virgin and mated *P. citri* females generated in this study were *de novo* assembled on its own (Figure S1A), and together with other available *P. citri* short-read sequence data, including reads from *P. citri* males (Figure S1B). Both assemblies were combined, resulting in 389,962 unique sequences (Figure S1C). After the merge with the long-read transcriptome (Figure S1D), the full consolidated set contained 440,881 unique transcript sequences (Figure S1E), with an average length of 1,356 nucleotides and N50 of 2,908 nucleotides (Table S1). On average, around 80% of short reads from each sample mapped back to *de novo* transcriptome assembly (Table S2). To retain as much variability as possible, we did not do any further assembly thinning and the presented dataset is, therefore, redundant. Approximately 53% of the transcripts mapped to the *Pcitri*.v1 genome, with a third of them mapping to the annotated *Pcitri*.v1 gene models. Completeness assessment with BUSCO^28^ found 98.7% complete BUSCOs for the consolidated transcriptome, the majority of which were duplicated (Figure S2). Transcript IDs with their InterPro annotations, information on mapping to *Pcitri*.v1 genome, and virgin versus mated female differential expression values are available in Table S3A, as well as on FAIRDOMHub along with a fasta file with all sequences from the consolidated transcriptome (see Data and code availability).

Due to the elusive nature of terpenoid biosynthesis in insects, it has been hypothesized that the origin of some terpenoids (or their precursors), might not be the insects themselves, but rather endosymbiotic bacteria or even plant food.^29^ For a comprehensive analysis of the biosynthetic capacity for terpenoids found in *P. citri*, our transcriptomic resource, therefore, contains transcripts of all taxonomic origins, as we elected not to exclude non-insect reads. Taxonomic analyses revealed that out of 207,753 protein sequences determined from the consolidated transcriptome, 26% were unclassified, with the majority of classified sequences assigned to insects (Figure S3). 2,641 sequences were assigned to bacteria, 1,010 of which correlated best with sequences from *Candidatus* Moranella endobia, a secondary endosymbiont of *P. citri*. The acquired data also includes 2,364 viral, 1,629 plant, and 4,299 fungal sequences. Of the plant sequences, most were assigned to potato (*Solanum tuberosum*), probably originating from the *P. citri* food source. Of the fungal sequences, most were assigned to *Wallemia mellicola*, a cosmopolitan fungus with air-disseminated spores.^30^

### Identification of putative IDS coding sequences

To find sequences with homology to *cis*- and *trans*-IDS enzymes, we searched *Pcitri*.v1 genome assembly as well as the *P. citri* consolidated transcriptome (Figure S1E) for sequences with assigned InterPro family annotations IPR001441 and IPR036424 (for *cis*-IDS sequences) or IPR000092 (for *trans*-IDS sequences). On the *Pcitri*.v1 genome assembly we also performed MAST motif search. All extracted sequences (Tables S4A and S4B) were collapsed into clusters based on their sequence similarity to manually inspect possible assembly or sequencing errors and determine the most probable consensus sequence. This resulted in a final number of 30 putative IDS sequences (Table S4C).

**Figure 1:**
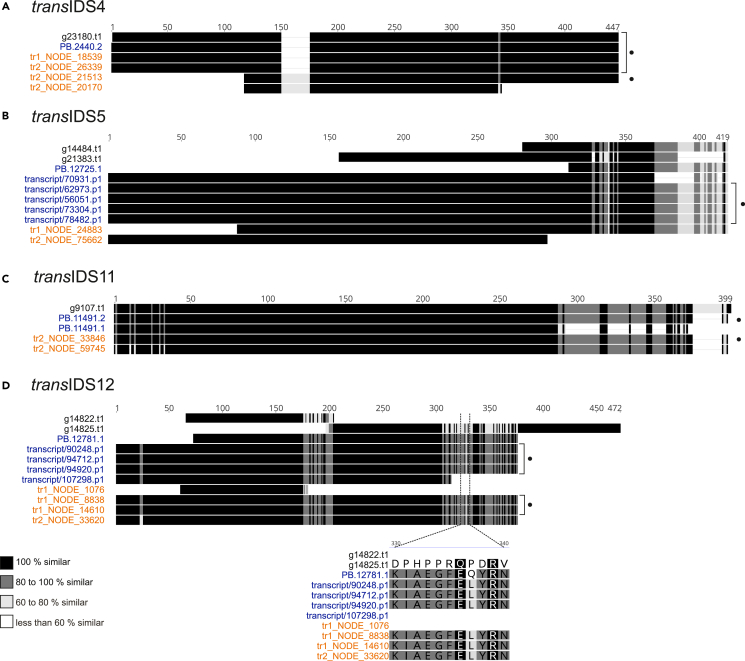
Identification and confirmation of IDS coding sequences predicted by transcriptome data Multiple sequence alignments for clusters of overlapping transcripts and gene models for candidate sequences *trans*IDS4 (A), *trans*IDS5 (B), *trans*IDS11 (C), and *trans*IDS12 (D). Each cluster includes translated coding sequences obtained from *Pcitri*.v1 gene models (in black text), long-read transcripts (in blue text), and transcripts *de novo* assembled from short reads (in orange text). All sequences are available in Table S4C. Sequences confirmed with amplification from cDNA and Sanger sequencing are marked with black dots. Color-coded sequence similarity is given in the legend in the bottom left. The alignment around the L328 versus Q328 variation is shown below the full-length alignments for *trans*IDS12 in (D). Multiple sequence alignment was done in MEGAX using the MUSCLE algorithm and visualized with Geneious software.

Newly acquired long- and short-read data provided alternative transcript sequences to matching *Pcitri*.v1 gene models of the same cluster for seven IDS sequences as well as information on possible sequence variability (Figures 1 and S4). For five candidates (*cis*IDS1, *trans*IDS4, *trans*IDS5, *trans*IDS11, and *trans*IDS12) we were able to confirm the sequences predicted by the transcriptome data with amplification (Figure 1). Two different amplicons were obtained in the case of *trans*IDS4 (Figure 1A), one identical to the gene model and one with N-terminal truncation and an insertion of 25 amino acids. For *trans*IDS5 (Figure 1B), the gene model was missing 283 amino acids at the N-terminus. For *trans*IDS11 (Figure 1C), we amplified a sequence with a frameshift close to the C-terminus resulting in a 21 amino acids long C-terminus truncation. In the case of *trans*IDS12 (Figure 1D), the structure of the amplified sequence differed significantly from the annotated gene models. Additionally, two variations of the same full-length *trans*IDS12 sequence were amplified, differing in a single amino acid position: L328 versus Q328 (Figure 1D). We also detected other single nucleotide polymorphisms (SNPs). For example, the translated amplicon of *cis*IDS1 has Leu (L327) instead of His at position 327 (H327), the latter predicted in the gene model, while the transcripts predicted from the *de novo* assembly or fully sequenced all have L327. Examining the mapping of short reads to the genome assembly, we observed that approximately 80–90% of the short reads correspond to L327 and the rest to H327.

Eight of the identified IDS sequences most likely originated from other species: *trans*IDS14 has only one mismatch to an FPPS (farnesyl diphosphate synthase) from potato, while *trans*IDS15, *cis*IDS7, and *cis*IDS10 have 100% identity to different IDS sequences from the fungus *Wallemia mellicola* (Table S4C). *Trans*IDS6, *trans*IDS7, *cis*IDS4, and *cis*IDS6 have homology to insect IDS sequences. However, as determined by phylogenetic analysis (Figures S5–S8), these grouped with homologous sequences from Chalcidoidea wasps (Hymenoptera). Additionally, one sequence can be assigned to a *P. citri* symbiont: *cis*IDS8 is identical to a protein annotated as a UPPS (undecaprenyl diphosphate synthase) from *Candidatus* Moranella endobia.

### *In silico* analysis reveals diverse putative functions of candidate *P. citri* IDSs

**Figure 2:**
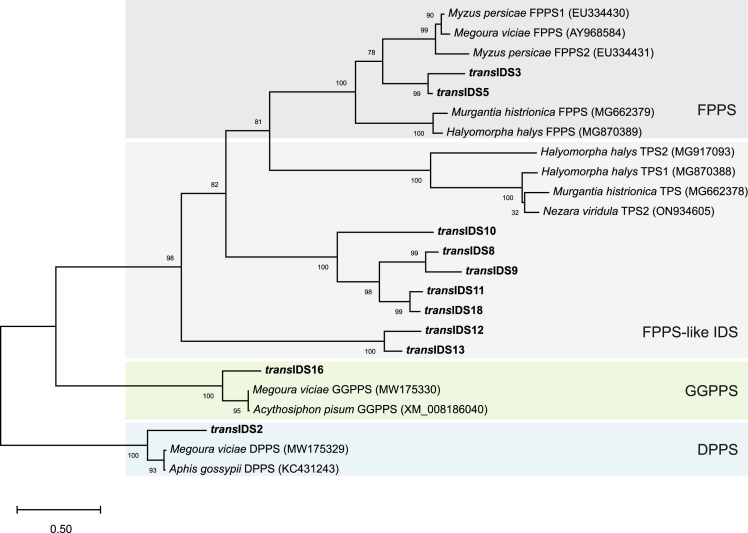
Phylogenetic tree of *P. citri trans*-IDS candidates and characterized *trans*-IDS sequences from Hemiptera species The predicted functions are shown in colored rectangles (DPPS, decaprenyl diphosphate synthase; GGPPS, geranylgeranyl diphosphate synthase; FPPS, farnesyl diphosphate synthase). The maximum-likelihood tree is drawn to scale, with branch lengths measured in the number of substitutions per site (scale on the bottom left). Bootstrap values (1,000 replicates) are shown next to nodes. We included all *P. citri trans*-IDS candidates, except for *trans*IDS17, a predicted ubiquitin thioesterase, and examples of IDS sequences from Hemiptera species, which were characterized *in vitro.* The latter include sequences from aphids (*Megoura viciae*,^32^^,^^33^*Aphis gossypii*,^31^*Myzus persicae*,^34^ and *Acyrthosiphon pisum*^35^) and from shield bugs (*Halyomorpha halys*,^36^*Nezara viridula*,^36^ and *Murgantia histrionica*.^37^ See Figure S9 for the alignment of *P. citri trans*-IDS sequences included in the phylogenetic tree.

We further analyzed the sequence features and phylogeny of identified *P. citri* IDS sequences, which suggested their potential functions (Table 1; Figure 2 for *trans*-IDS candidates). Two candidate sequences (*trans*IDS2, *trans*IDS4) are similar to decaprenyl diphosphate synthases (DPPSs), long-chain IDSs that catalyze the formation of the ubiquinone prenyl sidechain, important in aerobic cellular respiration. The functional DPPS enzyme forms a heterodimer of subunits 1 and 2, of which subunit 1 has a higher sequence homology to IDSs, while subunit 2 acts as a regulatory subunit.^31^^,^^32^ T*rans*IDS2 is similar to sequences of DPPS subunit 1 (Figures 2 and S5), while *trans*IDS4 lacks the D-rich motifs and is similar to sequences of DPPS subunit 2 (Table 1, and Figure S10).

**Table 1:** *P. citri cis*- and *trans*-IDS candidates

Candidate	FARM	SARM	Putative function	logFC	R	I
*trans*IDS2	DDVID	DDLLD	DPPS subunit 1	0.18	Y	N
*trans*IDS3	DDIID	DDYLD	FPPS	1.20∗	Y	N
*trans*IDS4	/	/	DPPS subunit 2	0.32	N	N
*trans*IDS5	DDIMD	DDYLD	FPPS	0.22	Y	Y
*trans*IDS8	DDILD	NDYND	FPPS-like	−1.26	/	/
*trans*IDS9	DDIFD	NDYND	FPPS-like	3.11∗	/	/
*trans*IDS10	DDAID	NDYCD	FPPS-like	0.93∗	N	N
*trans*IDS11	DDALD	NDYYD	FPPS-like	1.61∗	Y	N
*trans*IDS12	DDIAD	NDGVAL	FPPS-like	5.49∗	N	N
*trans*IDS13	DDVVD	NDGFVL	FPPS-like	−1.22∗	/	/
*trans*IDS16	DDIQD	DDYCN	GGPPS	0.08	N	N
*trans*IDS17	DDFHD	/	ubiquitin thioesterase	−0.13	Y	N
*trans*IDS18	DDALD	NDYYD	FPPS-like	−0.38	/	/
*cis*IDS1	/	/	DHPPS	0.07	N	N
*cis*IDS2	/	/	DHPPS-like	1.90	/	/
*cis*IDS3	/	/	DHPPS-like	0.09	/	/
*cis*IDS5	/	/	DHPPS-like	0.04	/	/
*cis*IDS8∗	/	/	UPPS	NA	N	N
*cis*IDS9	/	/	DHPPS regulatory subunit	0.15∗	/	/

For each candidate sequence, its putative function, differential expression values (logFC), contrasting samples from virgin to mated females, and results of *in vitro* regular (R) and irregular (I) C10 or C15 coupling activity tests of the proteins expressed in *E. coli* are given. For *trans*-IDS candidates, amino acids positioned at the first and second D-rich motif (FARM, SARM) according to the multiple sequence alignment are given as well. Conserved aspartate (D) residues are underlined. LogFC (log2-fold changes) values were obtained by averaging logFC values for all sequences in the sequence cluster of each candidate (Table S4C) and are marked with an asterisk if the differential expression was statistically significant (pAdj >0.05) for at least one of the sequences in each cluster. "/" – activity test not performed. Asterisk in the "Candidates" column denotes the sequence originating from *Candidatus* Moranella endobia. DPPS – decaprenyl diphosphate synthase, FPPS – farnesyl diphosphate synthase, GGPPS – geranylgeranyl diphosphate synthase, DHPPS – dehydrodolichyl diphosphate synthase.

*Trans*IDS3 and *trans*IDS5 have high similarity to FPPSs (Table 1; Figures 2 and S6). Additionally, there are seven sequences (*trans*IDS8, *trans*IDS9, *trans*IDS10, *trans*IDS11, *trans*IDS12, *trans*IDS13, *trans*IDS18) with similarity to FPPS-like sequences from other Coccoidea species (Figure S11). All seven of them have a canonical FARM (DDxxD), but not SARM (Table 1). Phylogenetically, the FPPS-like sequences are separated from the mealybug FPPS sequences with low sequence similarity between the two groups (Figure S11). Similar diversification pattern of IDS genes within Coccomorpha has been indicated previously.^36^ *Trans*IDS3 and *trans*IDS5, as well as *trans*IDS12 and *trans*IDS13 also have high pairwise sequence similarity (78% and 75%, respectively), indicating a possibility of more recent duplication events.

*Trans*IDS16 is similar to geranylgeranyl diphosphate synthases (GGPPS), catalyzing the formation of the diterpenoid precursor (Figures 2 and S12). Sequences of *trans*IDS1, *trans*IDS19, and *trans*IDS20 do not have canonical D-rich motifs. Additionally, *trans*IDS1 is similar to E3 ubiquitin protein ligases, while we could not find any similarity matches for *trans*IDS19 and *trans*IDS20. *Trans*IDS17 is similar to otubain-like ubiquitin thioesterases (Figure S13) and includes a DDxxD motif aligning with FARM, as do all other otubain-like orthologs identified from mealybugs and included in the phylogenetic analysis (Figure S13).

Out of the *cis*-IDS candidates, *cis*IDS1, *cis*IDS2, *cis*IDS3, and *cis*IDS5 are homologous to the catalytic subunit of heterodimeric dehydrodolichyl diphosphate synthase (DHPPS) (Figures S7 and S14), involved in the biosynthesis of dolichol phosphate, the lipid carrier of glycosyl units used for N-glycosylation in eukaryotes. Candidates *cis*IDS2, *cis*IDS3, and *cis*IDS5 have some closer orthologs within the Coccoidea, however, they are very distant to other DHPPS sequences and even *cis*IDS1 (Figure S14). On the other hand, *cis*IDS9 has highest similarity to sequences of the DHPPS regulatory subunit (Figure S8).

### Regular IDS activity was shown for five and irregular activity for one *P. citri* candidate

With the aim of identifying enzymes involved in the synthesis of the irregular C10 sex pheromone, we tested the activity of *P. citri* IDS candidates for producing short polyprenyl chains. We selected 11 *P citri* candidate sequences with homology to *trans*- or *cis*-IDSs for which the full sequence was confirmed with long-read transcriptome sequencing (Tables 1 and S4C). Two of these sequences (*trans*IDS3 and *trans*IDS10) were synthesized according to long-read sequencing data, and nine sequences (*trans*IDS2, *trans*IDS4, *trans*IDS5, *trans*IDS11, *trans*IDS12, *trans*IDS16, *trans*IDS17, *cis*IDS1, and *cis*IDS8) were amplified from *P. citri* cDNA (Table S4C). All sequences were expressed in *E. coli* and purified recombinant proteins were tested *in vitro* to identify regular and irregular IDS catalytic activity forming C10 or C15 backbones (see Figure S15 for SDS-PAGE of expressed and purified candidates). For *trans*IDS4 and *trans*IDS12, two amplicons were obtained, cloned, and tested (Figures 1A and 1D, and Table S4C).

**Figure 3:**
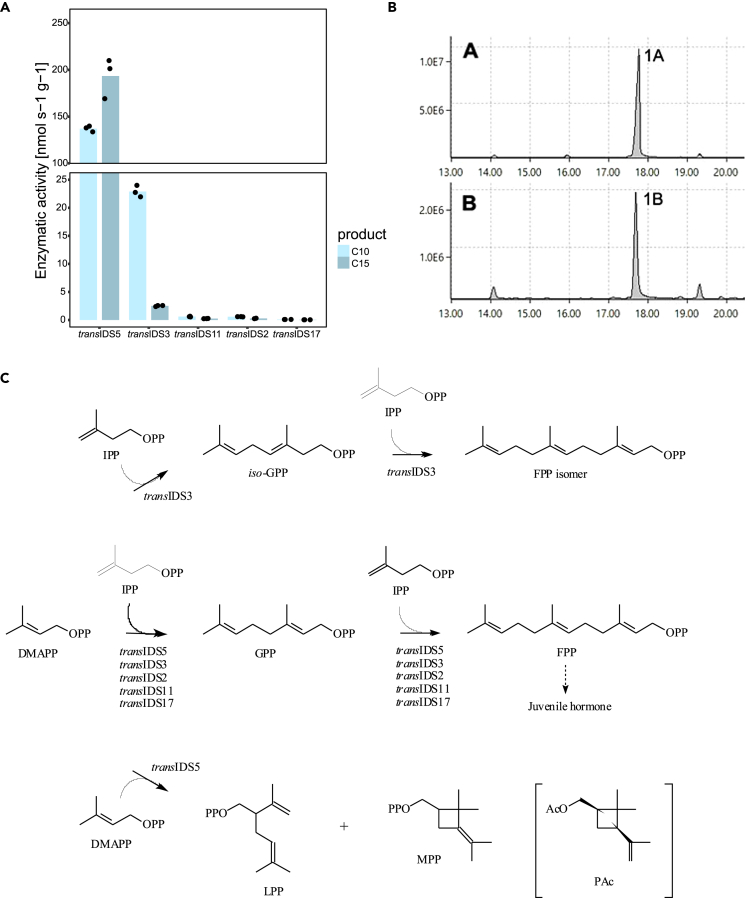
*In vitro* activity of *P. citri* IDS sequences expressed in *E. coli* Measured enzymatic activity for production of C10 and C15 prenyl diphosphates (A), chromatogram of standard geraniol (peak 1A in upper panel) and *trans*IDS5 product (peak 1B in lower panel) (B), and schematic representation of confirmed coupling reactions performed by *P. citri trans*-IDS candidates (C). *Trans*IDS5, *trans*ID11, *trans*IDS2, and *trans*IDS17 produced C10 geranyl diphosphate (GPP) and C15 farnesyl pyrophosphate (FPP); *trans*IDS3 produced C10 GPP and *cis*-isogeranyl diphosphate and two C15 FPP isomers. (A) Activity for production of C10 prenyl diphosphates (light blue bars) and activity for production of C15 prenyl diphosphates (dark blue bars). Height of the bars represents the mean of three separate measurements, plotted as block dots. Raw data are available in Table S5. Note the y axis break due to the much higher activity of *trans*IDS5. We did not detect any C10 or C15 products with other tested candidates (Table 1). Chromatograms in (B) are also shown in Figure S16. MS spectra of highlighted peaks are given in Figure S17. Chromatograms and MS spectra of products obtained with other candidates are available in Figures S16–S27. In (C), dimethylallyl diphosphate (DMAPP) can be joined to its isomer isopentenyl diphosphate (IPP) to form regular geranyl and iso-geranyl diphosphates (GPP and iso-GPP) and isomers of farnesyl diphosphate (FPP). FPP is a precursor for juvenile hormone biosynthesis, as denoted by the dashed arrow. Alternatively, two DMAPP units can be assembled into irregular lavandulyl diphosphate (LPP) and maconellyl diphosphate (MPP). The structure of planococcyl acetate (PAc), the sex pheromone of *P. citri*, is shown in brackets and is presumed to result from the coupling of two DMAPP units as well.

Five *trans*-IDS-like proteins (*trans*IDS2, *trans*IDS3, *trans*IDS5, *trans*IDS11, and *trans*IDS17) displayed regular IDS activity (Table 1, and Figure 3A). The products of *trans*IDS2, *trans*IDS5, *trans*IDS11, and *trans*IDS17 were identified as geranyl and farnesyl diphosphates based on chromatographic behavior and mass spectra of diphosphates and their dephosphorylated alcohol derivatives (Figures 3A, 3C, and S16–S24). Candidate *trans*IDS3 had an unusual pattern of regular prenyl diphosphate products. It formed two isomers of C10 prenyl diphosphate, geranyl and *iso*-geranyl diphosphates, that were further elongated to two C15 isomers: farnesyl and *iso*-farnesyl diphosphate (Figures 3C and S16–S21). Both isomers were identified based on the MS reference library spectra. Specific activities of *trans*IDS5 were significantly higher than those of the other enzymes, with *trans*IDS3 also exhibiting a relatively high activity for the production of C10 prenyl diphosphates (Figure 3A, and Table S5). Both *trans*IDS3 and *trans*IDS5 sequences were expressed in *P. citri* females with *trans*IDS3 also differentially expressed between virgin and mated females with higher expression in virgin females (Tables 1 and S4C).

Moreover, candidate *trans*IDS5 also had a low level of irregular IDS activity, if supplied with DMAPP only (Figure 3C). The enzyme produced two compounds, which were identified as lavandulyl (LPP) and maconelliyl diphosphate (MPP), based on GC-MS data of their dephosphorylated derivatives (Figures S25–S27).

To assess the activities of candidate proteins *in vivo* in the eukaryotic cell environment, the candidate IDS sequences were transiently expressed in the plant heterologous expression model *Nicotiana benthamiana*. Plants utilize two separately compartmentalized pathways for production of C5 isoprene blocks IPP and DMAPP. Mevalonate (MVA) pathway is localized in the cytoplasm, while MEP (methylerythritol phosphate) pathway operates in chloroplasts. Regular monoterpenes in plants derive mainly from the MEP pathway, although the cross-talk between the precursor pools is possible.^38^ The known irregular IDS enzymes of plant origin (CPPS from *Tanacetum cinerariifolium* and LPPS from *Lavandula x intermedia*) are localized to chloroplasts.^11^^,^^13^ In our experiments, the deletion of chloroplast-targeting peptide from these enzymes resulted in activity loss in plant cells. Therefore, each candidate gene was tested in its native form and with the addition of a chloroplast transit peptide. HPLC-MS analysis of leaf extracts did not reveal irregular prenyl diphosphates. Similarly, no production of volatile organic compounds derived from irregular monoterpenoids was detected by GC-MS in agroinfiltrated *N. benthamiana* leaves. Regular isoprenyl diphosphate synthase activity of candidate proteins could not be assessed in this system due to the presence of native plant GPP and FPP interfering with the detection of additional GPP and FPP possibly formed by heterologously expressed enzymes.

### Mutagenesis of *trans*IDS5 active site revealed functional importance of active site residues for its enzymatic activity

We further set to identify the amino acid residues important for the irregular coupling activity of *trans*IDS5 by performing mutagenesis. It was suggested that replacement of the first aspartate (D) in SARM with asparagine (N) played an important role in the evolution of irregular coupling activity.^39^ However, in *trans*IDS5, analogous mutation (D308N) caused a complete loss of irregular activity. The same was observed after the exchange of two other aspartate residues in SARM (D309N and D312N), and the first aspartate in FARM (D166N).

**Figure 4:**
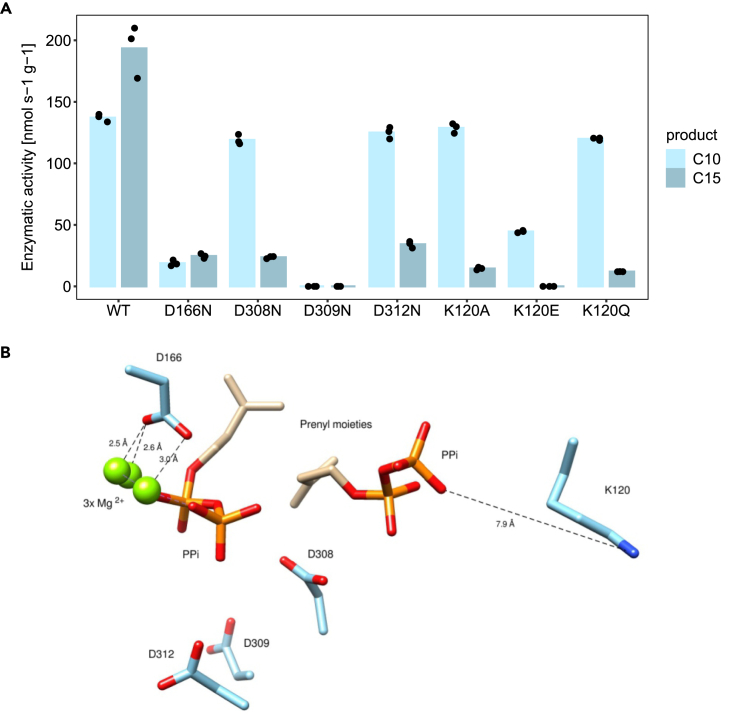
Mutagenesis of *trans*IDS5 active site residues (A) Activity of *trans*IDS5 and its mutants expressed in *E. coli.* Activity for the production of C10 geranyl diphosphate (GPP, light blue bars) and activity for the production of C15 farnesyl diphosphate (FPP, dark blue bars) are plotted. Height of the bars represents the mean of three separate measurements, plotted as black dots. Raw data are available in Table S5. (B) Mutated amino acids and their position in the active site, based on a computational model of active center of *trans*IDS5 with two bound substrate (IPP) molecules showing the aspartate of the first (D166) and the second (D308, D309, D312) aspartate-rich motifs and lysine 120 (K120). Oxygen atoms in aspartate side chains and in pyrophosphate moieties (PPi) of the substrates are shown in red; the nitrogen atom of lysine side chain is highlighted in blue.

Moreover, the regular activity of aspartate mutants was affected to different degrees (Figure 4A). The regular activity of D166N mutant was significantly diminished, although the ratio between C10 GPP and C15 FPP was similar to the wild-type (WT) *trans*IDS5 (Figure 4A). D308N and D312N substitutions had an impact on the regular product length. Both mutants performed the initial coupling of IPP and DMAPP to C10 chains at levels comparable to the WT enzyme, but the addition of the second IPP to form the C15 chain was hampered compared to the WT (Figure 4A). The regular activity was completely abolished after the D309N exchange (Figure 4A).

To find additional residues important for the catalytic activity of *trans*IDS5, we build a computational model of its 3D structure (Figure 4B). We identified a positively charged lysine (K) residue in position 120 located at a comparatively short distance from the negatively charged diphosphate moiety of the IPP substrate (Figure 4B). We exchanged this residue with alanine (K120A), polar uncharged glutamine (K120Q), and negatively charged glutamate (K120E). All three substitutions resulted in the loss of irregular coupling activity, as well as in the diminished elongation of GPP (C10) to FPP (C15). As expected, the K120E exchange, introducing a negative charge in the active site, had the most inhibiting effect, precluding FPP formation entirely (Figure 4A).

### Heteromeric associations did not change IDS activity

In most cases, *trans*- and *cis*-IDSs are active in the form of homodimers.^40^ However, some IDSs form heterodimers with non-catalytic subunits, which modulate or stimulate their activity.^18^^,^^41^^,^^42^^,^^43^^,^^44^^,^^45^^,^^46^ To determine if irregular coupling might depend on the presence of other subunits, we tested *P. citri* IDS candidates in combinations with potential interactors (Table S6). In the *P. citri* genome, we identified homologs of the DPPS and DHPPS regulatory subunits (*trans*IDS4 and *cis*IDS9, respectively) and both were combined with all IDS-like candidates in irregular activity assays (Table S6). In addition, we examined two *P. citri* non-IDS-like proteins for their IDS-modifying activities: the protein product of g23689.t1, annotated as a pheromone-binding protein (PBP) and highly expressed in virgin females (Table S3B), and g36424.t1, identified as a *P. citri* homolog of isopentenyl diphosphate isomerase (IDI), an enzyme catalyzing the reversible isomerization of IPP to DMAPP (Table S6). Addition of *P. citri*-IDI to the reaction with IDS candidates supported the formation of regular products, if either IPP or DMAPP only were added to the reaction. However, none of the tested putative regulatory subunits altered or spurred irregular coupling activity of IDS candidates.

We also tested the hypothesis that heterodimeric complexes consisting of two IDS subunits are required for irregular coupling. Activity assays with DMAPP as the only substrate were carried out with pairwise combinations of *P. citri* IDS candidate proteins with each other as well as with IDS proteins forming irregular prenyl diphosphates (Table S6). The latter allows for the detection of terpene synthase (TPS) activities of *P. citri* IDS candidates, converting the prenyl diphosphate substrates into corresponding prenyl alcohols. Combined assays did not reveal any changes to the enzymatic activities of IDS candidates, which also did not demonstrate TPS activity on the tested irregular prenyl diphosphates.

## Discussion

Sustainable insect pest management, supported by state-of-the-art knowledge and technology, is an integral part of sustainable food production in a changing climate, in which the geographical ranges of many insect species are expected to widen.^47^ However, the use of biotechnology and genetic solutions depend on the detailed information about gene function. In this work, we present the results of a comprehensive search for candidate genes coding for isoprenyl diphosphate synthase (IDS) activity from the citrus mealybug, *P. citri*. The identification of these genes has the potential to enable either biological production of *P. citri* sex pheromones or gene silencing approaches, both of which provide novel options for pest management (reviewed in the study by Mateos Fernandez et al.^48^).

Functional genomics of many insect species is hindered by the lack of well-annotated, high-quality genome sequences.^49^^,^^50^ To improve the available genetic resources for *P. citri*,^26^ we complemented the existing genome data with short- and long-read transcriptome data (Figure S1). The transcriptomic dataset enabled us to predict and amplify alternative transcript sequences compared to the available genome models (Figures 1 and S4). In addition, we found a novel coding sequence (*cis*IDS5) among the *de novo* assembled transcripts, for which we were unable to find an aligning gene model in the current *P. citri* genome assembly. Using all resources, we were able to identify 18 candidate *P. citri* IDS sequences. Among these were putative DHPPS, FPPS, FPPS-like, GGPPS, DPPS, and DPPS-like coding sequences, as well as a ubiquitin thioesterase-like sequence with a D-rich motif (Table 1). Apart from the search for IDS coding sequences, our virgin and mated female-specific dataset could be useful for the identification of other genes related to mating. Among the differentially expressed genes, we were for example able to identify a putative pheromone-binding protein (g23689.t1).

However, inferring function from sequence analyses alone is inconclusive for IDSs, as even small changes can alter substrate and chain-length specificity.^51^ To test the activity of IDS candidates, we focused on the detection of C10 and C15 coupling products. We, therefore, did not confirm if the predicted DHPPS, GGPPS, and DPPS had longer chain-producing activities. *Trans*IDS16, a predicted GGPPS did not produce any C10 or C15 products. Similarly, neither of the two tested *cis*-IDS-like proteins produced detectable levels of regular or irregular C10 or C15 backbones, as both were predicted to be long-chain IDSs, namely DHPPS (*cis*IDS1) and UPPS (*cis*IDS8) (Table 1). Interestingly, *trans*IDS2, a predicted long-chain IDS (DPPS catalytic subunit), produced low amounts of FPP and GPP (Figure 3A). This might be attributed to functional promiscuity *in vitro*, producing polyprenyl chains of different lengths, as reported for other IDS enzymes.^9^ Additionally, *trans*IDS17, which was not homologous to IDS sequences but rather to otubain-like ubiquitin thioesterases, was able to produce regular C10 and C15 backbones, albeit at the lowest rate of all candidates (Figure 3A). It remains unknown if otubain-like sequences from other mealybug species have IDS activity, and whether this activity has a biological function.

The two putative FPPS sequences (*trans*IDS3 and *trans*IDS5) both displayed regular coupling activity resulting in C10 and C15 units. Our enzymatic activity and gene expression results indicate that *trans*IDS5 might be the major source of C15 terpenes in *P. citri*, while the C10 could be mainly composed of *trans*IDS5 and possibly also *trans*IDS3 products. The highest specific activity was measured for *trans*IDS5, also producing low amounts of irregular prenyl diphosphates, LPP and MPP (Figure 3C), which have not been reported in *P. citri*. The 2-methylbutanoated versions of both compounds have been identified as the sex pheromone of *Maconellicoccus hirsutus*, the pink hibiscus mealybug.^52^ Esterified lavandulol compounds act as sex pheromones also in some other *Planococcus*, *Pseudococcus*, and *Dysmicoccus* species.^3^ However, the irregular coupling activity of *trans*IDS5 only takes place in the absence of IPP and might, therefore, be unlikely to occur *in vivo* where the presence of IPP is expected. Although we did not find conclusive evidence for the role of *trans*IDS5 in the biosynthesis of the *P. citri* sex pheromone, we propose that it is the main source of regular sesquiterpenes in *P. citri*, possibly including the juvenile hormone (Figure 3C). Using structure-informed mutagenesis, we additionally demonstrated the importance of K120, D166, D308, D309, and D312 for the irregular coupling mechanism observed for *trans*IDS5 *in vitro* (Figure 4A).

Terpenoids are known for their extensive evolutionary divergence, with many instances of lineage-specific pathways and, therefore, metabolites. Despite the occurrence of many common and unique terpenoids in animals, terpenoid diversification is better understood in plants and microbes.^2^^,^^53^ In plants, terpenoid diversity is mainly determined by the activity of TPSs, which have high evolutionary divergence and functional plasticity.^54^ However, sequences with homology to plant and microbial TPSs have not been identified in insect genomes. Instead, numerous instances of IDS gene-family expansion have been observed, with frequent gene duplications resulting in functional redundancy or neofunctionalization.^36^^,^^37^^,^^55^^,^^56^^,^^57^ Among the *P. citri* IDS sequences, we observed expansions of FPPS-like sequences and DHPPS-like sequences (Figures S11 and S14). The former extends the previously reported FPPS-like diversification within the Coccomorpha.^36^ Three FPPS-like sequences were successfully cloned and tested (*trans*IDS10, *trans*IDS11, and *trans*IDS12), however, only one candidate (*trans*IDS11) exhibited IDS activity, producing low amounts of GPP and FPP (Figure 3A). Interestingly, while we were not able to confirm GPPS or FPPS activity for *trans*IDS12, its expression was observed to be strongly upregulated in virgin compared to mated *P. citri* females, which was also true for *trans*IDS9, *trans*IDS10, and *trans*IDS11 (Table 1). This differential expression suggests a possible role in mating or reproduction and needs further investigation.

Despite detecting two irregular prenyl diphosphate products, none of the IDS-like candidates that we tested displayed coupling activity leading to *cis*-planococcyl diphosphate under our experimental conditions. Another option, which could be explored in the future, is an expression of candidate genes in insect cells, as their cellular environment should be closer to *in vivo* conditions and could contribute to correct folding and glycosylation of the holoenzyme. *In vitro* functional characterization of IDS enzymes can be unreliable, as activity can depend on many factors, including the formation of heteromers.^31^ To that end, we tested for modulating activity of several potential interactors but did not detect any changes in the activity of *P. citri* IDS candidates.

The model of irregular terpenoid biosynthesis via a genome-encoded IDS has been challenged. Alternative hypotheses include the possibility that insects sequester plant-derived compounds or utilize products of their endosymbionts’ metabolism. Many studies reveal a straightforward strategy of modification and accumulation of plant defense compounds,^29^ but, in some cases, the derivatives of plant secondary metabolites are also used in sexual communication. For example, fragments of plant pyrrolizidine alkaloids and phenylpropanoid metabolites are reported to make danaine butterfly males more attractive to the co-specific females.^58^ However, the presence of irregular monoterpene structures has not been reported for known *P. citri* host plants, which makes the sequestration hypothesis improbable. Like other insects feeding on nutritionally unbalanced plant sap, mealybugs rely on obligate endosymbiotic bacteria as a source of amino acids and vitamins.^59^ *P. citri* is a part of a nested tripartite endosymbiotic system, where the insect harbors the Betaproteobacteria *Candidatus* Tremblaya princeps, which contains the Gammaproteobacteria *Candidatus* Moranella endobia.^60^^,^^61^ Although we were unable to identify any short-chain IDS-coding sequences within the genomes of *P. citri* endosymbionts, we cloned and tested one IDS sequence, a putative UPPS, from *Candidatus* Moranella endobia (*cis*IDS8). However, this did not produce any regular or irregular short prenyl chains. Moreover, genetic crosses between *P. citri* and *Planococcus minor*—the latter producing a branched lavandulol-type monoterpene—indicate that the enzyme responsible for the formation of the irregular terpene skeleton is encoded in the insect nuclear genome and is present at a single locus.^62^ The structure of the sex pheromone is also not inherited maternally,^62^ as would be expected if synthesis requires the transfer of egg-transmitted endosymbionts.

Another possibility is that the mealybug sex pheromones are biosynthesized via a different catalytic mechanism, executed by a different, and as yet unidentified, class of enzymes. Novel terpene synthases have been described, expanding on the canonical models of terpene biosynthesis.^63^ A recent example is the elucidation of the iridoid pheromone found in the pea aphid, *Acyrthosiphon pisum*. Biosynthesis of this molecule proceeds through the same intermediates as in plants, however, it was shown to involve a set of enzymes that lack homology to the plant counterparts.^64^ The insect enzymes were identified by transcriptomic analysis of the female hind legs, the site of pheromone biosynthesis.^64^ Targeted transcriptome analyses of pheromone glands were also useful for determining the genes related to pheromone metabolism and transport in Lepidoptera species.^65^^,^^66^^,^^67^^,^^68^ At present, however, such approaches are not possible in *P. citri* as the site of the pheromone gland remains unknown.

The identification of the *P. citri* IDS gene family provides an important foundation for deciphering terpenoid biosynthesis in this species as well as other mealybugs. The candidate FPPS and FPPS-like sequences with confirmed regular and irregular activity might also present a starting point for protein engineering; mutations have been shown to increase or even spur irregular coupling activity in IDSs.^14^^,^^69^ Diversification of IDS sequences in Coccoidea is an intriguing genomic enigma for future investigation, especially in relation to their capacity for species-specific irregular monoterpene synthesis, a unique feature in this insect superfamily, which still remains elusive.

### Limitations of the study

Whole insects were used for RNA isolation and generation of long and short-read sequencing data, therefore the differential expression and transcript data encompasses status on whole organism level, obscuring tissue- or cell-type-specific changes/abundances. For more precise focus on pheromone synthesis-related changes, pheromone gland transcriptome would be of use, however, its location has not been determined in *P. citri*. For the characterization of IDS activity, we used an experimental setup that allowed only for the identification of C10 and C15 chains, therefore we did not confirm any long-chain IDS activity, which needs to be characterized in the future.

## STAR★Methods

### Key resources table

**Table:** 

REAGENT or RESOURCE	SOURCE	IDENTIFIER
**Bacterial and virus strains**		

*Escherichia coli* TOP10 strain	Invitrogen	N/A
*Escherichia coli* BL21(DE3)pLysS	N/A	N/A
*Agrobacterium tumefaciens* EHA105 strain	N/A	N/A

**Chemicals, peptides, and recombinant proteins**		

TRIzol Reagent	ThermoFisher Scientific	Cat# 15596026
Amylose resin	New England Biolabs	E8022
ZB-5MS fused silica capillary column	Phenomenex	N/A
CycloSil-B column	Agilent	112-6632
Zorbax 300Extend-C18	Agilent	763973-902
Calf intestinal alkaline phosphatase	Promega	M1821
Isopentenyl diphosphate (IPP)	Echelon Biosciences	I-0050
Dimethylallyl diphosphate (DMAPP)	Echelon Biosciences	I-0051
Geranyl diphosphate (GPP)	Echelon Biosciences	I-0100
Poroshell 120 EC-C18 column (3.0 x 50 mm, 2.7 μm)	Agilent Technologies	699675-742

**Critical commercial assays**		

Quant-iT RNA Assay Kit	ThermoFisher Scientific	Cat# Q33140
TruSeq RNA Library Prep Kit v2	Illumina	RS-122-2001

**Deposited data**		

Structure of *S. cerevisiae* geranylgeranyl pyrophosphate synthase in complex with magnesium and IPP	Guo et al.^70^	PDB: 2E8U
*Planocococcus citri* virgin and mated females Illumina short reads	This paper	GEO: GSE179660
*Planocococcus citri* virgin females Iso-Seq reads	This paper	SRA: SRR15093694
*Planocococcus citri* consolidated transcriptome	This paper	FAIRDOMHub, https://fairdomhub.org/data_files/6316/content_blobs/17124/download
*Planocococcus citri* consolidated transcriptome annotation file	This paper	FAIRDOMHub, https://fairdomhub.org/data_files/6317/content_blobs/17125/download
*Planocococcus citri* Illumina short reads	University of Edinburgh	SRA: SRR11260462, SRR11260463, SRR11260468, SRR11260469, SRR11260470,SRR11260471
*Planococcus citri* v1 genome assembly	Dominic Laetsch^26^	https://ensembl.mealybug.org/Planococcus_citri_pcitriv1/Info/Index
*Planococcus ficus* v0 genome assembly	Filip Husnik^71^	https://ensembl.mealybug.org/Planococcus_ficus_pficusv0/Info/Index
*Pseudococcus longispinus* v1 genome assembly	Dominic Laetsch^72^	https://ensembl.mealybug.org/Pseudococcus_longispinus_v1/Info/Index
*Maconelicoccus hirsutus* genomic data	Kohli et al.^73^	https://github.com/VBL-Epi/M_hirsutus

**Recombinant DNA**		

pMAL-c5X expression vector	New England Biolabs	N8108S

**Software and algorithms**		

CLC Genomics Workbench 10.0.1	Qiagen	N/A
R	R Core Team	https://www.r-project.org/
*edgeR* R package	Robinson et al.^74^	https://bioconductor.org/packages/release/bioc/html/edgeR.html, RRID:SCR_012802
*limma* R package	Ritchie et al.^75^	https://bioconductor.org/packages/release/bioc/html/limma.html, RRID:SCR_010943
Minimap2	Li et al.^76^	https://github.com/lh3/minimap2, RRID:SCR_018550
cDNA_cupcake	Elizabeth Tseng^77^	https://github.com/Magdoll/cDNA_Cupcake
BBTools	Brian Bushnell^78^	https://jgi.doe.gov/data-and-tools/software-tools/bbtools/, RRID:SCR_016968
rnaSPAdes v3.13.0	Bushmanova et al.^79^	https://cab.spbu.ru/software/rnaspades/, RRID:SCR_016992
rnaQUAST	Bushmanova et al.^80^	https://github.com/ablab/rnaquast, RRID:SCR_016994
BUSCO v5.4.3	Manni et al.^28^	https://busco.ezlab.org/, RRID:SCR_015008
matchAnnot	Tom Skelly^81^	https://github.com/TomSkelly/MatchAnnot
Transdecoder v5.5.0	Brian J. Haas^82^	https://github.com/TransDecoder/TransDecoder, RRID:SCR_017647
InterProScan v5.56-89.0	Jones et al.^83^	https://github.com/ebi-pf-team/interproscan
mmseqs2	Mirdita et al.^84^	https://github.com/soedinglab/MMseqs2, RRID:SCR_022962
Pavian	Breitwieser et al.^85^	https://github.com/fbreitwieser/pavian, RRID:SCR_016679
MEME Suite	Bailey et al.^86^	https://meme-suite.org/meme/, RRID:SCR_001783
blast	Altschul et al.^87^	https://blast.ncbi.nlm.nih.gov/Blast.cgi, RRID:SCR_004870
CD-HIT	Fu et al.^88^	https://sites.google.com/view/cd-hit, RRID:SCR_007105
SWISS-MODEL	Waterhouse et al.^89^	https://swissmodel.expasy.org/, RRID:SCR_018123
MEGA X	Kumar et al.^90^	https://www.megasoftware.net/, RRID:SCR_023017
STAR v2.7.5c	Dobin et al.^91^	https://github.com/alexdobin/STAR, RRID:SCR_004463
BioRender	N/A	https://www.biorender.com/

### Resource availability

#### Lead contact

Additional information and requests for resources and reagents used in this study should be directed to and will be fulfilled by the lead contact, Mojca Juteršek, mojca.jutersek@nib.si.

#### Materials availability

Constructed plasmids for bacterial and plant expression are available upon reasonable request. For the bacterial expression constructs, contact Iryna M. Gerasymenko (gerasymenko@bio.tu-darmstadt.de) and for plant expression constructs, contact Diego Orzáez (dorzaez@ibmcp.upv.es).

#### Data and code availability

• Raw Illumina reads from *P. citri* virgin and mated females, along with results of the differential expression analysis (as given in Table S3B as well), were deposited at GEO under accession GSE179660. Raw Iso-Seq reads were deposited at SRA under accession SRR15093694. *P. citri* short reads provided by the University of Edinburgh and used in the second *de novo* transcriptome assembly are deposited at SRA under accession numbers SRR11260462, SRR11260463, SRR11260468, SRR11260469, SRR11260470, and SRR11260471. *P. citri* consolidated transcriptome FASTA file is available at FAIRDOMHub (https://fairdomhub.org/data_files/6316/content_blobs/17124/download), together with an annotation file (https://fairdomhub.org/data_files/6317/content_blobs/17125/download), also included in the Supplemental Files (Table S3A). All listed datasets are publicly available as of the date of publication. All accession numbers and links are given in the Deposited Data section of the key resources table. All other data presented in this paper is given in the supplemental information.
• This paper does not report original code. All software packages and code were run with standard parameters, unless otherwise stated in STAR Methods.
• Any additional information required to reanalyze the data reported in this paper is available from the lead contact upon request.

### Experimental model and study participant details

#### Insect rearing and sampling

The stock colony of *P. citri* was established in the insectary facilities at the Universitat Politècnica de València (UPV, Valencia, Spain) using specimens from Servei de Sanitat Vegetal of Generalitat Valenciana (GVA, Valencia, Spain). Mealybugs were reared on organic green lemons, maintained in a rearing chamber, in the dark at 24 ± 2°C, with 40−60 % relative humidity. Mated females were allowed to oviposit and, when ovisacs were laid, they were gently transferred with an entomological needle to new lemons. To obtain samples of virgin females, some lemons were separated from the main colony and were visually inspected every 3−4 days for the presence of male cocoons, which were manually removed with an entomological needle to leave only virgin females. Mated females were sampled on the lemons from the main stock colony after checking for the presence of ovisacs. The production of sex pheromone was confirmed by collection of emitted volatiles from a group of 20−30 virgin females by solid phase microextraction technique (SPME).^92^ The identity of the sex pheromone was unequivocally confirmed by full coincidence of its mass spectra and retention time with an analytical standard sample synthesised according to the method described by Kukovinets et al., 2006.^93^

### Method details

#### RNA isolation

RNA was isolated from approximately 150 (approximately 100 mg) pooled virgin and mated *P. citri* females separately, using TRIzol Reagent (Thermo Fisher Scientific, Waltham, MA, USA) according to the manufacturer’s instructions. RNA was isolated from four biological replicates, resulting in eight RNA samples, four from pools of virgin females and four from pools of mated females. RNA quality was assessed using the Quant-iT™ RNA Assay Kit **(**Thermo Fisher Scientific, Waltham, MA, USA).

#### Short-read Illumina RNA-Seq

Approximately 1 μg of isolated RNA from all eight RNA samples was purified to extract polyadenylated mRNA using biotin beads, fragmented, and primed with random hexamers to produce the first cDNA strand followed by second-strand synthesis to produce double-stranded cDNA. Libraries were constructed at the Earlham Institute, UK, on a Sciclone G3 NGSx workstation (PerkinElmer, Waltham, MA, USA) using the TruSeq RNA protocol v2 (Illumina Part # 15026495 Rev.F) and sequenced on two lanes of a HiSeq 4000 (Illumina, San Diego, CA, USA) generating 150 bp negative strand-specific paired-end reads. Illumina reads were subjected to adapter trimming, read quality filtering, mapping, and read summarization (counting only uniquely mapped paired reads) in CLC Genomics Workbench 10.0.1 (Qiagen, Hilden, Germany), with mapping parameters: mismatch cost 2, insertion cost 3, deletion cost 3, length fraction 0.9, similarity fraction 0.9. Reads were mapped to the *Pcitri*.v1 genome, downloaded from the MealyBugBase.^26^ Differential expression analysis was performed with R packages edgeR and limma,^74^^,^^75^^,^^94^ contrasting samples from virgin and mated *P. citri* females.

#### Long-read Iso-Seq transcriptome

Approximately 2 μg of RNA isolated from pooled virgin *P. citri* females was sent for PacBio SMRT sequencing using the Iso-Seq protocol (National Genomics Infrastructure, Sweden). High and low quality full-length transcript isoforms were mapped to the *Pcitri*.v1 genome using minimap2.^76^ Based on the genome mapping, the isoforms were collapsed and representative isoforms were filtered to exclude isoforms with 5' artefacts, all using scripts from cDNA_cupcake Github repository.^77^

#### *De novo* short-read transcriptome assembly

Trimmed paired and orphan Illumina RNA-Seq reads were subjected to *de novo* transcriptome assembly using rnaSPAdes v3.13.0.^79^ Two assemblies were created, first, using the virgin and mated female *P. citri* paired-end short reads (2x150 nt) obtained in this study, and second, combining them with paired-end reads of seven samples of *P. citri,* kindly provided by Dr. Laura Ross at the University of Edinburgh (2x75 nt and 2x50 nt, see Data and code availability). For the first assembly, default rnaSPAdes parameters were used, whereas for the second assembly, adapter trimming and sequencing artefact removal was additionally performed using BBTools’ bbduk script,^78^ followed by SPAdes’ BayesHammer^95^ and BBTools’ ecco, ecc (6 passes) and tadpole read error correction scripts. Assembly was done using k-mer lengths 29 and 49.

#### Consolidation of sequence resources, annotation, and quality control

The two Illumina short read assemblies (Figures S1A and S1B) were combined into a single set (Figure S1C) by removing identical sequences with CD-HIT-EST v6.4^88^ using 100% sequence identity threshold. In a successive run, the combined short read transcriptome and the Iso-Seq transcriptome (combining mapped and unmapped representative isoforms, Figure S1D) were consolidated into a single set (Figure S1E), again using CD-HIT-EST at 100% sequence identity threshold. The final consolidated transcriptome dataset as well as both short- and long-read datasets were quality checked using rnaQUAST^80^ and their completeness was assessed using BUSCO v5.4.3^28^ with insecta_odb10 lineage dataset. The consolidated transcriptome set was mapped to the *Pcitri*.v1 genome using STARlong v2.7.5c^91^ and mapped transcripts were matched with the gene models and scaffolds using matchAnnot^81^ (version 20150611.02). Transcripts were also subjected to ORF prediction and translation using Transdecoder v5.5.0,^82^ followed by the annotation of the predicted protein sequences with PFAM and InterPro (IPR) IDs using InterProScan v5.56-89.0.^83^ The longest ORFs were further used for taxonomic classification using mmseq2 taxonomy tool,^84^ querying against the non-redundant NCBI protein database (downloaded on 19.07.2022). Results of taxonomic classification were visualised with Pavian.^85^ Illumina short reads were mapped back to both *de novo* assemblies as well as IsoSeq transcriptome using STAR v2.7.5c^91^ with default parameters. Short read mapping counts for all three datasets were used for differential transcript expression analysis based on R packages edgeR and limma.^74^^,^^75^^,^^94^

#### Candidate selection

We queried the IPR annotations of *Pcitri*.v1 gene models and our consolidated transcriptome dataset with IDS-related IPR IDs, namely IPR001441 (Decaprenyl diphosphate synthase-like family), IPR036424 (Decaprenyl diphosphate synthase-like superfamily) and IPR000092 (Polyprenyl synthetase family), to identify homologs of *cis*- and *trans*-prenyltransferases. Besides using the IPR search to determine *trans*-IDS homologs, we also conducted a motif search using MAST v5.0.1 in MEME Suite.^86^ Twenty-one sequences of farnesyl diphosphate synthases (FPPS) from different organisms were used as an input for MEME. The identified motifs were used as a query against the *Pcitri*.v1 proteins (Table S7). Additionally, we also searched for *P. citri* isopentenyl diphosphate isomerase (IDI) homologs by querying the *P. citri*.v1 protein sequences with *Nicotiana tabacum* IDI sequence (GenBank: NP_001313140) using blastp.^87^

All extracted sequences with homology to *cis*- and *trans*-IDSs longer than 100 amino acids were subjected to CD-HIT with 100% sequence identity threshold to remove duplicates. They were further grouped into clusters, using CD-HIT along with multiple sequence alignments with MUSCLE,^96^ and the sequence representing the most probable full-length coding sequence was selected within each cluster.

For the phylogenetic analyses of candidate sequences, we first searched for similar sequences using blastp against the non-redundant protein bases or tblastn against the transcriptome shotgun assembly sequences (TSA). Additionally, we searched for homologs from *Planococcus ficus* and *Pseudococcus longispinus* genome assemblies in the MealyBugBase,^71^^,^^72^ and from *Maconellicoccus hirsutus* transcriptomic resource.^73^ Amino acid sequences were aligned using the MUSCLE algorithm^96^ and used to construct maximum-likelihood phylogenetic trees with LG model^97^ tested with the bootstrap method at 1000 replicates in MEGA-X, version 10.0.5.^90^

#### Candidate sequence amplification, expression in *E. coli*, and protein purification

The candidate sequences were amplified from the cDNA prepared with oligoT primer (T15NNN) from total RNA isolated from virgin *P. citri* females using primers listed in Table S8 and cloned into pMAL-c5X expression vector (New England Biolabs, Ipswich, MA, USA) using *Nco*I/*Bam*HI sites. The proteins were expressed in BL21(DE3)pLysS *E. coli* cells. After transformation, single colonies were injected into 2 mL of LB medium containing 100 mg/L of ampicillin and 50 mg/L of chloramphenicol and incubated over night at 37°C. Bacterial suspensions were inoculated into fresh LB medium containing 100 mg/L of ampicillin (1 mL/100 mL) and incubated at 37°C till OD_600_ reached approximately 0.5. The expression was induced by 0.5 mM IPTG and carried out at 30°C overnight. The cells were collected by centrifugation (5 min at 5,000 g) and resuspended in column buffer (CB: 20 mM Tris/HCl, pH 7.5; 200 mM NaCl; 1 mM EDTA; 10 mM β-mercaptoethanol). The cells were lysed by freeze-thaw followed by sonication. The extract was clarified by centrifugation (2 x 15 min at 10,000 g) and filtering through paper filter and applied onto a column packed with amylose resin (New England Biolabs, Ipswich, MA, USA) equilibrated with CB. After wash with CB (30 mL per 2 mL resin), the proteins were eluted with CB containing 10 mM maltose. The eluates were concentrated using AmiconUltra-15 centrifugal filters (Merck Millipore) and stored at -20°C in 25 % glycerol. The protein concentration was determined by the method of Bradford.^98^ The protein expression and purification procedure was performed at least two times for each enzyme with the same results.

#### Enzyme activity assays

Isopentenyl, dimethylallyl, and geranyl diphosphate (IPP, DMAPP, and GPP) were purchased as tri-ammonium salts from Echelon Biosciences (Salt Lake City, UT, USA). The compounds were dissolved in the mixture of methanol:water (7:3) at the concentration of 1 mg/mL and stored at -20°C. The activity assays were carried out using 10–180 μg of protein in a total volume of 100 μL in 35 mM HEPES buffer, pH 7.4, containing 10 mM MgCl_2_ and 5 mM β-mercaptoethanol at 30°C for 30 or 60 min. Regular coupling activity was determined using IPP and DMAPP as substrates, both at 100 μM, while the assays for irregular activity contained 200 μM of only DMAPP. The reaction was stopped by adding 100 μL of chloroform, and the proteins were precipitated by vigorous shaking followed by centrifugation (10 min at 16,000 g). The water phase (injection volume 5 μL) was used for LC-MS analysis performed on an 1260 Infinity HPLC system coupled to a G6120B quadrupole mass spectrometry detector (Agilent Technologies, Santa Clara, CA, USA). The separation was carried out on a Poroshell 120 EC-C18 (3.0 x 50 mm, 2.7 μm) column (Agilent Technologies, Santa Clara, CA, USA) using the method described in Nagel et al., 2012.^99^ The detection of C10 and C15 prenyl diphosphates was performed in negative single ion mode; m/z 313 and 381, respectively. The quantity of formed products was calculated based on peak areas using a calibration line built for GPP.

For analysis of regular terpene alcohols, the reactions were carried out with 70–500 μg of protein in a total volume of 400 μL in 35 mM HEPES buffer, pH 7.4, containing 10 mM MgCl_2_, and substrates (IPP and DMAPP, 1 mM for *trans*IDS5 and *trans*IDS3, 10 mM for *trans*IDS2, *trans*IDS11, and *trans*IDS17) at 28°C overnight. For dephosphorylation of prenyl diphosphates, the reaction mixture was supplemented with 80 μL of 0.5 M glycine-NaOH buffer (pH 10.5) containing 5 mM ZnSO_4_; 80 units of calf intestinal alkaline phosphatase (Promega, Madison, WI, USA) were added and the mixture was incubated at 37°C for 1 h. The alcohols were extracted 3 times with 400 μL of methyl tert-butyl ether (MTBE); the organic phase was evaporated to approximately 150 μL under compressed air flow. GC-MS analysis for detection of C10 products was carried out on a QP2010 Ultra (Shimadzu, Duisburg, Germany). The gas chromatograph (GC) was equipped with a ZB-5MS fused silica capillary column (30 m, 0.25 mm ID, df = 0.25 μm) from Phenomenex (Aschaffenburg, Germany). 1-3 μl sample aliquots (depending on concentrations) were injected by using an AOC-20i autosampler-system (Shimadzu, Duisburg, Germany) into a PTV-split/splitless-injector (Optic 4, ATAS GL, Eindhoven, Netherlands), which operated in splitless-mode. PTV injection-temperature was programmed from initial 50°C to 250°C (heating-rate of 50°C/s) and then an isothermal hold until the end of the GC run. Hydrogen was used as carrier-gas with a constant flow rate of 1.3 mL/min. The GC-temperature profile was 60°C for 2 min, then from 60°C to 120°C at 2°C per min, from 120°C to 230°C at 10°C per min, from 230°C to 320°C at 20°C, with a final temperature hold for 5 min. Electron ionization mass spectra were recorded at 70 eV from m/z 40 to 250. The ion source of the mass spectrometer and the transfer line were kept at 250°C. GC-MS analysis for detection of C15 products was carried out on Cyclosil-B capillary column (30 m x 0.250 mm x 0.25 μm; Agilent Technologies, Santa Clara, CA, USA) with the following temperature gradient: 50°C for 3 min → from 50°C to 120°C at 2°C per min → 120°C to 230°C at 10°C per min. Helium was applied as the carrier gas at a flow rate of 1 mL/min. Injection volume was 1 μL (1:100 split). For identification of irregular products of *trans*IDS5, 1 mg of protein was incubated with 2 mg of DMAPP tri-ammonium salt in 1.6 mL of 35 mM HEPES buffer, pH 7.4, containing 10 mM MgCl2 at 28°C overnight. The products were dephosphorylated and extracted as described above, and analysed by GC-MS using the protocol for C10 regular products. Maconelliol standard was synthesized by the protocol of Dunkelblum et al., 2002^22^ and its structure confirmed by 1H- and 13C-NMR measurements.

#### Candidate expression in *Nicotiana benthamiana*

Genetic constructs for candidate expression in plants were assembled with the Golden Braid (GB) cloning system.^100^ Transcriptional units in alpha-level GB vectors included IDS coding sequences fused with a C-terminal His-tag under control of the 35S CaMV promoter. Each candidate gene was tested in its native form and with the addition of a modified chloroplast transit peptide (cTP) of ribulose bisphosphate carboxylase small subunit from *Nicotiana sylvestris* (GenBank: XP_009794476.1). If a different (e.g. mitochondrial) localization signal was predicted, it was removed before adding the cTP. Transient expression in *N. benthamiana* leaves was carried out as described earlier.^101^ For detection of irregular IDS activity, the plant material (200 mg) was frozen in liquid nitrogen, ground and extracted with 80 % methanol (400 μL) by 30 min incubation in the ice ultrasound bath. Supernatants after two centrifugation steps (10 min at 16,000 g) were analysed using 1260 Infinity HPLC system coupled to G6120B quadrupole mass spectrometry detector (Agilent Technologies, Santa Clara, CA, USA). The separation was carried out on Zorbax Extend-C18 (4.6 x 150 mm, 3.5 μm) column (Agilent Technologies, Santa Clara, CA, USA) with mobile phase consisting of 5 mM ammonium bicarbonate in water as solvent A and acetonitrile as solvent B applying the following gradient (% B): starting at 0, 0 to 8 within 2 min, holding 8 for 15 min, 8 to 64 within 3 min, 64 to 100 within 2 min, holding 100 for 4 min, 100 to 0 within 1 min, re-equilibrating at 0 for 12 min. The detection of C10 and C15 prenyl diphosphates was performed in negative single ion mode; m/z 313 and 381, respectively. For detection of volatile compounds, samples from agroinfiltrated leaves were collected 5 dpi (days post infiltration), snap-frozen in liquid nitrogen and analysed by GC-MS as described previously.^24^

#### Computational model and site-directed mutagenesis of *trans*IDS5

The 3-D model of *trans*IDS5 was built on the SWISS-MODEL homology-modelling server^89^ using the crystal structure of *Saccharomyces cerevisiae* geranylgeranyl pyrophosphate synthase in complex with magnesium and IPP^70^ (PDB: 2E8U, https://doi.org/10.2210/pdb2E8U/pdb) as a template. For structure visualisation and analysis, UCSF Chimera v.1.14 was applied.^102^ The location of prenyl substrates was derived by superposition of the *trans*IDS5 model and the template structure. Mutations were introduced using the Q5-SDM kit (New England Biolabs) with primers listed in Table S8.

### Quantification and statistical analysis

Short read mapping counts for all three datasets were used for differential transcript expression analysis based on R packages edgeR and limma.^74^^,^^75^^,^^94^

## Supplemental information

Document S1. Figures S1–S27 and Tables S1, S2, and S5–S8

Table S3. Transcriptome annotation and differential expression data

Table S4. Selected IDS sequences, related to Table 1 List of putative IDS Pcitri.v1 gene models (a) and transcripts (b), and final IDS sequences (c) after collapsing. Tables in (a) and (b) include the InterPro (IPR) Annotation with the IPR family ID and name, candidate ID, start and end positions of the identified domain and E-value of the IPR Annotation. Table (b) also includes information on whether the sequence originates from long-read or short-read transcriptome. Gray shaded rows in (a) mark candidates determined by the MAST motif-search. Table (c) includes a list of all selected cis- and trans-IDS sequences with information on source sequences from the Pcitri.v1 genome or the transcriptome with their expression values, including log2 fold-changes contrasting virgin to mated females, as well as normalised and log-transformed counts per million (CPM). It also includes information whether the candidate sequence was successfully amplified or synthesized (see also Table 1), the amino acid sequence of the amplified/synthesized sequences and the putative species of origin, deduced from pyhlogenetic analyses and blast results.

## Footnotes

Supplemental information can be found online at https://doi.org/10.1016/j.isci.2024.109441.

## References

[1] Tillman J.A., Seybold S.J., Jurenka R.A., Blomquist G.J. (1999). Insect pheromones - an overview of biosynthesis and endocrine regulation. Insect Biochem. Mol. Biol..

[2] Beran F., Köllner T.G., Gershenzon J., Tholl D. (2019). Chemical convergence between plants and insects: biosynthetic origins and functions of common secondary metabolites. New Phytol..

[3] Zou Y., Millar J.G. (2015). Chemistry of the pheromones of mealybug and scale insects. Nat. Prod. Rep..

[4] Franco J.C., Cocco A., Lucchi A., Mendel Z., Suma P., Vacas S., Mansour R., Navarro-Llopis V. (2022). Scientific and technological developments in mating disruption of scale insects. Entomol. Gen..

[5] Vacas S., Alfaro C., Navarro-Llopis V., Primo J. (2010). Mating disruption of California red scale, *Aonidiella aurantii* Maskell (Homoptera: Diaspididae), using biodegradable mesoporous pheromone dispensers. Pest Manag. Sci..

[6] Zou Y., Chinta S.P., Millar J.G., Beck J.J., Coats J.R., Duke S.O., Koivunen M.E. (2013). Pest Management with Natural Products.

[7] Lucchi A., Suma P., Ladurner E., Iodice A., Savino F., Ricciardi R., Cosci F., Marchesini E., Conte G., Benelli G. (2019). Managing the vine mealybug, *Planococcus ficus*, through pheromone-mediated mating disruption. Environ. Sci. Pollut. Res. Int..

[8] Daane K.M., Cooper M.L., Mercer N.H., Hogg B.N., Yokota G.Y., Haviland D.R., Welter S.C., Cave F.E., Sial A.A., Boyd E.A. (2021). Pheromone Deployment Strategies for Mating Disruption of a Vineyard Mealybug. J. Econ. Entomol..

[9] Nagel R., Schmidt A., Peters R.J. (2019). Isoprenyl diphosphate synthases: the chain length determining step in terpene biosynthesis. Planta.

[10] Kobayashi M., Kuzuyama T. (2019). Structural and Mechanistic Insight into Terpene Synthases that Catalyze the Irregular Non-Head-to-Tail Coupling of Prenyl Substrates. Chembiochem.

[11] Rivera S.B., Swedlund B.D., King G.J., Bell R.N., Hussey C.E., Shattuck-Eidens D.M., Wrobel W.M., Peiser G.D., Poulter C.D. (2001). Chrysanthemyl diphosphate synthase: isolation of the gene and characterization of the recombinant non-head-to-tail monoterpene synthase from *Chrysanthemum cinerariaefolium*. Proc. Natl. Acad. Sci. USA.

[12] Hemmerlin A., Rivera S.B., Erickson H.K., Poulter C.D. (2003). Enzymes encoded by the farnesyl diphosphate synthase gene family in the Big Sagebrush *Artemisia tridentata* ssp. *spiciformis*. J. Biol. Chem..

[13] Demissie Z.A., Erland L.A.E., Rheault M.R., Mahmoud S.S. (2013). The biosynthetic origin of irregular monoterpenes in lavandula: Isolation and biochemical characterization of a novel *cis*-prenyl diphosphate synthase gene, lavandulyl diphosphate synthase. J. Biol. Chem..

[14] Chan Y.-T., Ko T.-P., Yao S.-H., Chen Y.-W., Lee C.-C., Wang A.H.-J. (2017). Crystal Structure and Potential Head-to-Middle Condensation Function of a *Z,Z*-Farnesyl Diphosphate Synthase. ACS Omega.

[15] Ozaki T., Zhao P., Shinada T., Nishiyama M., Kuzuyama T. (2014). Cyclolavandulyl Skeleton Biosynthesis via Both Condensation and Cyclization Catalyzed by an Unprecedented Member of the *cis*-Isoprenyl Diphosphate Synthase Superfamily. J. Am. Chem. Soc..

[16] Teufel R., Kaysser L., Villaume M.T., Diethelm S., Carbullido M.K., Baran P.S., Moore B.S. (2014). One-pot enzymatic synthesis of merochlorin A and B. Angew. Chem. Int. Ed. Engl..

[17] Ogawa T., Emi K.I., Koga K., Yoshimura T., Hemmi H. (2016). A *cis*-prenyltransferase from *Methanosarcina acetivorans* catalyzes both head-to-tail and nonhead-to-tail prenyl condensation. FEBS J..

[18] Emi K.I., Sompiyachoke K., Okada M., Hemmi H. (2019). A heteromeric *cis*-prenyltransferase is responsible for the biosynthesis of glycosyl carrier lipids in *Methanosarcina mazei*. Biochem. Biophys. Res. Commun..

[19] Watson G. (2023). CABI Compendum, *Planococcus citri* (citrus mealybug). https://www.cabidigitallibrary.org/doi/10.1079/cabicompendium.45082.

[20] Bierl-Leonhardt B.A., Moreno D.S., Schwarz M., Fargerlund J., Plimmer J.R. (1981). Isolation, identification and synthesis of the sex pheromone of the citrus mealybug, *Planococcus citri* (Risso). Tetrahedron Lett..

[21] Wolk J.L., Goldschmidt Z., Dunkelblum E. (1986). A short stereoselective synthesis of (+)-cis-planococcyl acetate, sex pheromone of the citrus mealybug *Planococcus citri* (Risso). Synthesis.

[22] Dunkelblum E., Zada A., Gross S., Fraistat P., Mendel Z. (2002). Sex pheromone and analogs of the citrus mealybug, *Planococcus citri*: synthesis and biological activity. IOBC-WPRS Bullertins.

[23] Passaro L.C., Webster F.X. (2004). Synthesis of the Female Sex Pheromone of the citrus mealybug, *Planococcus citri*. J. Agric. Food Chem..

[24] Mateos-Fernández R., Moreno-Giménez E., Gianoglio S., Quijano-Rubio A., Gavaldá-García J., Estellés L., Rubert A., Rambla J.L., Vazquez-Vilar M., Huet E. (2021). Production of Volatile Moth Sex Pheromones in Transgenic *Nicotiana benthamiana* Plants. Biodes. Res..

[25] Petkevicius K., Wenning L., Kildegaard K.R., Sinkwitz C., Smedegaard R., Holkenbrink C., Borodina I. (2022). Biosynthesis of insect sex pheromone precursors via engineered β-oxidation in yeast. FEMS Yeast Res..

[26] Laetsch D. (2017). *Planococcus citri* Pcitri.v1. https://ensembl.mealybug.org/Planococcus_citri_pcitriv1/Info/Index.

[27] Levi-Zada A., Fefer D., David M., Eliyahu M., Franco J.C., Protasov A., Dunkelblum E., Mendel Z. (2014). Diel periodicity of pheromone release by females of *Planococcus citri* and *Planococcus ficus* and the temporal flight activity of their conspecific males. Naturwissenschaften.

[28] Manni M., Berkeley M.R., Seppey M., Zdobnov E.M. (2021). BUSCO: Assessing Genomic Data Quality and Beyond. Curr. Protoc..

[29] Beran F., Petschenka G. (2022). Sequestration of Plant Defense Compounds by Insects: From Mechanisms to Insect-Plant Coevolution. Annu. Rev. Entomol..

[30] Jančič S., Nguyen H.D.T., Frisvad J.C., Zalar P., Schroers H.J., Seifert K.A., Gunde-Cimerman N. (2015). A Taxonomic Revision of the *Wallemia sebi* Species Complex. PLoS One.

[31] Zhang H., Li Z.-X. (2013). *In vitro* and *in vivo* characterization of a novel insect decaprenyl diphosphate synthase: A two-major step catalytic mechanism is proposed. Biochem. Biophys. Res. Commun..

[32] Song X., Li Z.-X. (2022). Functional characterization of two different decaprenyl diphosphate synthases in the vetch aphid *Megoura viciae*. Arch. Insect Biochem. Physiol..

[33] Song X., Qin Y.G., Zhang Y.H., Zhou Y.B., Li Z.X. (2023). Farnesyl/geranylgeranyl diphosphate synthases regulate the biosynthesis of alarm pheromone in a unique manner in the vetch aphid *Megoura viciae*. Insect Mol. Biol..

[34] Cheng Y.J., Li Z.X. (2019). Both farnesyl diphosphate synthase genes are involved in the production of alarm pheromone in the green peach aphid *Myzus persicae*. Arch. Insect Biochem. Physiol..

[35] Ding B.Y., Niu J., Shang F., Yang L., Chang T.Y., Wang J.J. (2019). Characterization of the Geranylgeranyl Diphosphate Synthase Gene in *Acyrthosiphon pisum* (Hemiptera: Aphididae) and Its Association With Carotenoid Biosynthesis. Front. Physiol..

[36] Rebholz Z., Lancaster J., Larose H., Khrimian A., Luck K., Sparks M.E., Gendreau K.L., Shewade L., Köllner T.G., Weber D.C. (2023). Ancient origin and conserved gene function in terpene pheromone and defense evolution of stink bugs and hemipteran insects. Insect Biochem. Mol. Biol..

[37] Lancaster J., Khrimian A., Young S., Lehner B., Luck K., Wallingford A., Ghosh S.K.B., Zerbe P., Muchlinski A., Marek P.E. (2018). *De novo* formation of an aggregation pheromone precursor by an isoprenyl diphosphate synthase-related terpene synthase in the harlequin bug. Proc. Natl. Acad. Sci. USA.

[38] Hemmerlin A., Harwood J.L., Bach T.J. (2012). A raison d’être for two distinct pathways in the early steps of plant isoprenoid biosynthesis?. Prog. Lipid Res..

[39] Liu P.-L., Wan J.-N., Guo Y.-P., Ge S., Rao G.-Y. (2012). Adaptive evolution of the chrysanthemyl diphosphate synthase gene involved in irregular monoterpene metabolism. BMC Evol. Biol..

[40] Gao Y., Honzatko R.B., Peters R.J. (2012). Terpenoid synthase structures: A so far incomplete view of complex catalysis. Nat. Prod. Rep..

[41] Burke C.C., Wildung M.R., Croteau R. (1999). Geranyl diphosphate synthase: Cloning, expression, and characterization of this prenyltransferase as a heterodimer. Proc. Natl. Acad. Sci. USA.

[42] Tholl D., Kish C.M., Orlova I., Sherman D., Gershenzon J., Pichersky E., Dudareva N. (2004). Formation of monoterpenes in *Antirrhinum majus* and *Clarkia breweri* flowers involves heterodimeric geranyl diphosphate synthases. Plant Cell.

[43] Qu Y., Chakrabarty R., Tran H.T., Kwon E.-J.G., Kwon M., Nguyen T.-D., Ro D.-K. (2015). A lettuce (*Lactuca sativa*) homolog of human Nogo-B receptor interacts with *cis*-prenyltransferase and is necessary for natural rubber biosynthesis. J. Biol. Chem..

[44] Yin J.-L., Wong W.-S., Jang I.-C., Chua N.-H. (2017). Co-expression of peppermint geranyl diphosphate synthase small subunit enhances monoterpene production in transgenic tobacco plants. New Phytol..

[45] Zhou F., Wang C.-Y., Gutensohn M., Jiang L., Zhang P., Zhang D., Dudareva N., Lu S. (2017). A recruiting protein of geranylgeranyl diphosphate synthase controls metabolic flux toward chlorophyll biosynthesis in rice. Proc. Natl. Acad. Sci. USA.

[46] Dong C., Zhang M., Song S., Wei F., Qin L., Fan P., Shi Y., Wang X., Wang R. (2023). A Small Subunit of Geranylgeranyl Diphosphate Synthase Functions as an Active Regulator of Carotenoid Synthesis in *Nicotiana tabacum*. Int. J. Mol. Sci..

[47] Schneider L., Rebetez M., Rasmann S. (2022). The effect of climate change on invasive crop pests across biomes. Curr. Opin. Insect Sci..

[48] Mateos Fernández R., Petek M., Gerasymenko I., Juteršek M., Baebler Š., Kallam K., Moreno Giménez E., Gondolf J., Nordmann A., Gruden K. (2022). Insect pest management in the age of synthetic biology. Plant Biotechnol. J..

[49] Li F., Zhao X., Li M., He K., Huang C., Zhou Y., Li Z., Walters J.R. (2019). Insect genomes: progress and challenges. Insect Mol. Biol..

[50] Hotaling S., Sproul J.S., Heckenhauer J., Powell A., Larracuente A.M., Pauls S.U., Kelley J.L., Frandsen P.B. (2021). Long Reads Are Revolutionizing 20 Years of Insect Genome Sequencing. Genome Biol. Evol..

[51] Wallrapp F.H., Pan J.J., Ramamoorthy G., Almonacid D.E., Hillerich B.S., Seidel R., Patskovsky Y., Babbitt P.C., Almo S.C., Jacobson M.P., Poulter C.D. (2013). Prediction of function for the polyprenyl transferase subgroup in the isoprenoid synthase superfamily. Proc. Natl. Acad. Sci. USA.

[52] Zhang A., Amalin D., Shirali S., Serrano M.S., Franqui R.A., Oliver J.E., Klun J.A., Aldrich J.R., Meyerdirk D.E., Lapointe S.L. (2004). Sex pheromone of the pink hibiscus mealybug, *Maconellicoccus hirsutus*, contains an unusual cyclobutanoid monoterpene. Proc. Natl. Acad. Sci. USA.

[53] Tholl D., Rebholz Z., Morozov A.V., O’Maille P.E. (2023). Terpene synthases and pathways in animals: enzymology and structural evolution in the biosynthesis of volatile infochemicals. Nat. Prod. Rep..

[54] Karunanithi P.S., Zerbe P. (2019). Terpene Synthases as Metabolic Gatekeepers in the Evolution of Plant Terpenoid Chemical Diversity. Front. Plant Sci..

[55] Gilg A.B., Tittiger C., Blomquist G.J. (2009). Unique animal prenyltransferase with monoterpene synthase activity. Naturwissenschaften.

[56] Beran F., Rahfeld P., Luck K., Nagel R., Vogel H., Wielsch N., Irmisch S., Ramasamy S., Gershenzon J., Heckel D.G., Köllner T.G. (2016). Novel family of terpene synthases evolved from *trans*-isoprenyl diphosphate synthases in a flea beetle. Proc. Natl. Acad. Sci. USA.

[57] Darragh K., Orteu A., Black D., Byers K.J.R.P., Szczerbowski D., Warren I.A., Rastas P., Pinharanda A., Davey J.W., Fernanda Garza S. (2021). A novel terpene synthase controls differences in anti-aphrodisiac pheromone production between closely related *Heliconius butterflies*. PLoS Biol..

[58] Nishida R., Kim C.-S., Fukami H., Irik R. (1991). Ideamine N-Oxides: Pyrrolizidine Alkaloids Sequestered by the Danaine Butterfly, *Idea leuconoe*. Agric. Biol. Chem..

[59] Husnik F., Nikoh N., Koga R., Ross L., Duncan R.P., Fujie M., Tanaka M., Satoh N., Bachtrog D., Wilson A.C.C. (2013). Horizontal Gene Transfer from Diverse Bacteria to an Insect Genome Enables a Tripartite Nested Mealybug Symbiosis. Cell.

[60] McCutcheon J.P., von Dohlen C.D. (2011). An Interdependent Metabolic Patchwork in the Nested Symbiosis of Mealybugs. Curr. Biol..

[61] López-Madrigal S., Latorre A., Porcar M., Moya A., Gil R. (2013). Mealybugs nested endosymbiosis: going into the ‘matryoshka’ system in *Planococcus citri* in depth. BMC Microbiol..

[62] Tabata J. (2022). Genetic Basis Underlying Structural Shift of Monoterpenoid Pheromones in Mealybugs. J. Chem. Ecol..

[63] Rudolf J.D., Chang C.-Y. (2020). Terpene synthases in disguise: enzymology, structure, and opportunities of non-canonical terpene synthases. Nat. Prod. Rep..

[64] Köllner T.G., David A., Luck K., Beran F., Kunert G., Zhou J.-J., Caputi L., O’Connor S.E. (2022). Biosynthesis of iridoid sex pheromones in aphids. Proc. Natl. Acad. Sci. USA.

[65] Vogel H., Heidel A.J., Heckel D.G., Groot A.T. (2010). Transcriptome analysis of the sex pheromone gland of the noctuid moth *Heliothis virescens*. BMC Genom..

[66] Gu S.-H., Wu K.-M., Guo Y.-Y., Pickett J.A., Field L.M., Zhou J.-J., Zhang Y.-J. (2013). Identification of genes expressed in the sex pheromone gland of the black cutworm *Agrotis ipsilon* with putative roles in sex pheromone biosynthesis and transport. BMC Genom..

[67] Nuo S.-M., Yang A.-J., Li G.-C., Xiao H.-Y., Liu N.-Y. (2021). Transcriptome analysis identifies candidate genes in the biosynthetic pathway of sex pheromones from a zygaenid moth , *Achelura yunnanensis* ( Lepidoptera : Zygaenidae ). PeerJ.

[68] Yao S., Zhou S., Li X., Liu X., Zhao W., Wei J., Du M., An S. (2021). Transcriptome Analysis of *Ostrinia furnacalis* Female Pheromone Gland: Esters Biosynthesis and Requirement for Mating Success. Front. Endocrinol..

[69] Gerasymenko I., Sheludko Y.V., Navarro Fuertes I., Schmidts V., Steinel L., Haumann E., Warzecha H. (2022). Engineering of a Plant Isoprenyl Diphosphate Synthase for Development of Irregular Coupling Activity. Chembiochem.

[70] Guo R.T., Cao R., Liang P.H., Ko T.P., Chang T.H., Hudock M.P., Jeng W.Y., Chen C.K.M., Zhang Y., Song Y. (2007). Bisphosphonates target multiple sites in both *cis*- and *trans*- prenyltransferases. Proc. Natl. Acad. Sci. USA.

[71] Husnik F. (2017). *Planococcus ficus* Pficus.v0. https://ensembl.mealybug.org/Planococcus_ficus_pficusv0/Info/Index.

[72] Laetsch D. (2017). *Pseudococcus longispinus* v1. https://ensembl.mealybug.org/Pseudococcus_longispinus_v1/Info/Index.

[73] Kohli S., Gulati P., Narang A., Maini J., Shamsudheen K.V., Pandey R., Scaria V., Sivasubbu S., Brahmachari V. (2021). Genome and transcriptome analysis of the mealybug *Maconellicoccus hirsutus*: Correlation with its unique phenotypes. Genomics.

[74] Robinson M.D., McCarthy D.J., Smyth G.K. (2010). edgeR: a Bioconductor package for differential expression analysis of digital gene expression data. Bioinformatics.

[75] Ritchie M.E., Phipson B., Wu D., Hu Y., Law C.W., Shi W., Smyth G.K. (2015). *limma* powers differential expression analyses for RNA-sequencing and micorarray studies. Nucleic Acids Res..

[76] Li H. (2018). Minimap2: Pairwise alignment for nucleotide sequences. Bioinformatics.

[77] Tseng E. (2022). cDNA_Cupcake. https://github.com/Magdoll/cDNA_Cupcake.

[78] Bushnell B. (2022). BBMap. https://sourceforge.net/projects/bbmap/.

[79] Bushmanova E., Antipov D., Lapidus A., Prjibelski A.D. (2019). RnaSPAdes: A *de novo* transcriptome assembler and its application to RNA-Seq data. GigaScience.

[80] Bushmanova E., Antipov D., Lapidus A., Suvorov V., Prjibelski A.D. (2016). RnaQUAST: A quality assessment tool for *de novo* transcriptome assemblies. Bioinformatics.

[81] Skelly T. (2015). MatchAnnot. https://github.com/TomSkelly/MatchAnnot.

[82] Haas B. (2023). TransDecoder. https://github.com/TransDecoder/TransDecoder.

[83] Jones P., Binns D., Chang H.-Y., Fraser M., Li W., McAnulla C., McWilliam H., Maslen J., Mitchell A., Nuka G. (2014). InterProScan 5: Genome-scale protein function classification. Bioinformatics.

[84] Mirdita M., Steinegger M., Breitwieser F., Söding J., Levy Karin E. (2021). Fast and sensitive taxonomic assignment to metagenomic contigs. Bioinformatics.

[85] Breitwieser F.P., Salzberg S.L. (2020). Pavian: interactive analysis of metagenomics data for microbiome studies and pathogen identification. Bioinformatics.

[86] Bailey T.L., Johnson J., Grant C.E., Noble W.S. (2015). The MEME Suite. Nucleic Acids Res..

[87] Altschul S.F., Gish W., Miller W., Myers E.W., Lipman D.J. (1990). Basic Local Alignment Search Tool. J. Mol. Biol..

[88] Fu L., Niu B., Zhu Z., Wu S., Li W. (2012). CD-HIT: Accelerated for clustering the next-generation sequencing data. Bioinformatics.

[89] Waterhouse A., Bertoni M., Bienert S., Studer G., Tauriello G., Gumienny R., Heer F.T., De Beer T.A.P., Rempfer C., Bordoli L. (2018). SWISS-MODEL: Homology modelling of protein structures and complexes. Nucleic Acids Res..

[90] Kumar S., Stecher G., Li M., Knyaz C., Tamura K. (2018). MEGA X: Molecular evolutionary genetics analysis across computing platforms. Mol. Biol. Evol..

[91] Dobin A., Davis C.A., Schlesinger F., Drenkow J., Zaleski C., Jha S., Batut P., Chaisson M., Gingeras T.R. (2013). STAR: Ultrafast universal RNA-seq aligner. Bioinformatics.

[92] Vacas S., Navarro I., Seris E., Ramos C., Hernández E., Navarro-Llopis V., Primo J. (2017). Identification of the male-produced aggregation pheromone of the four-spotted coconut weevil, *Diocalandra frumenti*. J. Agric. Food Chem..

[93] Kukovinets O.S., Zvereva T.I., Kasradze V.G., Galin F.Z., Frolova L.L., Kuchin A.V., Spirikhin L.V., Abdullin M.I. (2006). Novel synthesis of *Planococcus citri* pheromone. Chem. Nat. Compd..

[94] Law C.W., Alhamdoosh M., Su S., Dong X., Tian L., Smyth G.K., Ritchie M.E. (2018). RNA-seq analysis is easy as 1-2-3 with limma, Glimma and edgeR. F1000Research.

[95] Nikolenko S.I., Korobeynikov A.I., Alekseyev M.A. (2013). BayesHammer : Bayesian clustering for error correction in single-cell sequencing. BMC Genom..

[96] Edgar R.C. (2004). MUSCLE: Multiple sequence alignment with high accuracy and high throughput. Nucleic Acids Res..

[97] Le S.Q., Gascuel O. (2008). An Improved General Amino Acid Replacement Matrix. Mol. Biol. Evol..

[98] Bradford M.M. (1976). A rapid and sensitive method for the quantitation of microgram quantitites of protein utilizing the principle of protein-dye binding. Anal. Biochem..

[99] Nagel R., Gershenzon J., Schmidt A. (2012). Nonradioactive assay for detecting isoprenyl diphosphate synthase activity in crude plant extracts using liquid chromatography coupled with tandem mass spectrometry. Anal. Biochem..

[100] Sarrion-Perdigones A., Falconi E.E., Zandalinas S.I., Juárez P., Fernández-del-Carmen A., Granell A., Orzaez D. (2011). GoldenBraid: An iterative cloning system for standardized assembly of reusable genetic modules. PLoS One.

[101] Gerasymenko I., Sheludko Y., Fräbel S., Staniek A., Warzecha H. (2019). Combinatorial biosynthesis of small molecules in plants: Engineering strategies and tools. Methods Enzymol..

[102] Pettersen E.F., Goddard T.D., Huang C.C., Couch G.S., Greenblatt D.M., Meng E.C., Ferrin T.E. (2004). UCSF Chimera - A visualization system for exploratory research and analysis. J. Comput. Chem..

