# Supplementary material for: Transcriptome-informed identification and characterization of Planococcus citri cis- and trans-isoprenyl diphosphate synthase genes
Source: iScience. 2024 Mar 6;27(4):109441. doi: 10.1016/j.isci.2024.109441 (PMC10960109; doi:10.1016/j.isci.2024.109441)
Supplement: Document S1. Figures S1–S27 and Tables S1, S2, and S5–S8 [file mmc1.pdf]

iScience, Volume *27*

Supplemental information

Transcriptome-informed identification and characterization of *Planococcus citri cis*- and *trans*-isoprenyl diphosphate synthase genes

Mojca Juteršek, Iryna M. Gerasymenko, Marko Petek, Elisabeth Haumann, Sandra Vacas, Kalyani Kallam, Silvia Gianoglio, Vicente Navarro-Llopis, Michael Heethoff, Ismael Navarro Fuertes, Nicola Patron, Diego Orzáez, Kristina Gruden, Heribert Warzecha, and Špela Baebler

Table S1: Quality assessment of short- and long-read transcriptome data, related to STAR Methods. (a) Combined rnaQUAST outputs for the consolidated transcriptome (Figure S1e) and each of the transcriptome datasets separately (short-read *de novo* assembly v1 – Figure S1a, short-read *de novo* assembly v2 – Figure S1b, and long-read Iso-Seq transcriptome – Figure S1d). (b) Metrics determined for the *Pcitri*.v1 genome assembly, to which the transcriptome datasets were mapped.

a) Transcriptome metrics

	Consolidated transcriptome	Long-read transcriptome	*de novo* v1 transcriptome	*de novo* v2 transcriptome
Total number of assembled transcripts	440,881	72,445	317,306	134,464
Transcripts > 500	234,111	71,487	75,790	122,374
Transcripts >1000	168,571	63,478	44,334	81,127
Average length of assembled transcripts	1,356.41	2,358.59	633.64	2,180.98
Longest transcript	211,937	12,275	124,003	211,973
Total length	598,016,656	170,867,687	201,059,045	293,262,838
Transcript N50	2,908	2,800	1,515	3,495
**Transcriptome alignment to *Pcitri*.v1 genome**				
Aligned	363,614	68,843	248,614	111,385
Uniquely aligned	315,563	55,010	229,305	91,910
Multipy aligned	6,828	35	6,962	651
Misassembly candidates reported by GMAP	41,058	13,759	12,297	19,038
Misassembly candidates reported by BLASTN	88,030	24,581	29,664	42,074
Misassemblies	22,983	8,801	5,270	11,052
Unaligned	77,267	3,602	68,692	23,079
Average aligned fraction	0.97	0.96	0.98	0.95
Average alignment length	1,196.85	2,120.38	596.42	1,952.25
Average blocks per alignment	5.93	12.44	2.95	8.64
Average block length	201.73	170.52	210.92	225.95
Average mismatches per transcript	8.31	6.17	6.80	9.71
NA50	2,546	2,550	1,310	3,002

**Assembly completeness (sensitivity)**				
Database coverage	0.67	0.43	0.54	0.56
Duplication ratio	5.02	3.63	1.65	2.47
Average number of transcripts mapped to one isoform	5.20	3.64	2.52	2.68
50%-assembled genes	21,775	12,919	16,445	17,640
95%-assembled genes	16,687	10,806	10,887	13,392
50%-covered genes	22,664	12,988	18,039	19,015
95%-covered genes	17,929	11,082	12,718	14,218
50%-assembled isoforms	22,293	13,280	16,625	18,004
95%-assembled isoforms	16,976	11,005	10,949	13,558
50%-covered isoforms	23,203	13,352	18,244	18,404
95%-covered isoforms	18,258	11,298	12,789	14,403
50%-assembled exons	118,790	85,346	98,980	102,164
95%-assembled exons	113,418	83,603	91,952	98,760
Mean isoform assembly	0.81	0.96	0.69	0.84
Mean isoform coverage	0.84	0.93	0.74	0.86
Mean exon coverage	0.96	0.99	0.93	0.97
Average percentage of isoform 50%-covered exons	0.83	0.93	0.74	0.86
Average percentage of isoform 95%-covered exons	0.74	0.89	0.61	0.79
**Assembly specificity**				
Unannotated	178,976	4,132	174,060	36,232
50%-matched	76,324	36,763	33,960	23,761
95%-matched	8,632	2,203	8,330	1,196
Mean fraction of transcript matched	0.23	0.59	0.14	0.27
Mean fraction of block matched	0.46	0.56	0.46	0.40
50%-matched blocks	0.46	0.56	0.47	0.40
95%-matched blocks	0.39	0.50	0.37	0.33
Matched length	157,253,815	72,465,708	41,811,301	64,516,908
Unmatched length	265,414,648	49,779,570	111,379,414	130,860,407

b) Genome metrics

Pcitri*.v1 *genome	
Genes	40,620
Isoforms	42,260
Average length of all isoforms	1,104.78
Total length of all isoforms	46,687,951
Exons	180,975
Average exon length	257.98
Average number of exons per isoform	4.28
Maximal number of exons per isoform	117
Introns	138,715
Average intron length	853.25

Table S2: Percent of short reads mapping to transcriptome sequences, related to STAR Methods. Illumina short-read samples used for *P. citri* short-read transcriptome assembly with SRA or GEO accession numbers and percentages of reads mapping to all three generated transcriptome datasets, counting uniquely and multimapped reads. The first seven samples were provided by Edinburgh University (E) and include samples from *P. citri* males (M) and females (F). Eight samples were sequenced in this study (S) and include samples from *P. citri* mated and virgin females (MF and VF, respectively). Only reads from this study were mapped back to v1 of short-read transcriptome, as it was assembled from those reads only.

Sample	*Sample accession*	*Mapped to short-read v1*	*Mapped to short-read v2*	*Mapped to long-read*	*Average per sample*
EALL1	/	/	93.26%	86.49%	**89.87%**
EF1	SRR11260463	/	95.12%	92.38%	**93.75%**
EF2	SRR11260462	/	82.79%	79.99%	**81.39%**
EF3	SRR11260471	/	92.75%	89.81%	**91.28%**
EM1	SRR11260470	/	96.12%	82.07%	**89.09%**
EM2	SRR11260469	/	85.29%	73.62%	**79.45%**
EM3	SRR11260468	/	85.75%	72.95%	**79.35%**
SMF08	GSM5425814	74.95%	78.53%	62.37%	**71.95%**
SMF09	GSM5425815	77.28%	78.64%	64.07%	**73.33%**
SMF10	GSM5425816	77.88%	81.10%	70.48%	**76.49%**
SMF11	GSM5425817	79.91%	80.31%	68.04%	**76.08%**
SVF02	GSM5425810	78.15%	80.84%	68.72%	**75.90%**
SVF03	GSM5425811	80.89%	82.10%	67.65%	**76.88%**
SVF05	GSM5425812	84.30%	86.23%	75.74%	**82.09%**
SVF06	GSM5425813	81.00%	82.71%	73.67%	**79.13%**
Average per transcriptome set		**79.29%**	**85.44%**	**75.20%**	

Table S5: IDS activity measurements of *P. citri* candidates, related to Figures 3a and 4a. (a) Raw data for measurements of IDS enzymatic activity for candidate IDSs producing C10 or C15 prenyl diphosphates, source data for Figure 3a, and (b) measurements of *trans*IDS5 mutants, source data for Figure 4a. Three measurements and their average are given for each protein and for each chain length. Values are given in mmol s^{-1} g^{-1}.

a)

	**C10**			**C15**			**C10 average**	**C15 average**
*trans*IDS5	133.71	137.93	139.84	169.16	201.27	209.93	137.16	193.46
*trans*IDS3	22.74	24.00	21.96	2.57	2.58	2.40	22.90	2.51
*trans*IDS11	0.66	0.60	0.55	0.27	0.25	0.23	0.60	0.25
*trans*IDS2	0.60	0.60	0.56	0.32	0.29	0.24	0.59	0.28
*trans*IDS17	0.09	0.09	0.10	0.04	0.03	0.04	0.09	0.04

b)

	**C10**			**C15**			**C10 average**	**C15 average**
*trans*IDS5 -wt	133.71	137.93	139.84	169.16	201.27	209.93	137.16	193.46
D166N	18.20	16.81	21.43	22.79	24.57	26.63	18.81	24.66
D308N	123.54	117.62	115.92	24.18	24.27	22.52	119.03	23.66
D309N	0.00	0.00	0.00	0.00	0.00	0.00	0.00	0.00
D312N	129.20	119.87	125.87	34.90	31.18	36.47	124.98	34.18
K120A	124.45	129.84	132.18	13.41	15.55	14.58	128.82	14.52
K120E	44.60	45.47	43.62	0.00	0.00	0.00	44.56	0.00
K120Q	120.59	118.66	120.46	11.94	11.95	12.04	119.90	11.98

Table S6: Combined activity assays performed for detection of irregular IDS and terpene synthase activities, related to STAR Methods. All cloned IDS candidates were tested in pairwise combinations among each other (top part of the table) as well as in combination with other possible interactors: *P.citri* putative IDS regulatory subunits (*trans*IDS4, *cis*IDS9), g23689.tl, a putative *P. citri* pheromone binding protein (PBP); g36424.tl, *P.citri* isopentenyl diphosphate isomerase (IDI). For detection of terpene synthase activities, IDS-like candidates from *P. citri* were combined with IDS proteins reported to fulfil irregular coupling: chrysanthemyl diphosphate synthase from *Tanacetum cinerariifolium* (*Tc*CPPS) (Rivera et al. 2001[11]), lavandulyl (*Li*LPPS) and cyclolavandulyl (*Ssp*CLPPS) diphosphate synthases from *Lavandula × intermedia* and *Streptomyces* sp. CL190, respectively (Demissie et al. 2013[13]; Ozaki et al. 2014[15]), and a mutant of neryl diphosphate synthase from *Solanum lycopersicum* (*Sl*NPPS-N88H) with increased irregular activity producing *trans*-planococcyl diphosphate (Gerasymenko et al. 2022[66]). Results of individual activities of *P. citri* IDS candidates are given in Table 1, TableS5, Figure 3a, and Figure 4a.

	*trans*IDS10	*trans*IDS3	*trans*IDS12	*trans*IDS5	*trans*IDS11	*trans*IDS2	*trans*IDS16	*trans*IDS17	*cis*IDS1	*cis*IDS8	
*trans*IDS10		X	X	X	X	X	X	X	X	X	Combinations between *P. citri* IDS candidates
*trans*IDS3			X	X	X	X	X	X	X	X	
*trans*IDS12				X	X	X	X	X	X	X	
*trans*IDS5					X	X	X	X	X	X	
*trans*IDS11						X	X	X	X	X	
*trans*IDS2							X	X	X	X	
*trans*IDS16								X	X	X	
*trans*IDS17									X	X	
*cis*IDS1										X	
*cis*IDS8											
*trans*IDS4	X	X	X	X	X	X	X	X	X	X	Combinations with regulatory subunits
*cis*IDS9	X	X	X	X	X	X	X	X	X	X	
g23689.t1 (PBP)	X	X	X	X	X	X	X	X	X	X	Combinations with accessory proteins
g36424.t1 (IDI)	X	X	X	X	X	X	X	X	X	X	
*Tc*CPPS	X	X	X	X	X	X	X	X	X	X	Combinations with IDSs with irregular activity
*Li*LPPS	X	X	X	X	X	X	X	X	X	X	
*Ssp*CLPPS	X	X	X	X	X	X	X	X	X	X	
*Sl*NPPS-N88H	X	X	X	X	X	X	X	X	X	X	

Table S7: Input and output of the MEME motif search for *P. citri* sequences containing *trans*-IDS motifs, related to STAR Methods. Input sequences of FPPS-coding genes from different organisms used for MEME motif search (a) and output of MAST algorithm (b) with sequences from *Pcitri*.v1 genome, which contain the motifs detected by MEME. For the input sequences, organism names and Uniprot accession numbers are given, and for the target sequences, *Pcitri*.v1 gene model IDs and their E-values are given.

a)

Organism	UniProt_acc
Acromyrmex echinatior	F4WXE7
Anthonomus grandis	Q56CY8
Arabidopsis thaliana	Q09152
Artemisia annua	Q9ZPJ3
Artemisia spiciformis	Q7XYS8
Bombyx mori	Q95P28
Choristoneura fumiferana	Q1XAB0
Culex quinquefasciatus	B0VZA8
Dendroctonus jeffreyi	Q56CY7
Drosophila melanogaster	Q7KN61
Epicauta gorhami	A0A075D844
Escherichia coli	P22939
Gallus gallus	P08836
Harpegnathos saltator	E2B2E9
Ips pini	Q58GE9
Mylabris cichorii	A0A0U2D675
Myzus persicae	B1PI49
Pediculus humanus corporis	E0VQL3
Tanacetum cinerariifolium	P0C565
Tetropium fuscum	J9QQQ8
Tribolium castaneum	D6WSE7

b)

GeneID	Evalue
g32607.t1	5.30E-72
g14484.t1	1.00E-63
g21383.t1	8.10E-32
g7366.t1	2.80E-25
g34061.t1	1.50E-23
g9107.t1	4.10E-18
g2704.t1	4.20E-18
g26044.t1	3.40E-10
g21682.t1	8.30E-07
g1822.t1	3.70E-05
g14822.t1	1.30E-04
g35512.t1	4.30E-01
g35890.t1	7.30E+00
g18384.t1	7.30E+00
g4554.t1	7.90E+00
g24024.t1	7.90E+00

Table S8: Primers used for amplification of IDS sequences from *P. citri* cDNA and for introducing mutations into *trans*IDS5 sequence, related to STAR Methods. For cloning primers (a), the cloning sites for pMAL-c5X expression vector are underlined (*Nco*I in forward primer and *Bam*HI in reverse primer), and for mutagenic primers (b), the mutated positions are underlined.

a) Cloning primers

Primer pair	Target
Fw 5’-ACATCCATGGCGTGTTTGGGTTCG-3’<br>Rv 5’-ATGTGGATCCTACTTCAGACGGTTCAAC-3’	*trans*IDS2
Fw 5’-ACATCCATGGCGAATTGTTCAAGTG-3’<br>Rv 5’-ATGATTCGGATCCTCAGAAATCCAC-3’	*trans*IDS4
Fw 5’-GCACCATGGGTCCATTATTC-3’<br>Rv 5’-GATAGGATCCTTAATTACTCCGTTTG-3’	*trans*IDS5
Fw 5’-ACATCCATGGATACTTTTGTTCGTTC-3’<br>Rv 5’-ATGTGGATCCTCATCTGCTATACTTTTTATTAAATTC-3’	*trans*IDS11
Fw 5’-ACATCCATGGTGAATTCGTTTCGAAG-3’<br>Rv 5’-ATCAGGATCCTTAAATTTCTCCCATCGC-3’	*trans*IDS12
Fw 5’-ACATCCATGGATATGGAAAGTGG-3’<br>Rv 5’-TAGTGGATCCTCAAGTATTCTCTTCC-3’	*trans*IDS16
Fw 5’-ACATCCATGGAAGACAAAGAAGC-3’<br>Rv 5’-ATGTGGATCCTTAATCGCTTTCGACC-3’	*trans*IDS17
Fw 5’-TTGACCATGGCATCTGAACTACCAGCAC-3’<br>Rv 5’-TGAAGGATCCTCACTCCACGTGAATATATAATTTG-3’	*cis*IDS1
Fw 5’-CTATCCATGGCGTCAAAAAATCAGCAACAAC-3’<br>Rv 5’-TCATGGATCCTTAGCTGTAATTCGGTGCTGTTC-3’	*cis*IDS8

b) Mutagenic primers

Primer pair	Mutation
Fw 5’- ATATTATGGATGGAGCTGAAACGAGAAG -3’<br>Rv 5’- CGTTCAATATTAAAAGAAATGCTTGAAGC -3’	D166N
Fw 5’- ACGATTATTTGGACTGTTTTGG -3’<br>Rv 5’- TCTGTATTTGGAAATAATGTCCC-3’	D308N
Fw 5’- TTGGACTGTTTTGGAGATGCAGATG -3’<br>Rv 5’- ATAATTGTCCTGTATTTGGAAATAATGTCC-3’	D309N
Fw 5’- TTGGAGATGCAGATGAAATTGGTAAAATC -3’<br>Rv 5’- AACAGTTCAAATAATCGTCCTGTATTTGG-3’	D312N
Fw 5’-AATCGTGGATTAGCTCTAGTCACCGC-3’<br>Rv 5’-TTTAGCTCCTCCGGGGACGTTATACTG-3’	K120A
Fw 5’- AATCGTGGATTAGCTCTAGTCACCGC-3’<br>Rv 5’-TTTCTCTCCTCCGGGGACGTTATAC-3’	K120E
Fw 5’- AATCGTGGATTAGCTCTAGTCACCGC-3’<br>Rv 5’- TTTCTGTCCTCCGGGGACGTTATAC -3’	K120Q

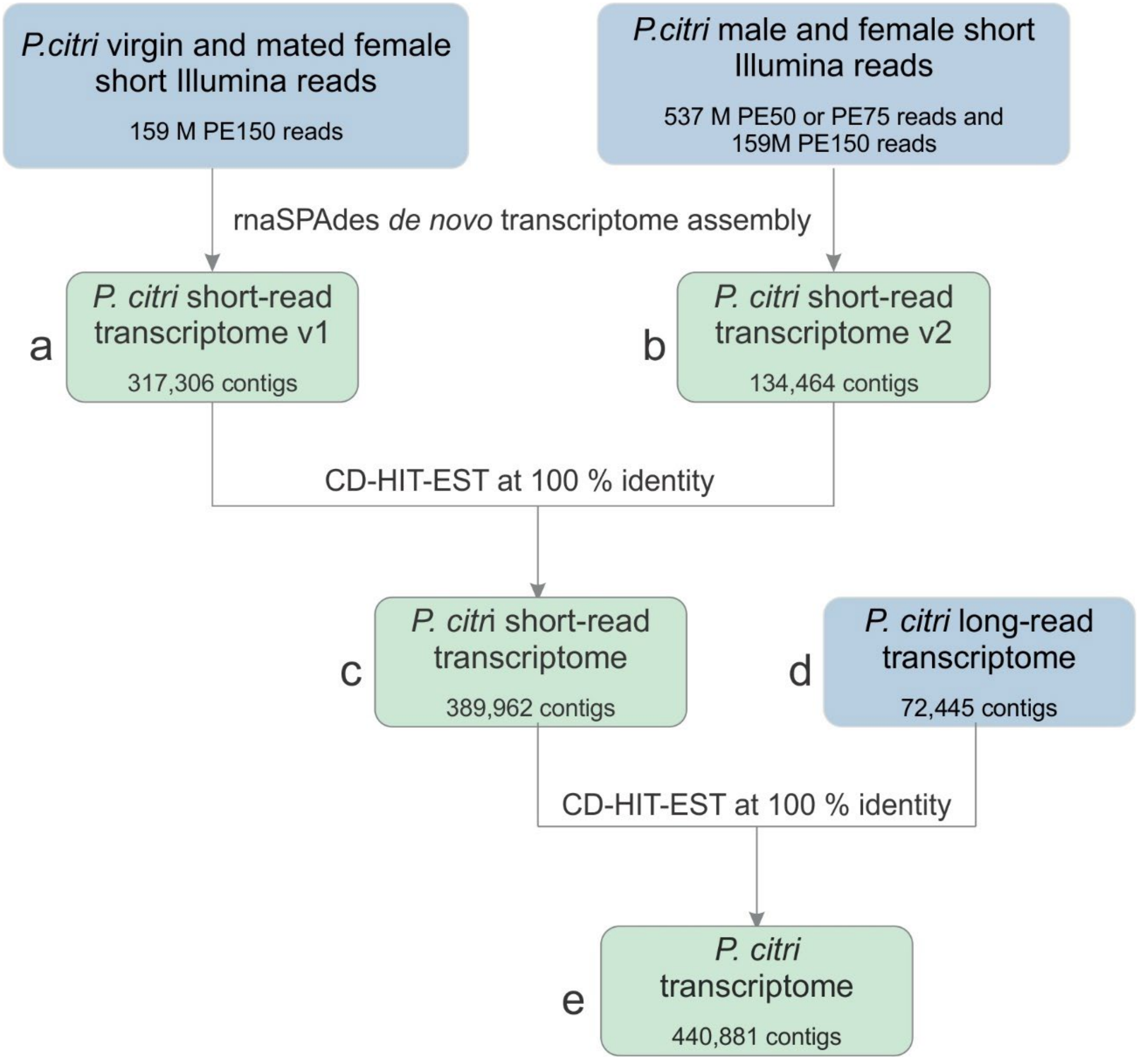

Figure S1: Construction of *P. citri* transcriptome resource, related to STAR Methods. Diagram of consolidation steps taken to combine the short-read (a, b, merged into c) and long-read (d) *P. citri* transcriptome resources into a comprehensive dataset of all assembled and sequenced putative *P. citri* transcripts (e). Raw sequencing data are in blue boxes. For details on the consolidation approach, see STAR Methods.

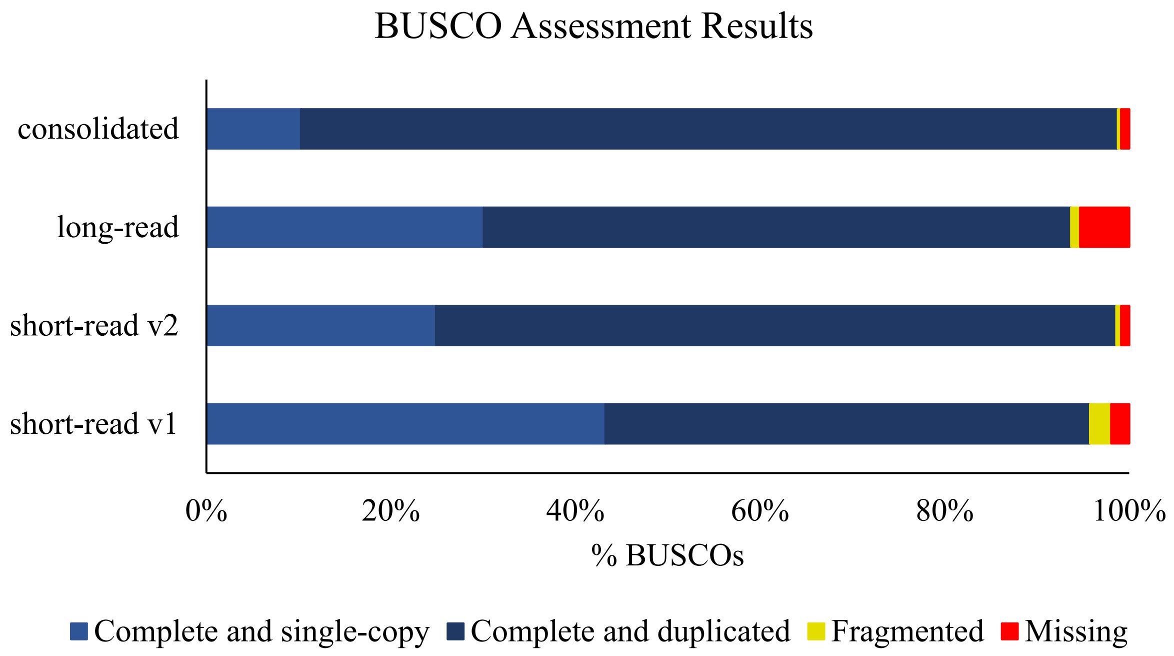

Figure S2: BUSCO assessment of transcriptome completeness, related to STAR Methods. BUSCO was run for the consolidated transcriptome dataset (Figure S1e), long-read Iso-Seq dataset (Figure S1d) and both short-read *de novo* assembled datasets (Figure S1a and b). Percents of complete BUSCOs are shown in blue (light for single-copy and dark for duplicates) and for fragmented and missing BUSCOs in yellow and red, respectively. N=1367

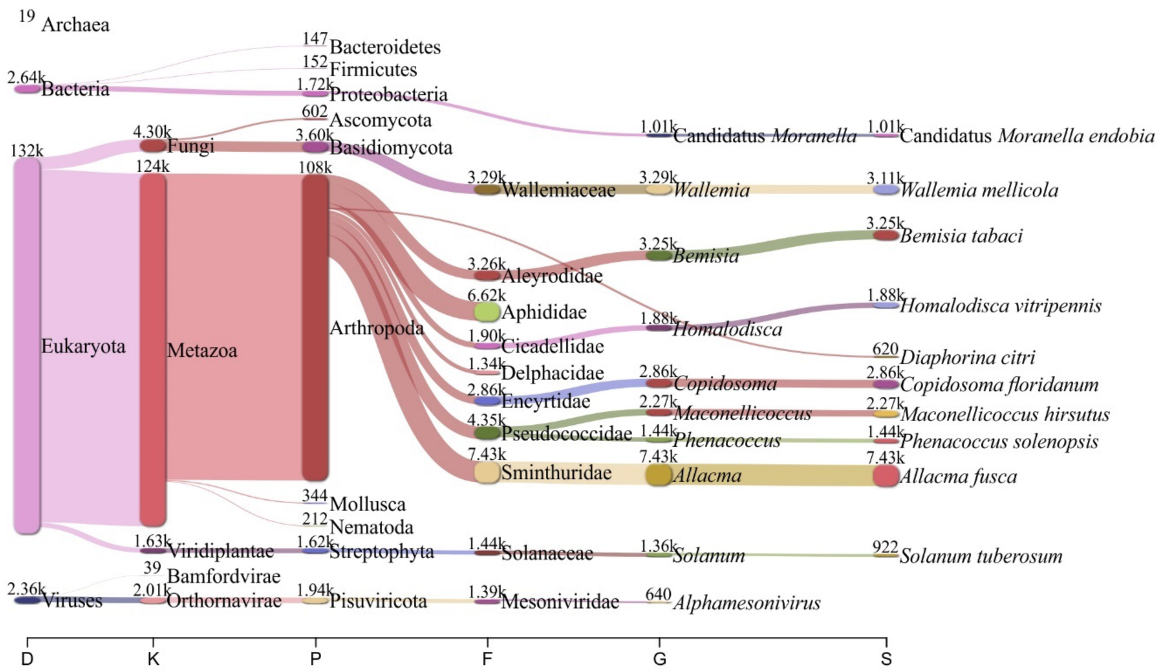

Figure S3: Taxonomic classification of the *P. citri* transcriptome dataset, related to STAR Methods. Sankey plot visualisation of mmseqs2 classification of translated coding sequences from the consolidated *P. citri* transcriptome (Figure S1e). For each taxa, the number of assigned sequences is given. Markings on the x-axis: D - domain, K - kingdom, P - phylum, F - family, G - genus, S - species.

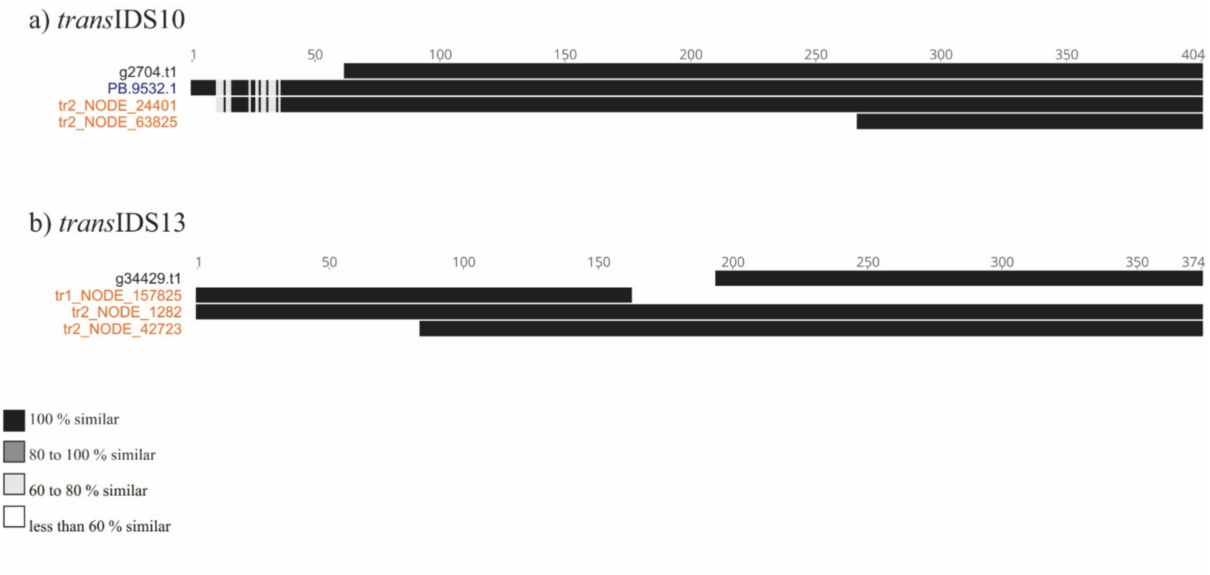

Figure S4: Alignments of sequence resources for *trans*IDS10 and *trans*IDS13, related to Figure 1. Multiple sequence alignments were done in MEGAX and visualised with Geneious software. Color-coded similarity is given in the legend below. Names of sequences originating from the *Pcitri*.v1 genome, long-read, and short-read assemblies are given in black, blue, and orange, respectively.

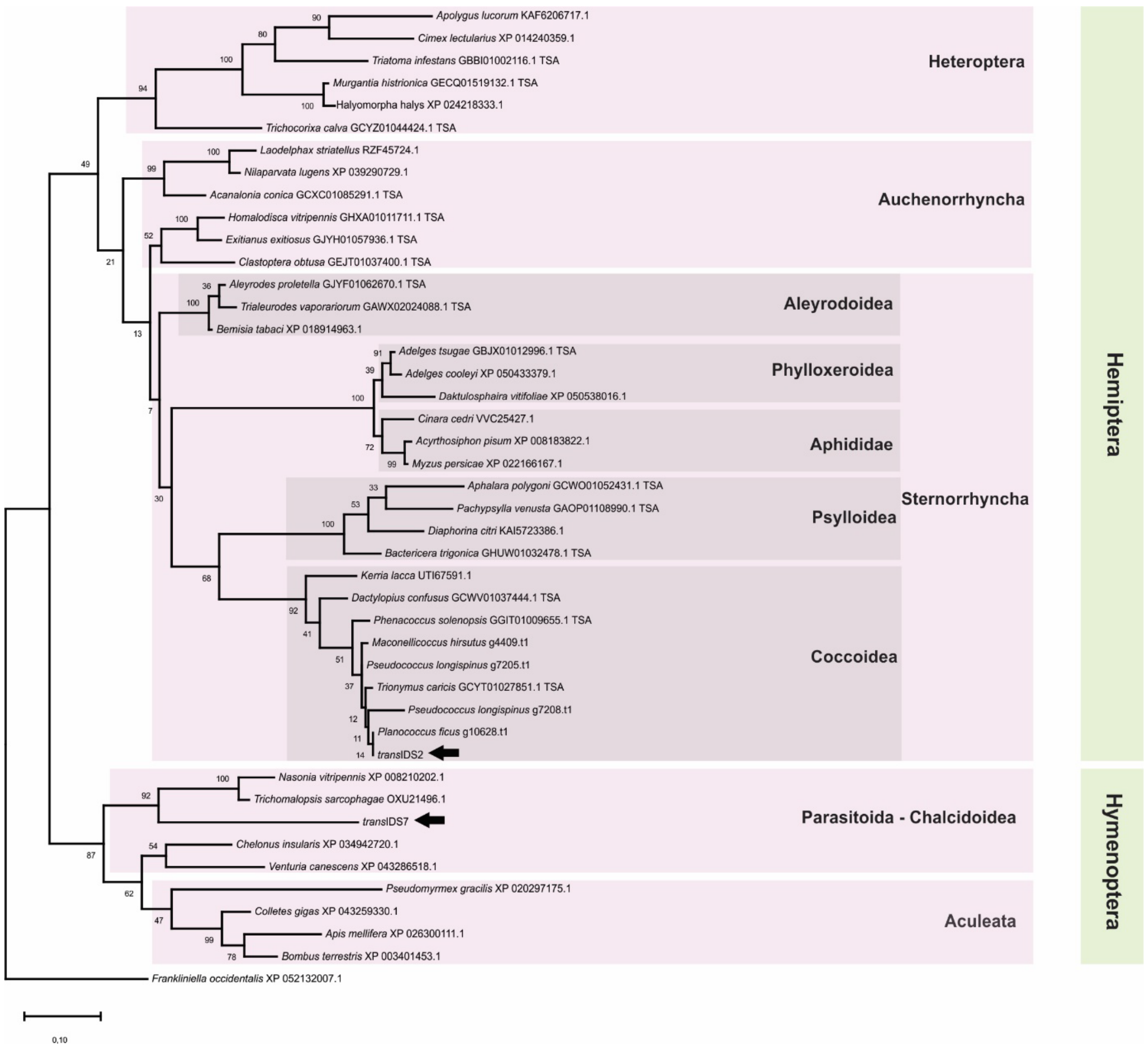

Figure S5: Phylogenetic tree of putative DPPS subunit 1 sequences from selected species with *trans*IDS2 and *trans*IDS7, related to Table 1. The tree is drawn to scale, with branch lengths measured in the number of substitutions per site (scale on the bottom left) and bootstrap values given at nodes. This analysis involved 44 amino acid sequences with a total of 603 positions in the final dataset. Positions of candidate sequences from this study (*trans*IDS2 and *trans*IDS7) are marked with black arrows. For each sequence, species of origin and GenBank, TSA, or gene model ID are given. Taxonomic classification of included species is marked with coloured blocks.

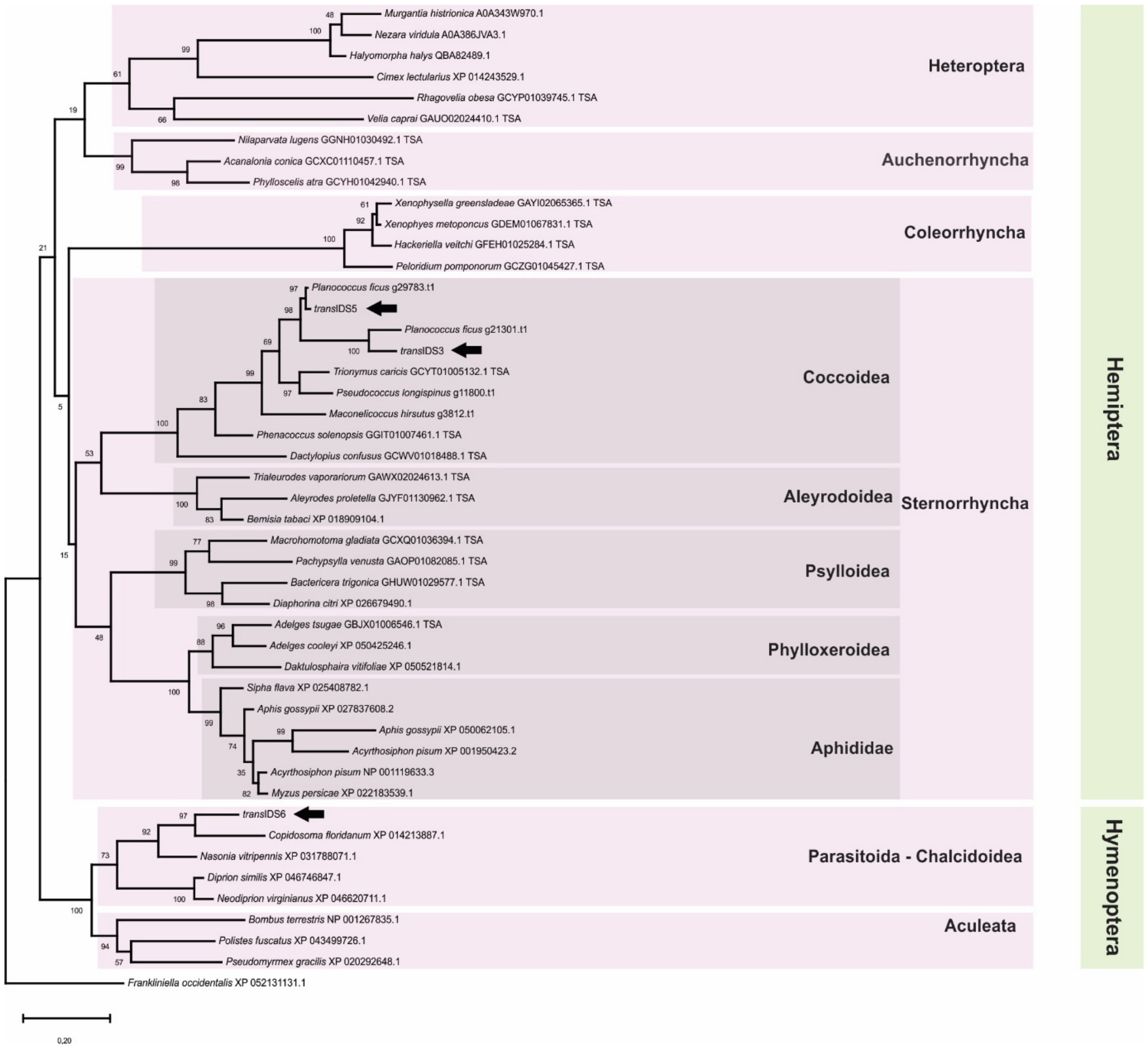

Figure S6: Phylogenetic tree of putative FPPS sequences from selected species with *trans*IDS3, *trans*IDS5, and *trans*IDS6, related to Table 1. The tree is drawn to scale, with branch lengths measured in the number of substitutions per site (scale on the bottom left) and bootstrap values given at nodes. This analysis involved 47 amino acid sequences with a total of 525 positions in the final dataset. Positions of candidate sequences from this study (*trans*IDS3, *trans*IDS5, and *trans*IDS6) are marked with black arrows. For each sequence, species of origin and GenBank, TSA, or gene model ID are given. Taxonomic classification of included species is marked with coloured blocks.

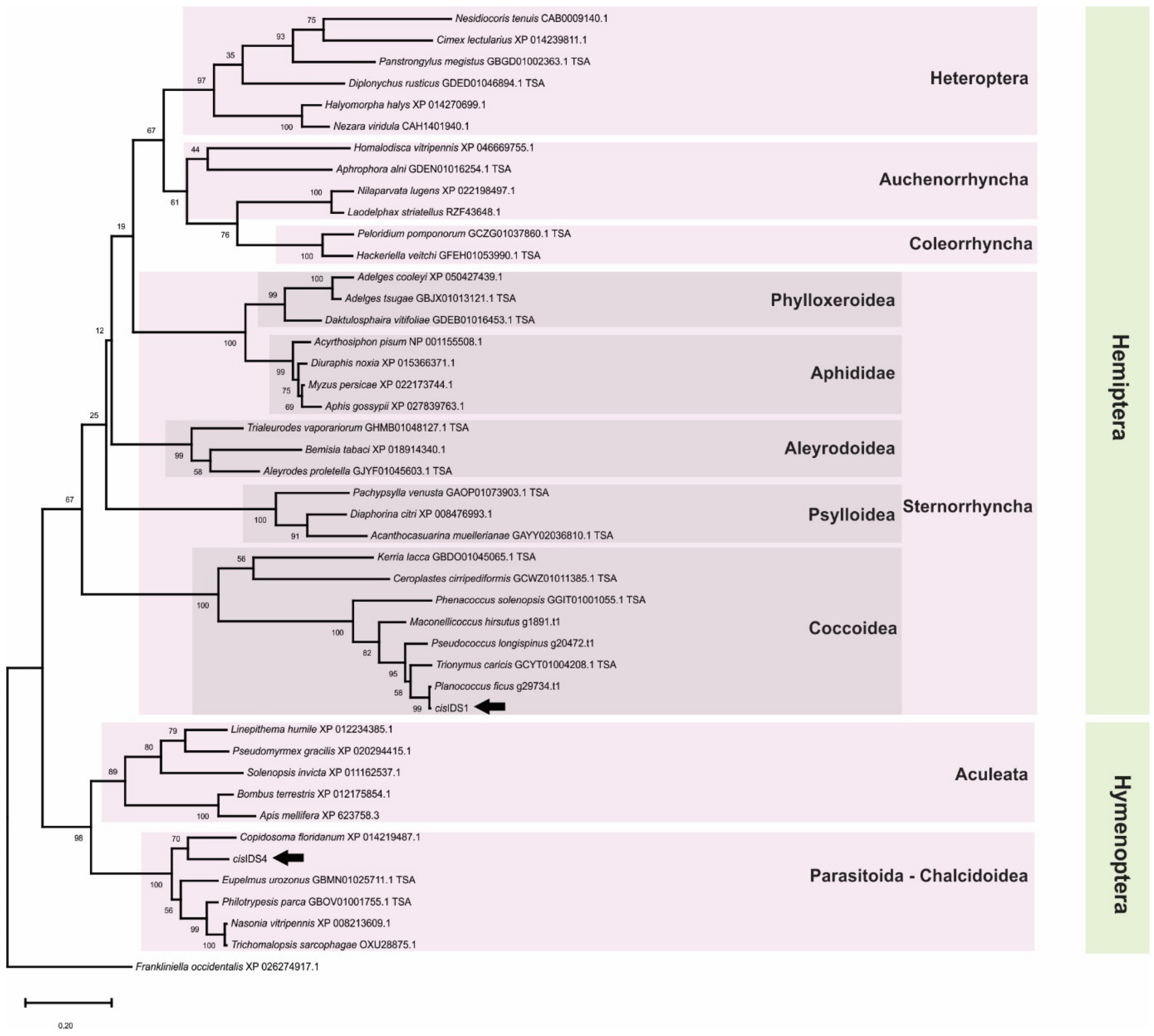

Figure S7: Phylogenetic tree of putative DHPPS catalytic subunit sequences from selected species with *cis*IDS1 and *cis*IDS4, related to Table 1. The tree is drawn to scale, with branch lengths measured in the number of substitutions per site (scale on the bottom left) and bootstrap values given at nodes. This analysis involved 45 amino acid sequences with a total of 418 positions in the final dataset. Positions of candidate sequences from this study (*cis*IDS1 and *cis*IDS4) are marked with black arrows. For each sequence, species of origin and GenBank, TSA, or gene model ID are given. Taxonomic classification of included species is marked with coloured blocks.

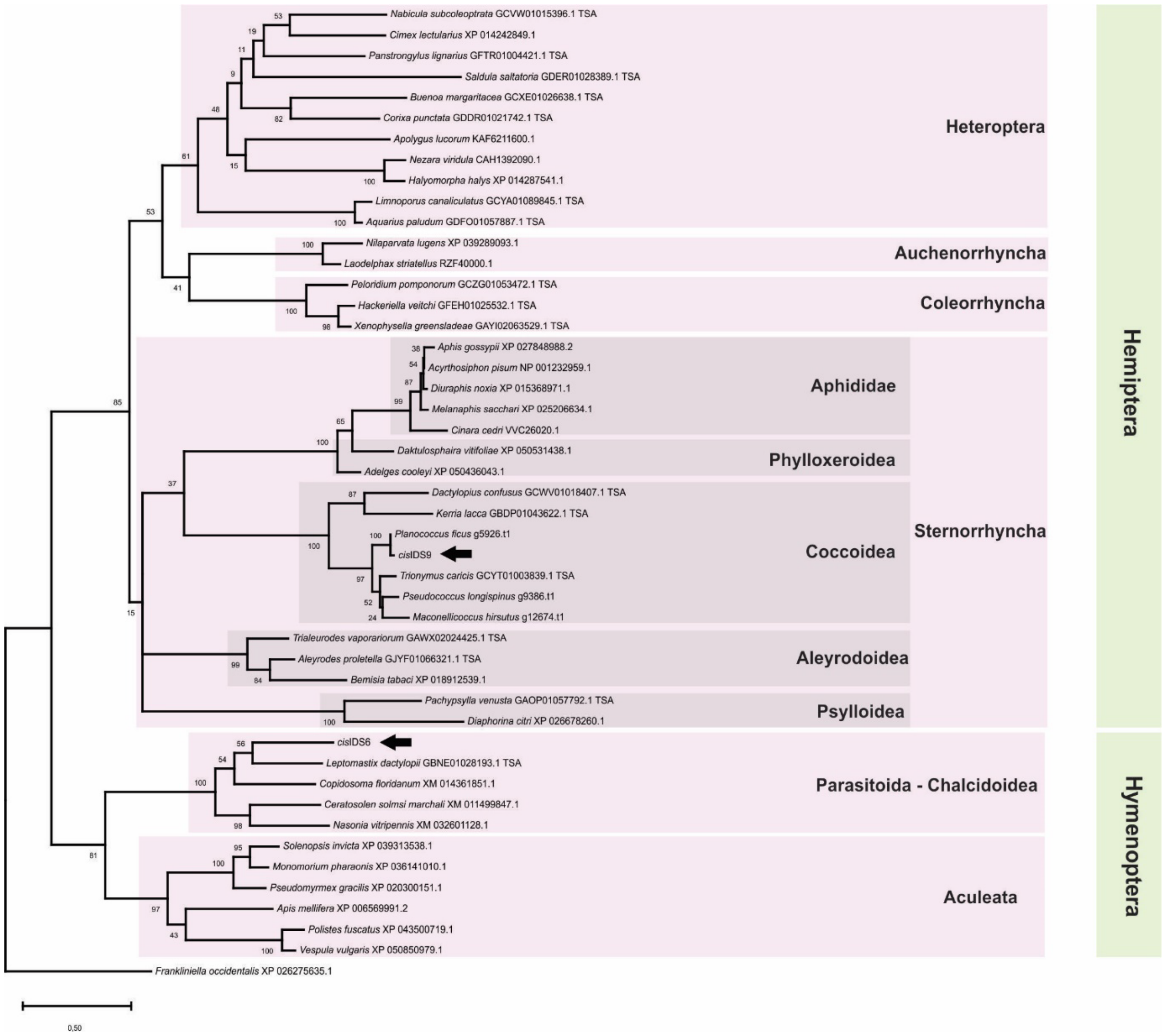

Figure S8: Phylogenetic tree of putative DHPPS regulatory subunit sequences from selected species with *cis*IDS6 and *cis*IDS9, related to Table 1. The tree is drawn to scale, with branch lengths measured in the number of substitutions per site (scale on the bottom left) and bootstrap values given at nodes. The analysis involved 47 amino acid sequences with a total of 559 positions in the final dataset. Positions of candidate sequences from this study (*cis*IDS6 and *cis*IDS9) are marked with black arrows. For each sequence, species of origin and GenBank, TSA, or gene model ID are given. Taxonomic classification of included species is marked with coloured blocks.

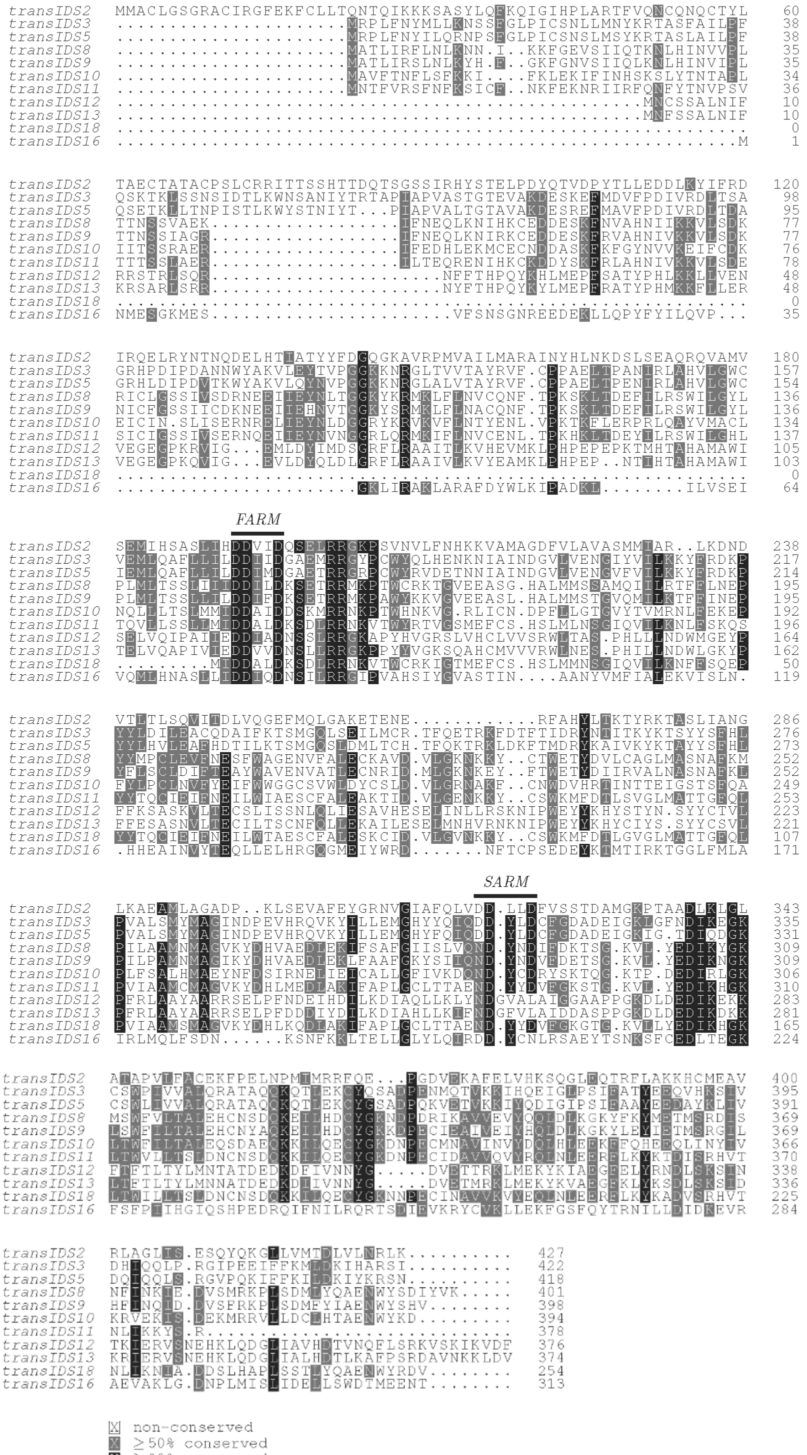

Figure S9: Multiple sequence alignment of *trans*-IDS *P. citri* candidates, related to Figure 2. Protein sequences of *trans*IDS2 (putative DHPPS), *trans*IDS3, *trans*IDS5, *trans*IDS8, *trans*IDS9, *trans*IDS10, *trans*IDS11, *trans*IDS12, *trans*IDS13, *trans*IDS18 (all putative FPPS or FPPS-like), and *trans*IDS16 (putative GGPPS). For more details on the candidates, see Table 1 and Figure 2. Positions of the first and second aspartate-rich motifs (FARM and SARM, respectively) are indicated. White, gray or black shading of the residues denote their conservation, as given in the legend below the alignment. Alignment and its visualisation were done in R using "msa" library and msaPrettyPrint function.

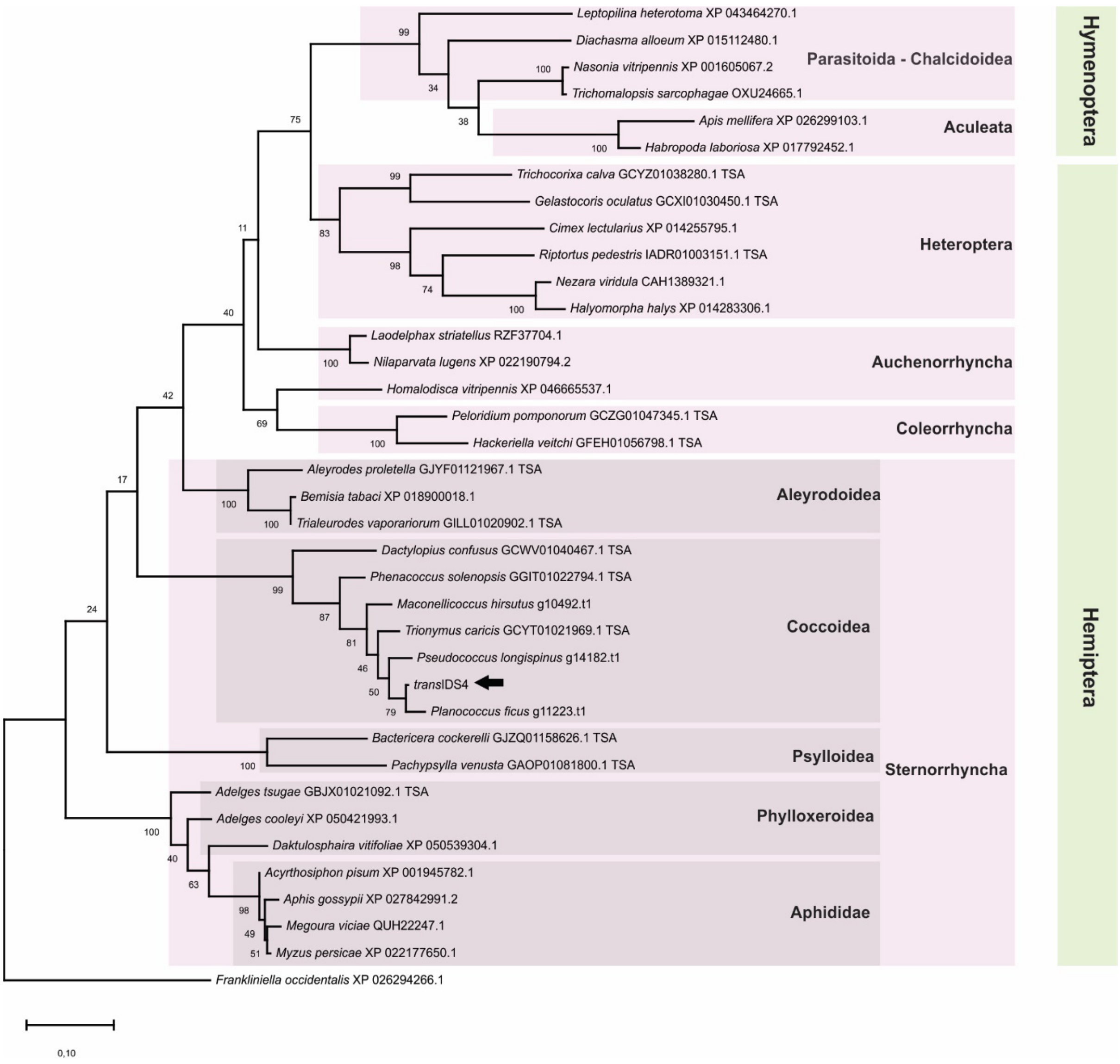

Figure S10: Phylogenetic tree of putative DPPS regulatory subunit sequences from selected species with *trans*IDS4, related to Table 1. The tree is drawn to scale, with branch lengths measured in the number of substitutions per site (scale on the bottom left) and bootstrap values given at nodes. The analysis involved 37 amino acid sequences with a total of 474 positions in the final dataset. The position of candidate sequence from this study (*trans*IDS4) is marked with a black arrow. For each sequence, species of origin and GenBank, TSA, or gene model ID are given. Taxonomic classification of included species is marked with coloured blocks.

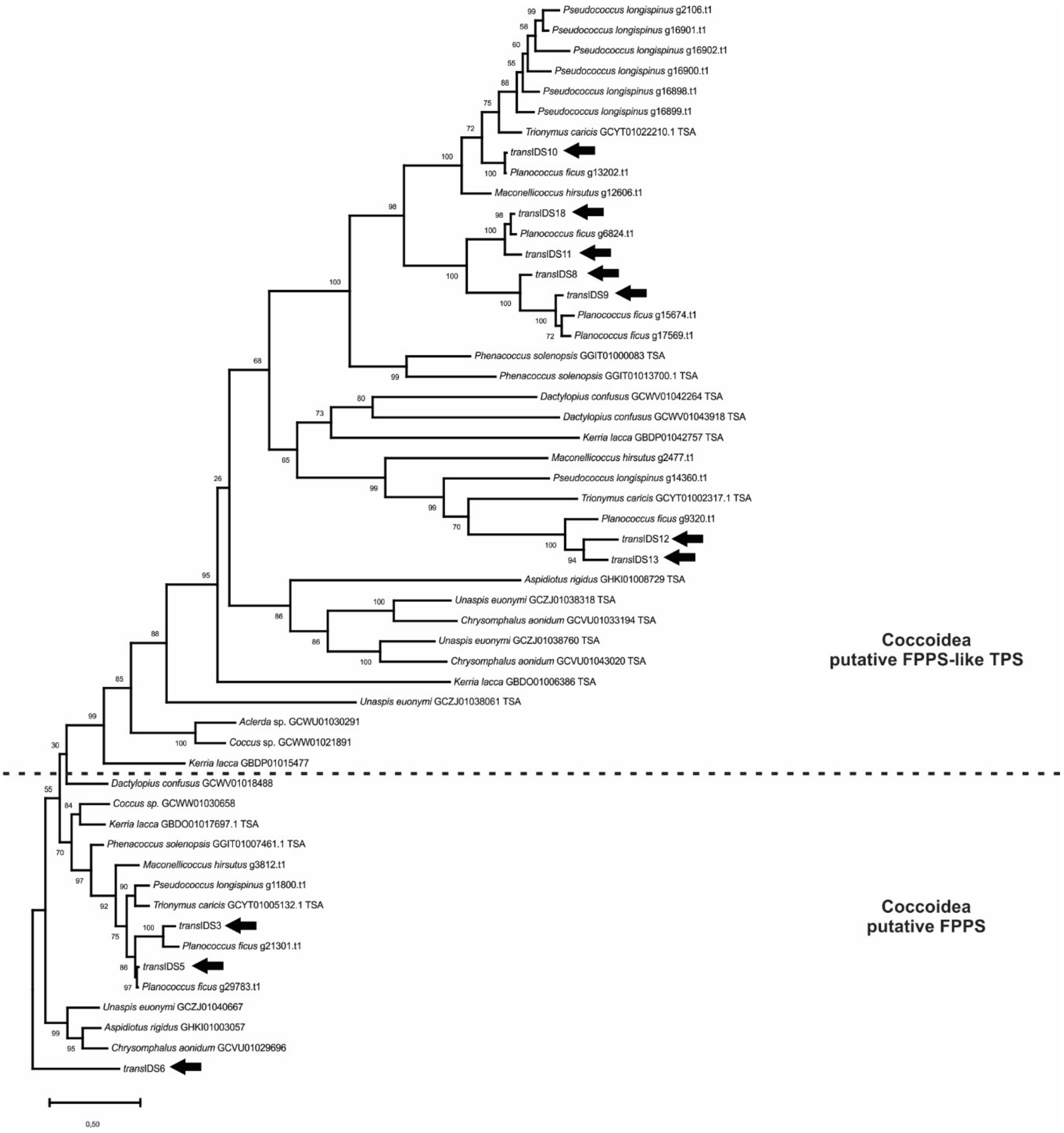

Figure S11: Phylogenetic tree of putative FPPS and FPPS-like sequences from Coccoidea species, related to Table 1. The tree is drawn to scale, with branch lengths measured in the number of substitutions per site (scale on the bottom left) and bootstrap values given at nodes. The analysis involved 53 amino acid sequences with a total of 826 positions in the final dataset. Positions of candidate sequences from this study (*trans*IDS3, *trans*IDS5, *trans*IDS6, *trans*IDS8, *trans*IDS9, *trans*IDS10, *trans*IDS11, *trans*IDS12, *trans*IDS13, and *trans*IDS18) are marked with black arrows. For each sequence, species of origin and GenBank, TSA, or gene model ID are given. The delineation between the FPPS and FPPS-like sequences is based on Rebholz et al., 2023[33]. Tree is rooted with *trans*IDS6, the identified FPPS with similarity to sequences from Chalcidoidea wasps (Hymenoptera).

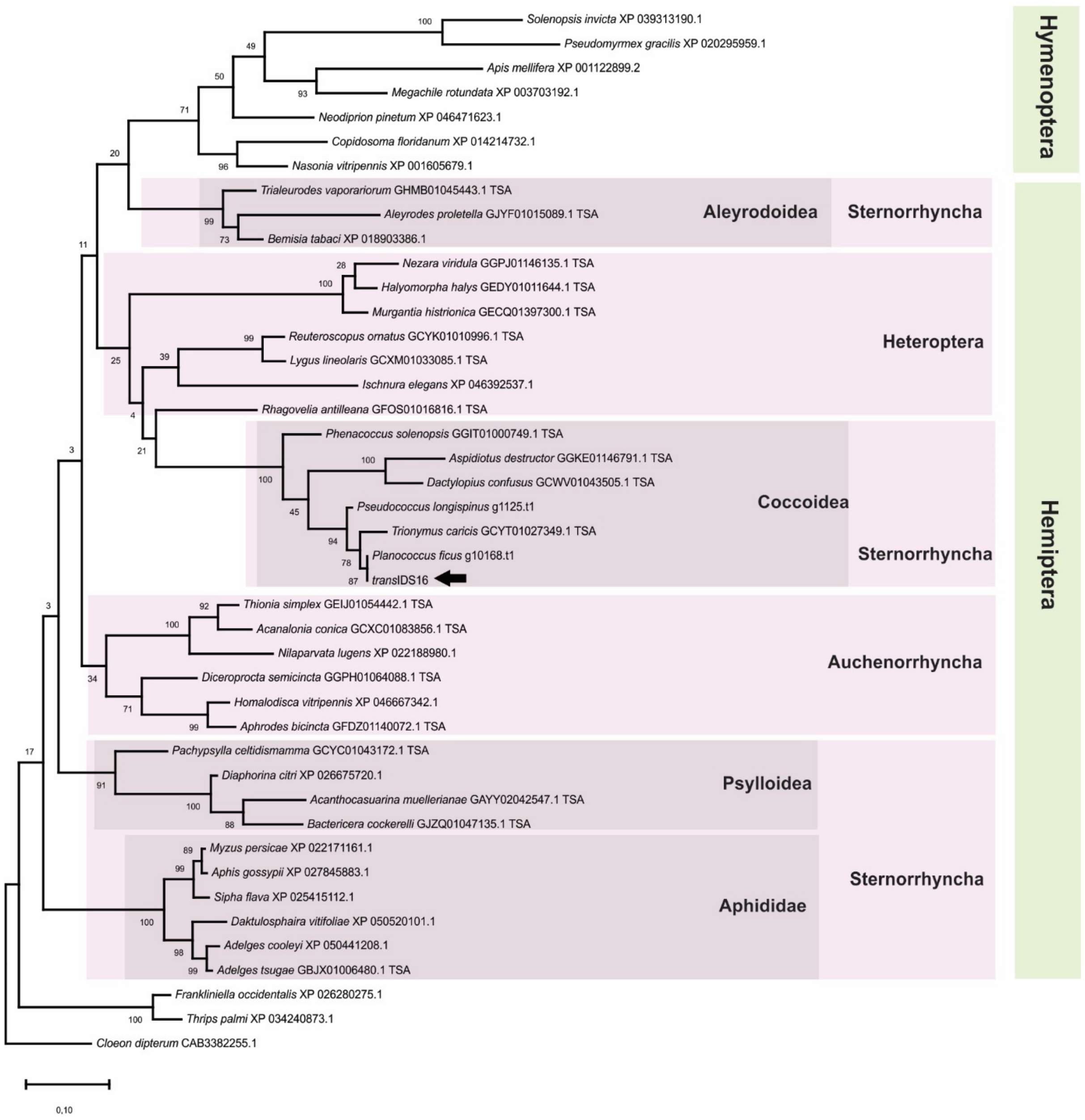

Figure S12: Phylogenetic tree of putative GGPPS sequences from selected species with *trans*IDS16, related to Table 1. The tree is drawn to scale, with branch lengths measured in the number of substitutions per site (scale on the bottom left) and bootstrap values given at nodes. The analysis involved 43 amino acid sequences with a total of 400 positions in the final dataset. The position of candidate sequence from this study (*trans*IDS16) is marked with a black arrow. For each sequence, species of origin and GenBank, TSA, or gene model ID are given. Taxonomic classification of included species is marked with coloured blocks.

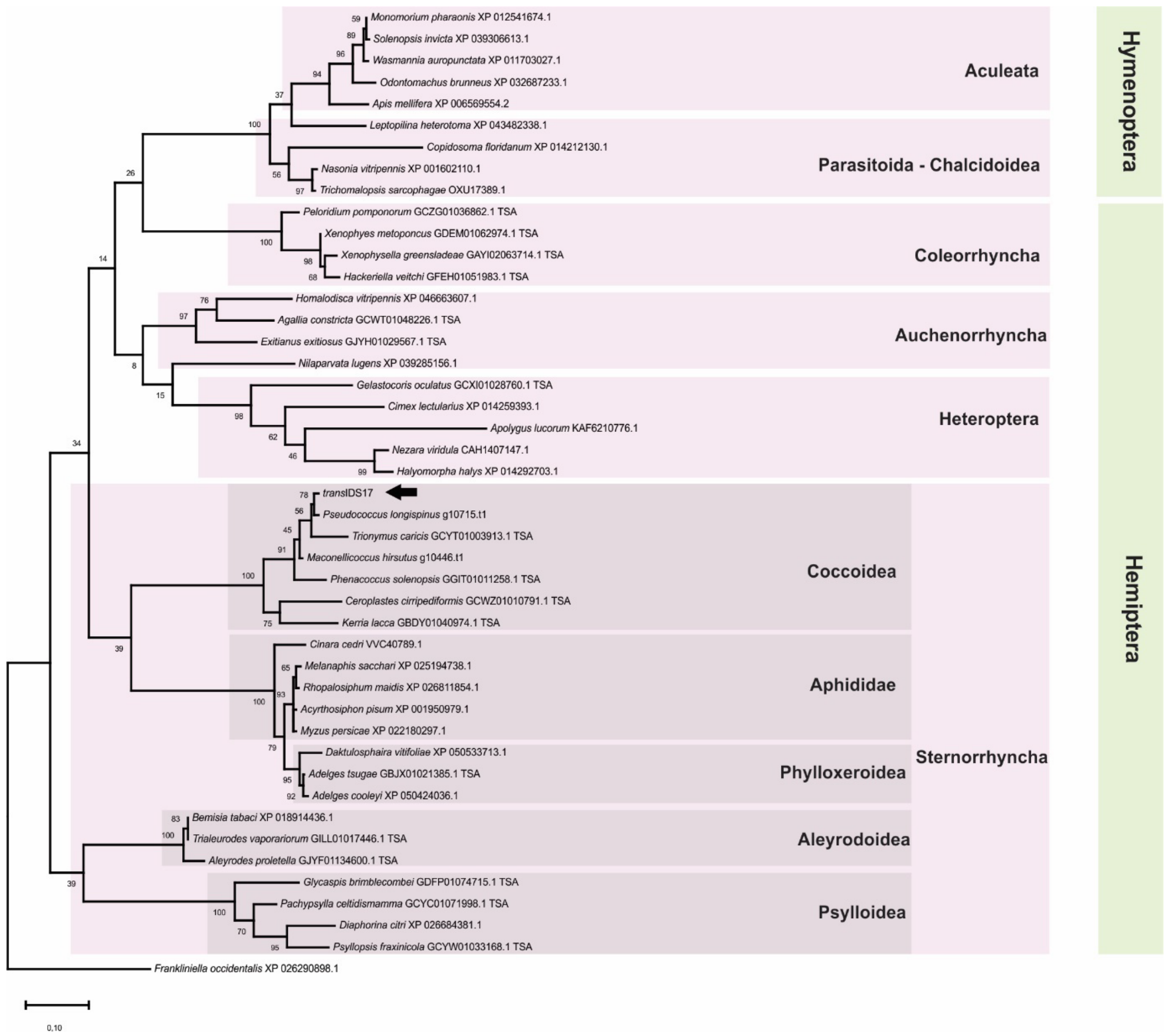

Figure S13: Phylogenetic tree of putative otubain-like sequences from selected species with *trans*IDS17, related to Table 1. The tree is drawn to scale, with branch lengths measured in the number of substitutions per site (scale on the bottom left) and bootstrap values given at nodes. The analysis involved 45 amino acid sequences with a total of 344 positions in the final dataset. The position of candidate sequence from this study (*trans*IDS17) is marked with a black arrow. For each sequence, species of origin and GenBank, TSA, or gene model ID are given. Taxonomic classification of included species is marked with coloured blocks.

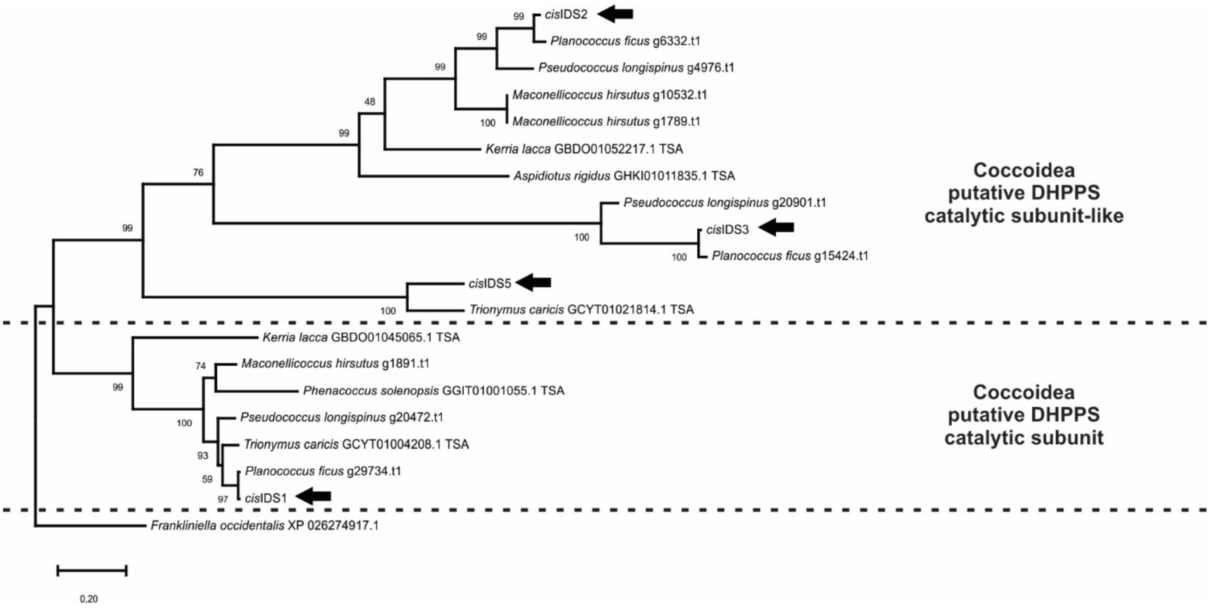

Figure S14: Phylogenetic tree of putative DHPPS and DHPPS-like sequences from selected Coccoidea species, related to Table 1. The tree is drawn to scale, with branch lengths measured in the number of substitutions per site (scale on the bottom left) and bootstrap values given at nodes. The analysis involved 45 amino acid sequences with a total of 344 positions in the final dataset. Positions of candidate sequences from this study (*cis*IDS1, *cis*IDS2, *cis*IDS3, and *cis*IDS5) are marked with black arrows. For each sequence, species of origin and GenBank, TSA, or gene model ID are given.

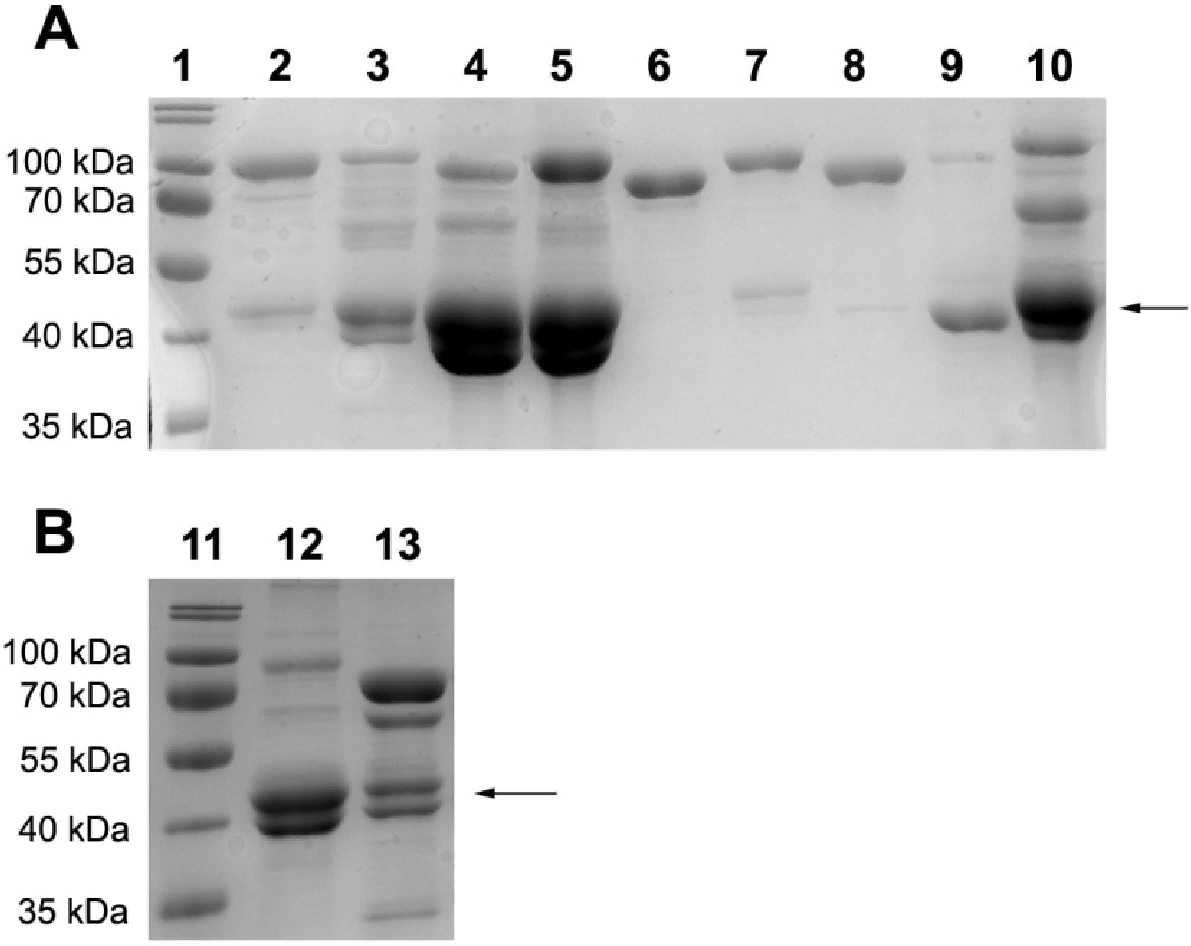

Figure S15: SDS-PAGE analysis of candidate proteins expressed in *E. coli* in fusion with maltose-binding domain and purified on amylose resin, related to Table 1. A) IDS of *trans* type. Lane 1: MW ladder; lane 2: *trans*IDS5 (90.8 kDa); lane 3: *trans*IDS3 (92 kDa); lane 4: *trans*IDS11 (86.3 kDa); lane 5: *trans*IDS2 (90.8 kDa); lane 6: *trans*IDS17 (74.9 kDa); lane 7: *trans*IDS16 (79.5 kDa); lane 8: *trans*IDS4 (84.1 kDa); lane 9: *trans*IDS10 (84.1 kDa); lane 10: *trans*IDS12 (89.7 kDa). B) IDS of *cis* type. Lane 11: MW ladder; lane 12: *cis*IDS1 (81.7 kDa); lane 13: *cis*IDS8 (71.6 kDa). For more details on the candidates, see Table 1. Arrows indicate the bands corresponding to partially split maltose-binding domain (40.4 kDa).

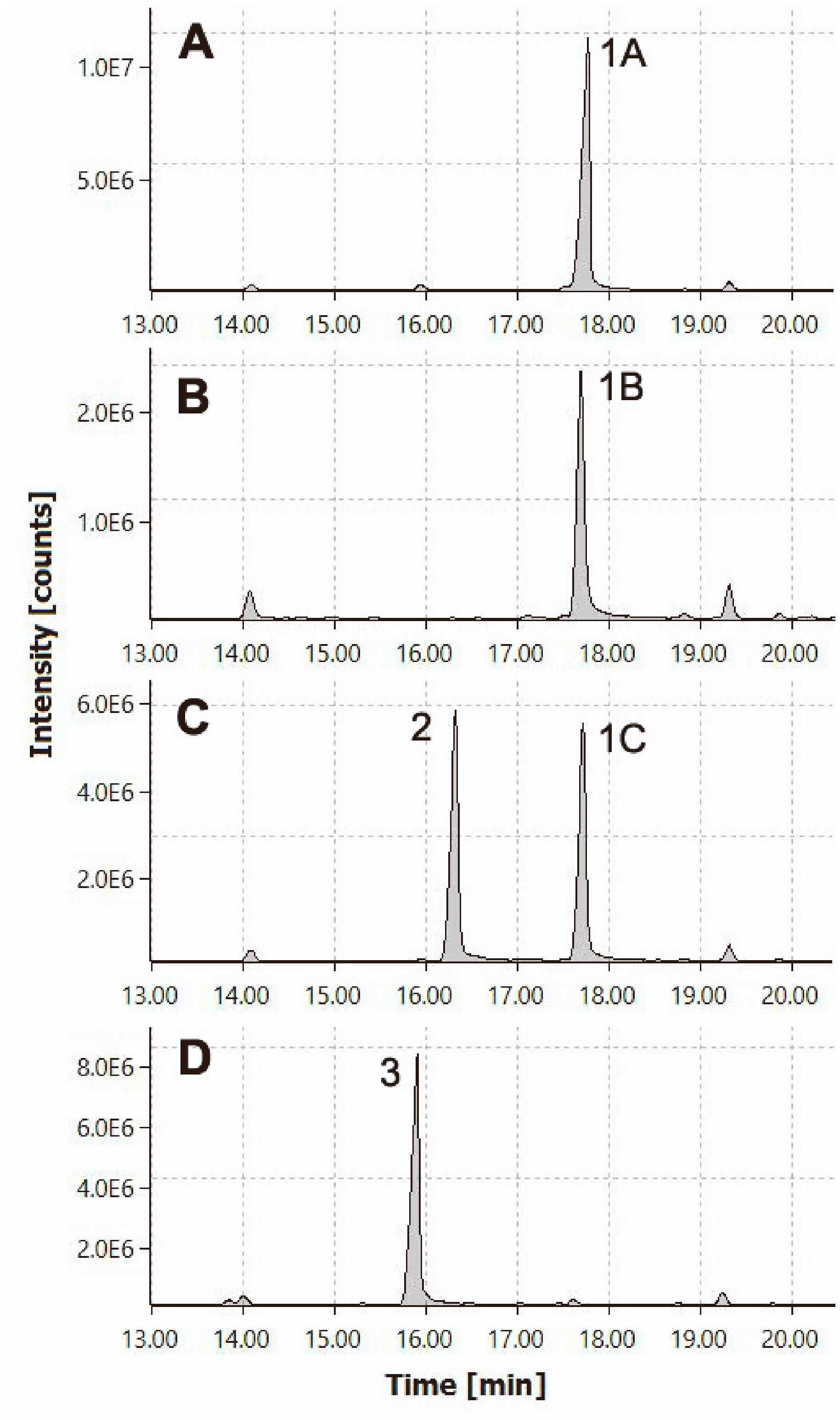

Figure S16: Identification of regular monoterpenes synthesysed by *trans*-IDS enzymes *trans*IDS5 and *trans*IDS3 from *P. citri*, related to Figure 3. The proteins were incubated with IPP and DMAPP, and dephosphorylated products were analysed by GC-MS (see STAR Methods). A) Standard GPP (dephosphorylated) – geraniol (peak 1A); B) *trans*IDS5 – geraniol (peak 1B); C) *trans*IDS3 – geraniol (peak 1C) and iso-geraniol (peak 2); D) standard NPP (dephosphorylated) – nerol (peak 3). EI-MS data of the peaks are shown in Figures S17 and S18.

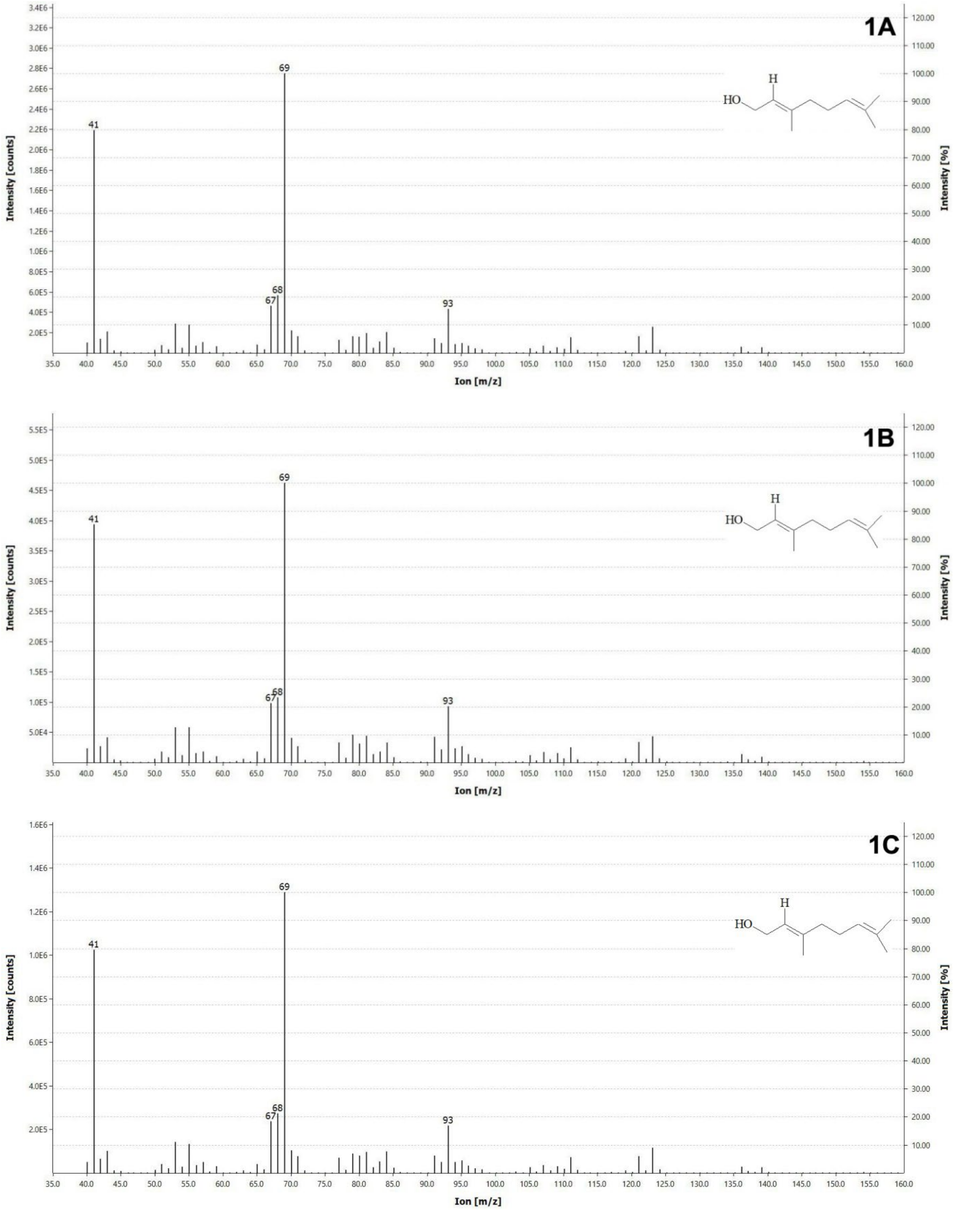

Figure S17: EI mass spectra of geraniol generated by dephosphorylation of standard GPP (1A) and products of *trans*IDS5 (1B) and *trans*IDS3 (1C), related to Figure 3. For chromatograms, see Figure S16.

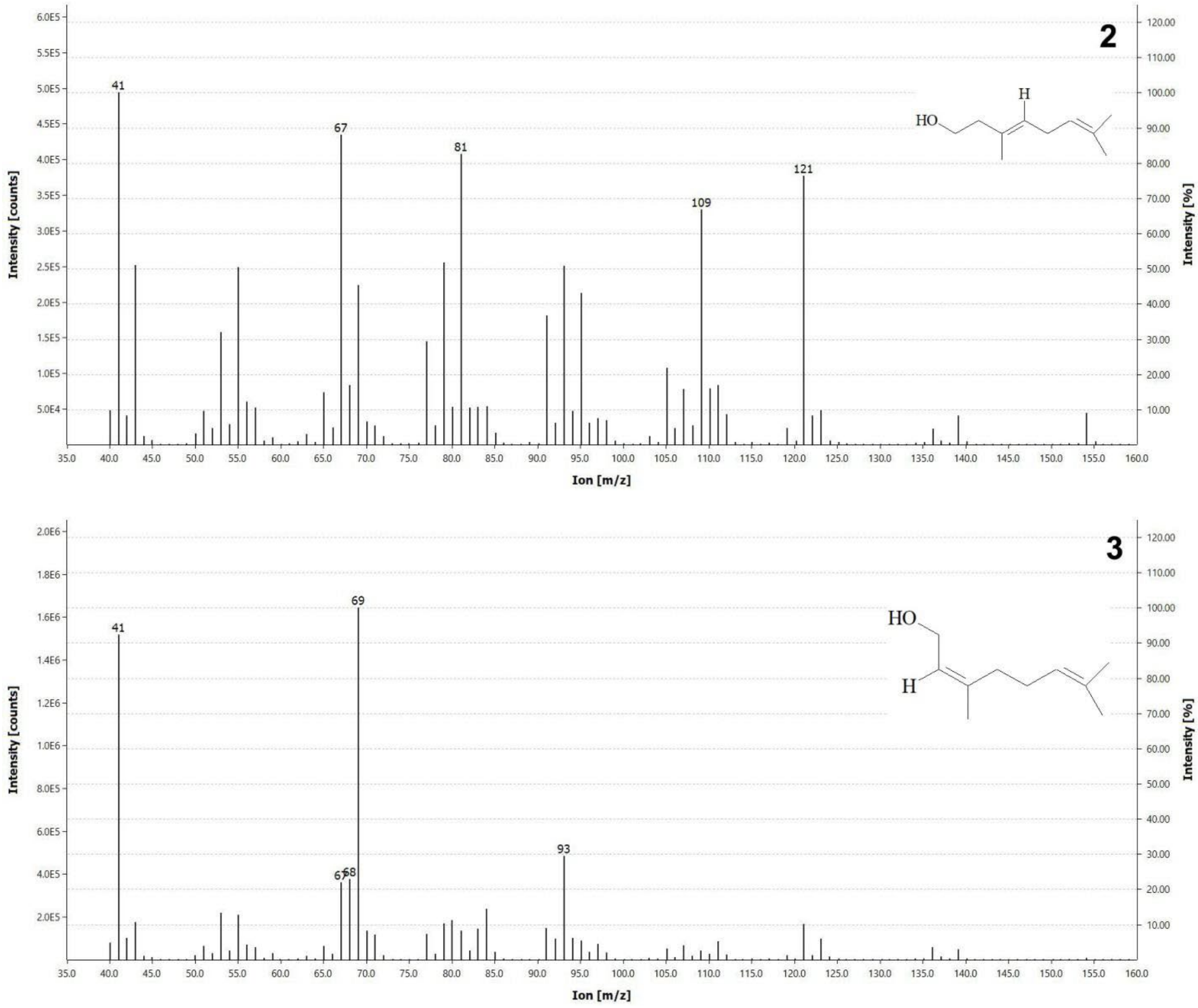

Figure S18: EI mass spectra of dephosphorylated derivatives of product of *trans*IDS3 (2, iso-geraniol) and standard NPP (3, nerol), related to Figure 3. For chromatograms, see Figure S16.

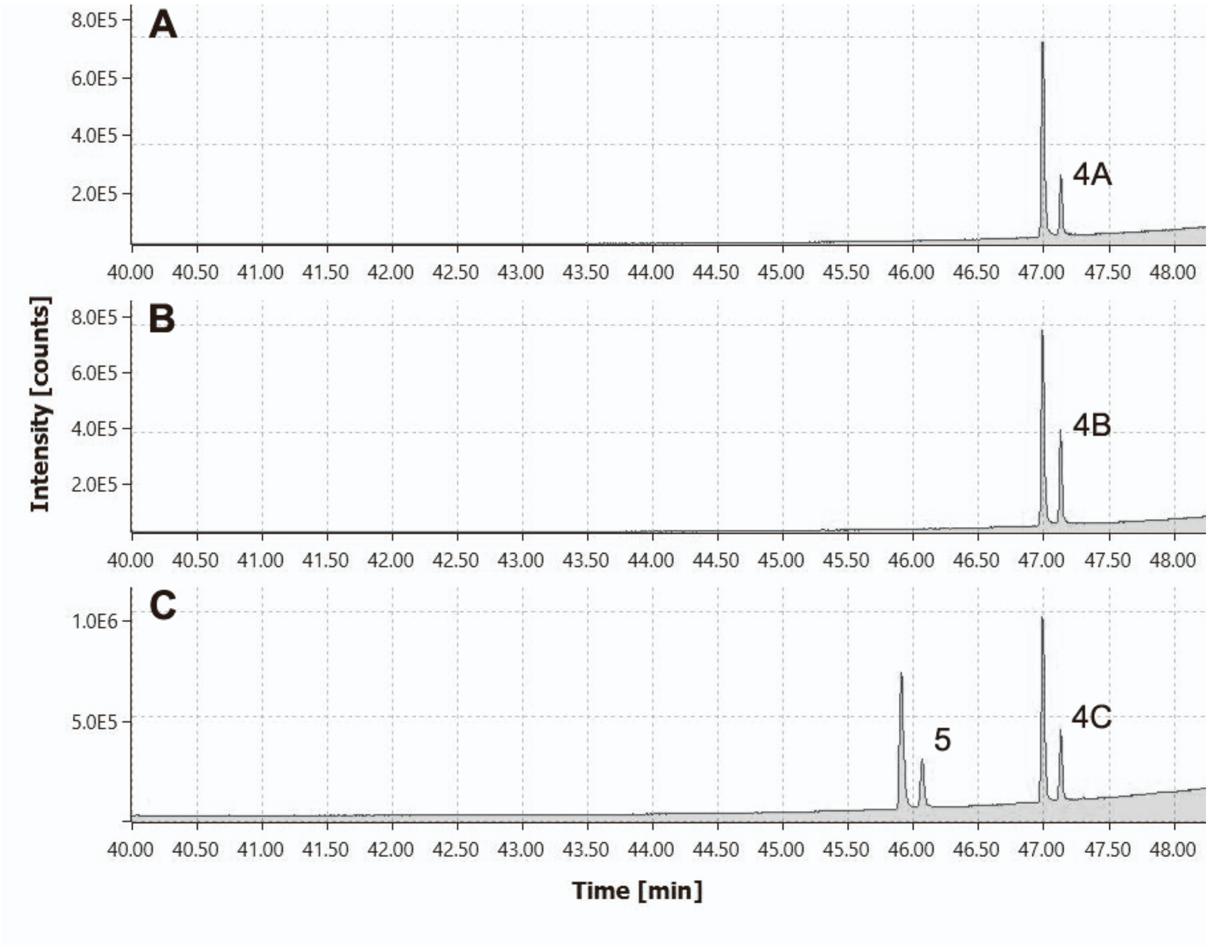

Figure S19: Identification of regular sesquiterpenes synthesised by *trans*-IDS enzymes *trans*IDS5 and *trans*IDS3 from *P. citri*, related to Figure 3. The proteins were incubated with IPP and DMAPP, and dephosphorylated products were analysed by GC-MS. (A) FPPS from *Tanacetum cinerariifolium* (known to produce *trans*-farnesyl diphosphate) – *trans*-farnesol (peak 4A); (B) *trans*IDS5 – *trans*-farnesol (peak 4B); (C) *trans*IDS3 – *trans*-farnesol (peak 4C) and farnesol isomer (peak 5). EI-MS data of the peaks are shown in Figures S20 and S21.

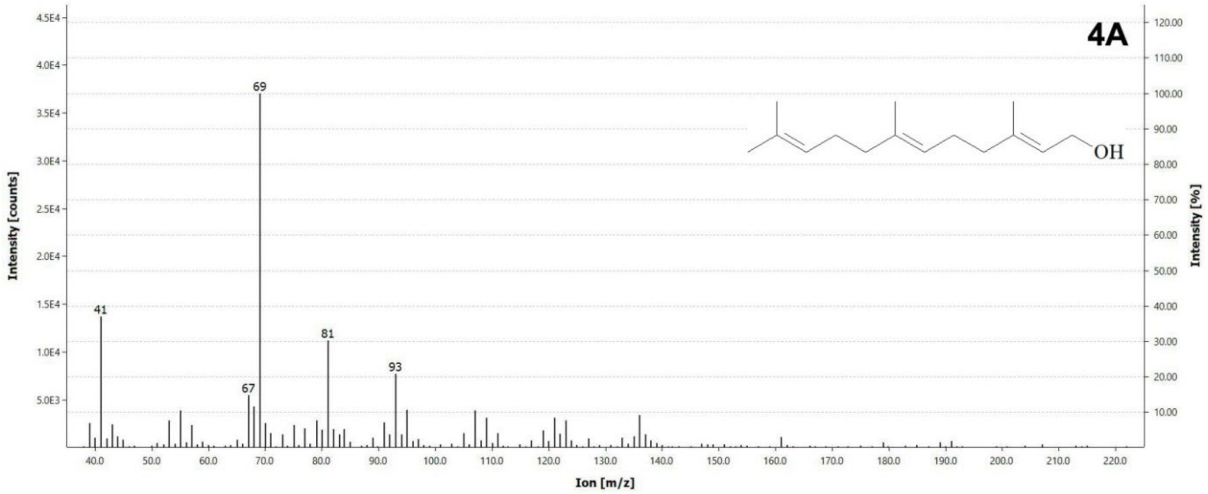

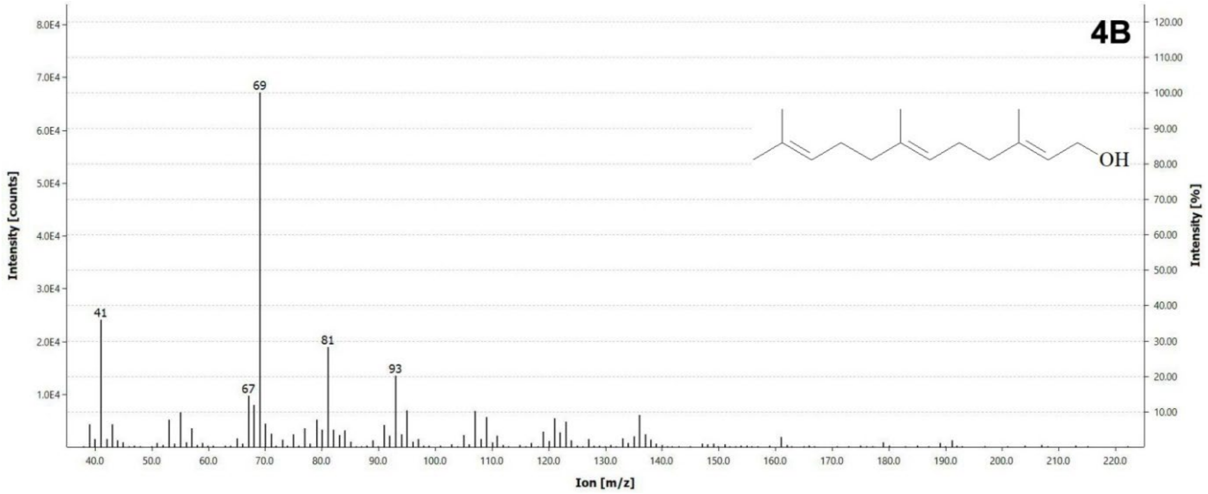

Figure S20: EI mass spectra of *trans*-farnesol generated by dephosphorylation of products of FPPS from *Tanacetum cinerariifolium* known to produce *trans*-farnesyl diphosphate (4A) and *trans*IDS5 (4B), related to Figure 3. For chromatograms, see Figure S19.

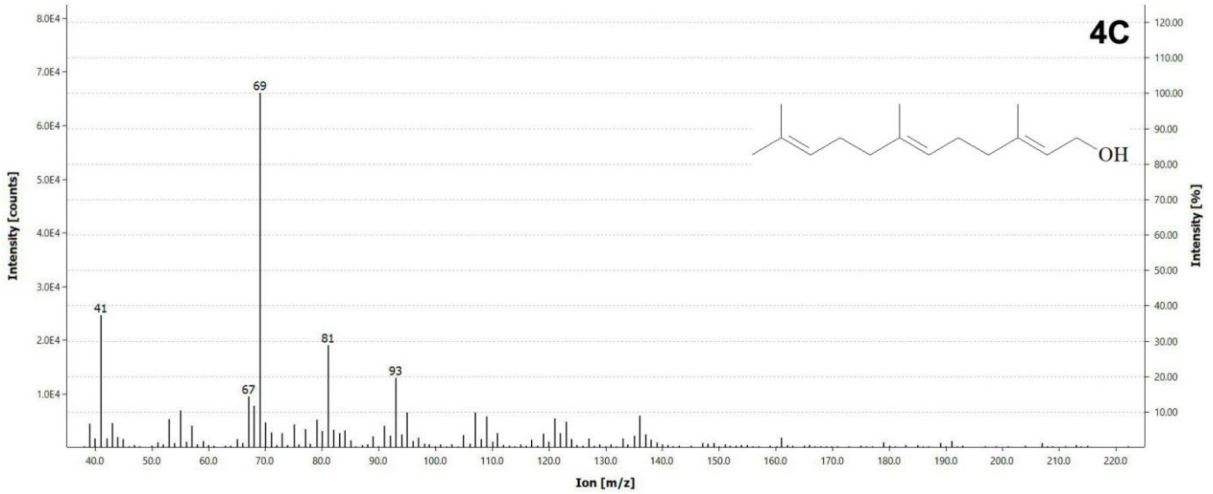

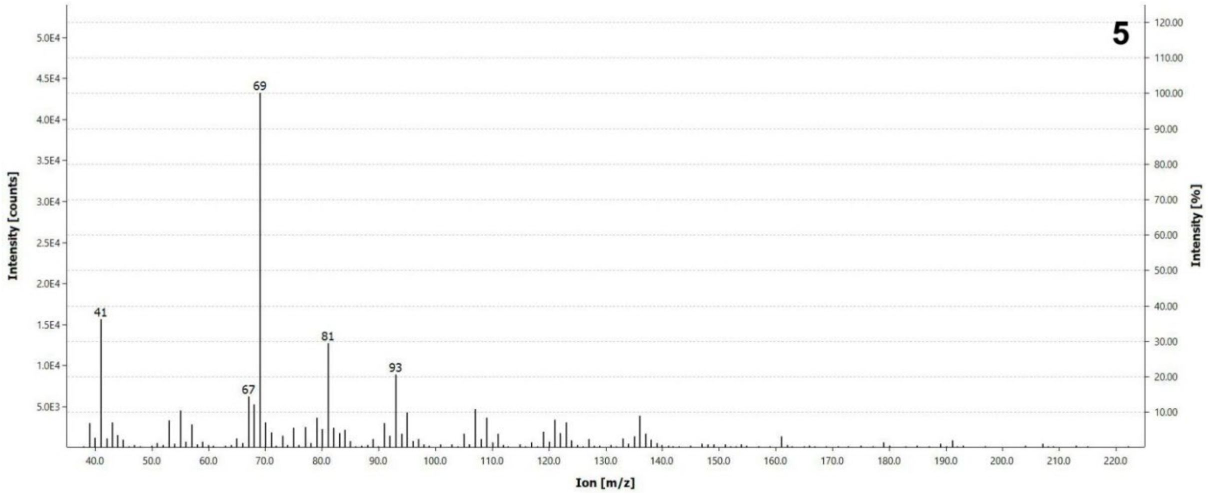

Figure S21: EI mass spectra of dephosphorylated products of *trans*IDS3, *trans*-farnesol (4C) and farnesol isomer (5), related to Figure 3. For chromatograms, see Figure S19.

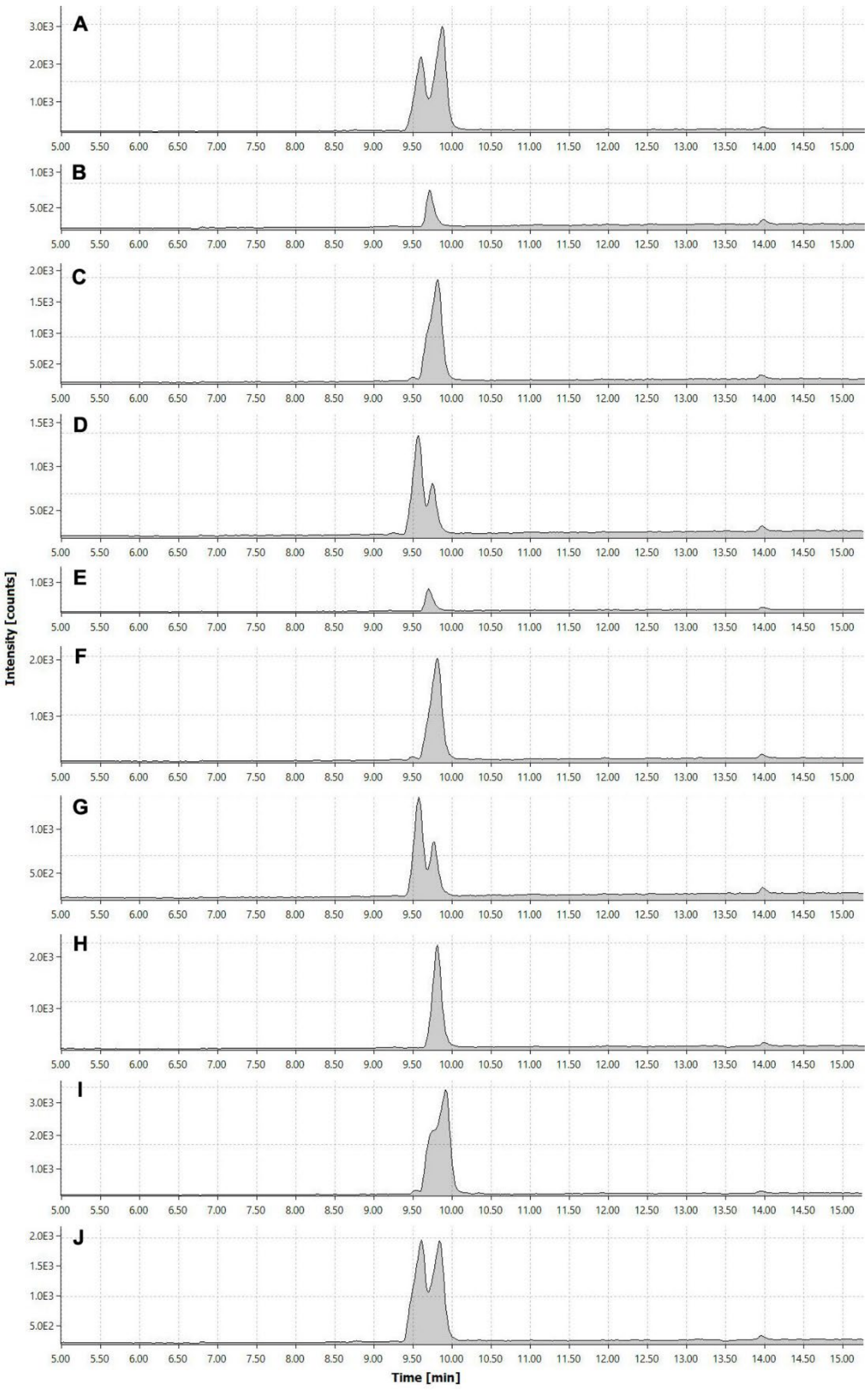

Figure S22: Identification of regular monoterpene diphosphates synthesised by *trans*-IDS enzymes *trans*IDS2, *trans*IDS11, and *trans*IDS17 from *P. citri*, related to Figure 3. The proteins were incubated with IPP and DMAPP, and the products were analysed by LC-MS. (A) Standard NPP and GPP; (B) product of *trans*IDS2; (C) product of *trans*IDS2 and GPP; (D) product of *trans*IDS2 and NPP; (E) product of *trans*IDS11; (F) product of *trans*IDS11 and GPP; (G) product of *trans*IDS11 and NPP; (H) product of *trans*IDS17; (I) product of *trans*IDS17 and GPP; (J) product of *trans*IDS17 and NPP.

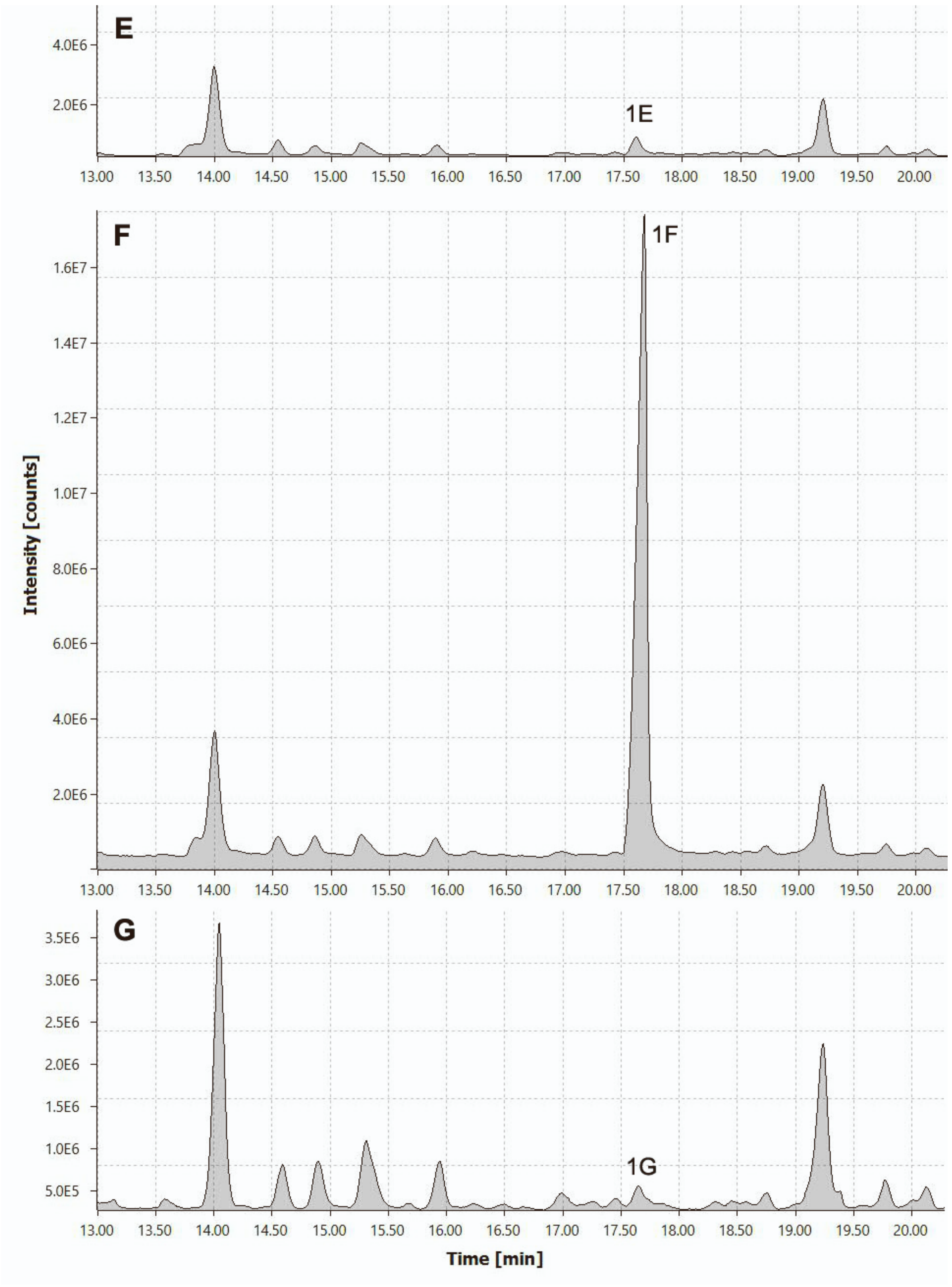

Figure S23: Identification of regular monoterpenes synthesysed by *trans*-IDS enzymes *trans*IDS2, *trans*IDS11, and *trans*IDS17 from *P. citri*, related to Figure 3. The proteins were incubated with IPP and DMAPP, and dephosphorylated products were analysed by GC-MS. E) *trans*IDS11 – geraniol (peak 1E); F) *trans*IDS2 – geraniol (peak 1F); G) *trans*IDS17 – geraniol (peak 1G). EI-MS data of the peaks are shown in Figure S24.

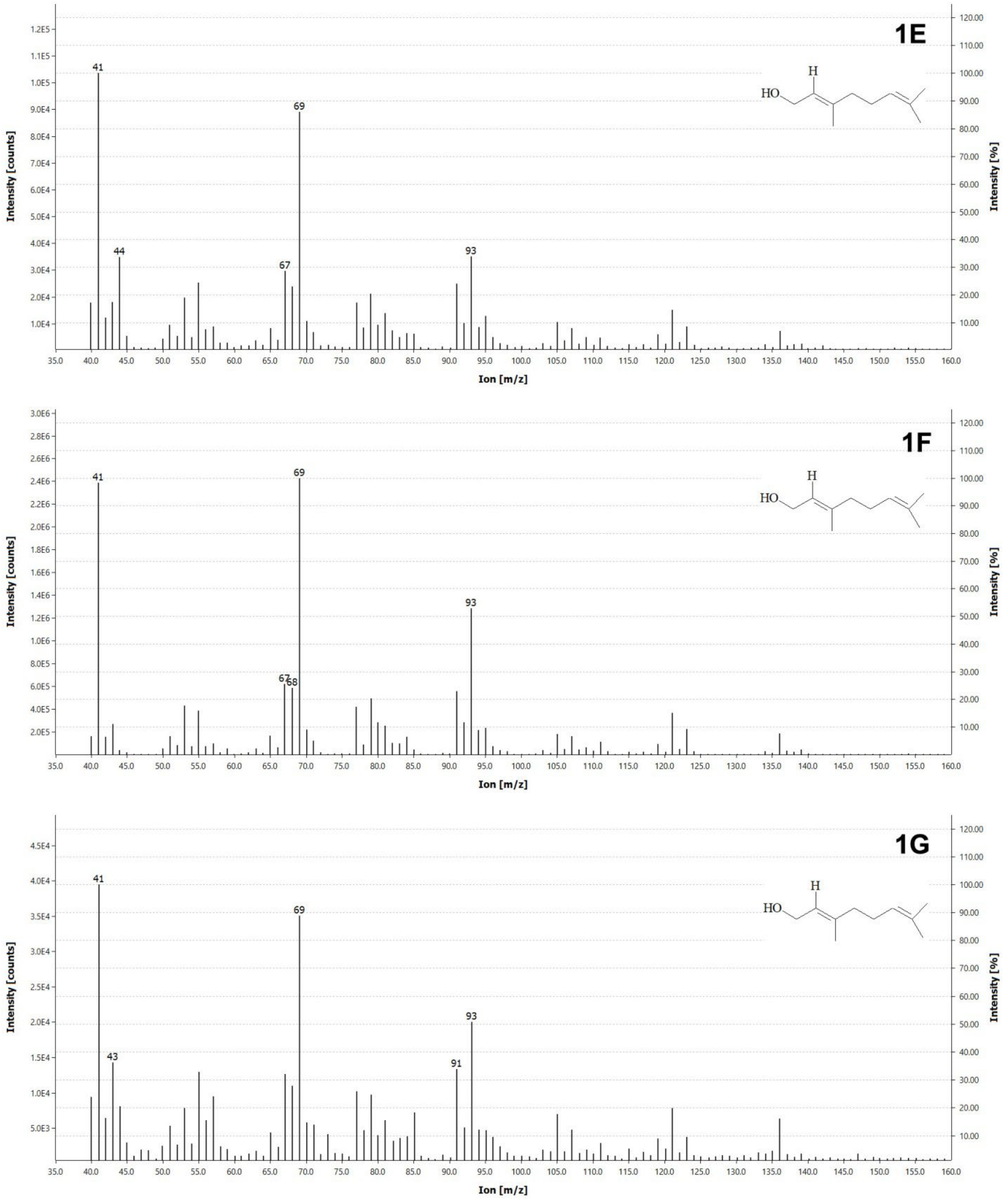

Figure S24: EI mass spectra of geraniol generated by dephosphorylation of products of *trans*IDS11 (1E), *trans*IDS2 (1F), and *trans*IDS17 (1G), related to Figure 3. For chromatograms, see Figure S23.

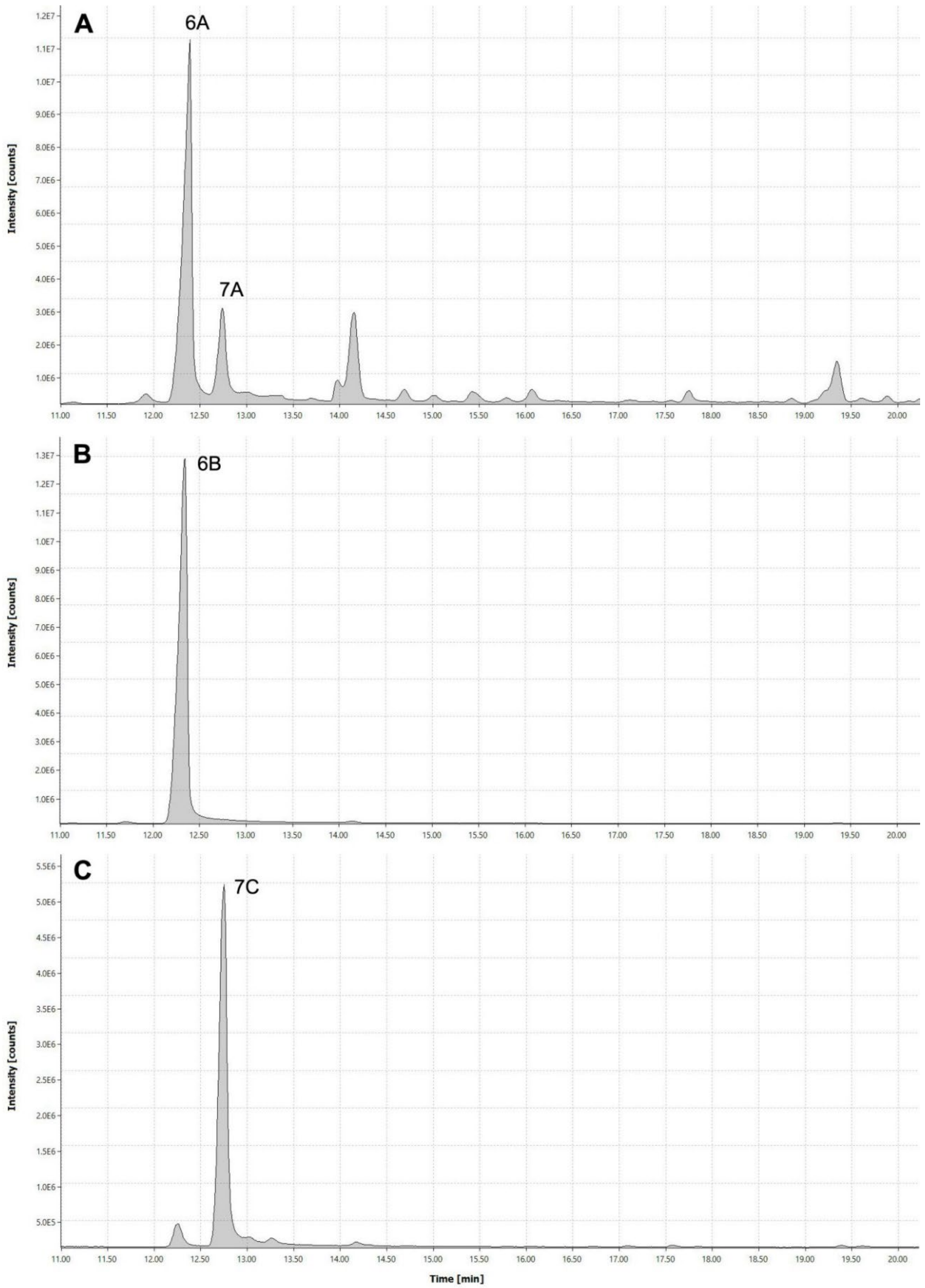

Figure S25: Identification of irregular monoterpenes synthesysed by *trans*IDS5, related to Figure 3. The protein was incubated with DMAPP only, and dephosphorylated products were analysed by GC-MS. A) Dephosphorylated products of *trans*IDS5 – lavandulol (peak 6A) and maconelliol (peak 7A); B) dephosphorylated product of lavandulyl diphosphate synthase from *Lavandula x intermedia* – lavandulol (peak 6B); C) chemically synthesized maconelliol (peak 7C). EI-MS data of the peaks are shown in Figures S26 and S27.

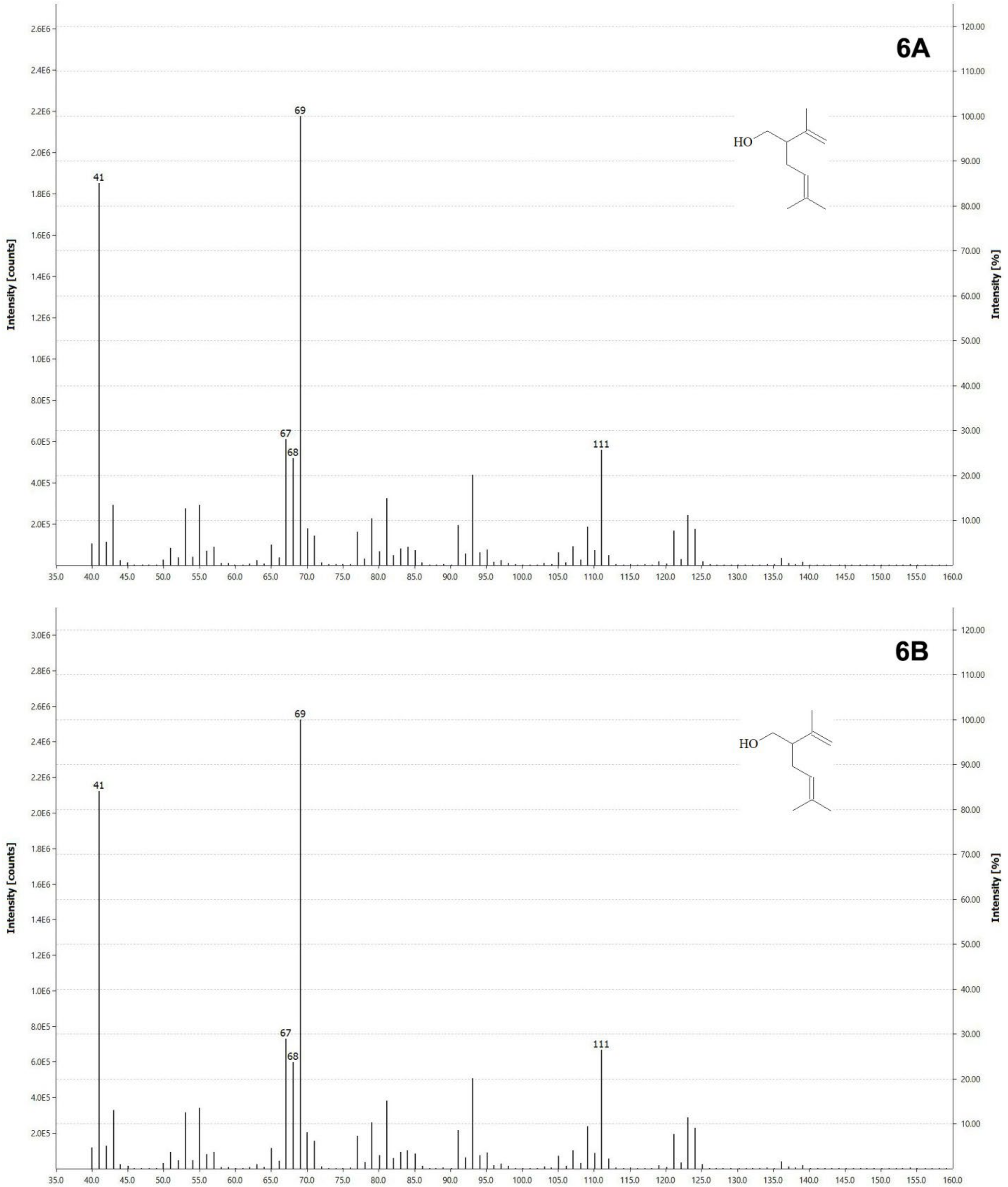

Figure S26: EI mass spectra of lavandulol generated by dephosphorylation of products of *trans*IDS5 (6A) and lavandulyl diphosphate synthase from *Lavandula* x *intermedia* (6B), related to Figure 3. For chromatograms, see Figure S25.

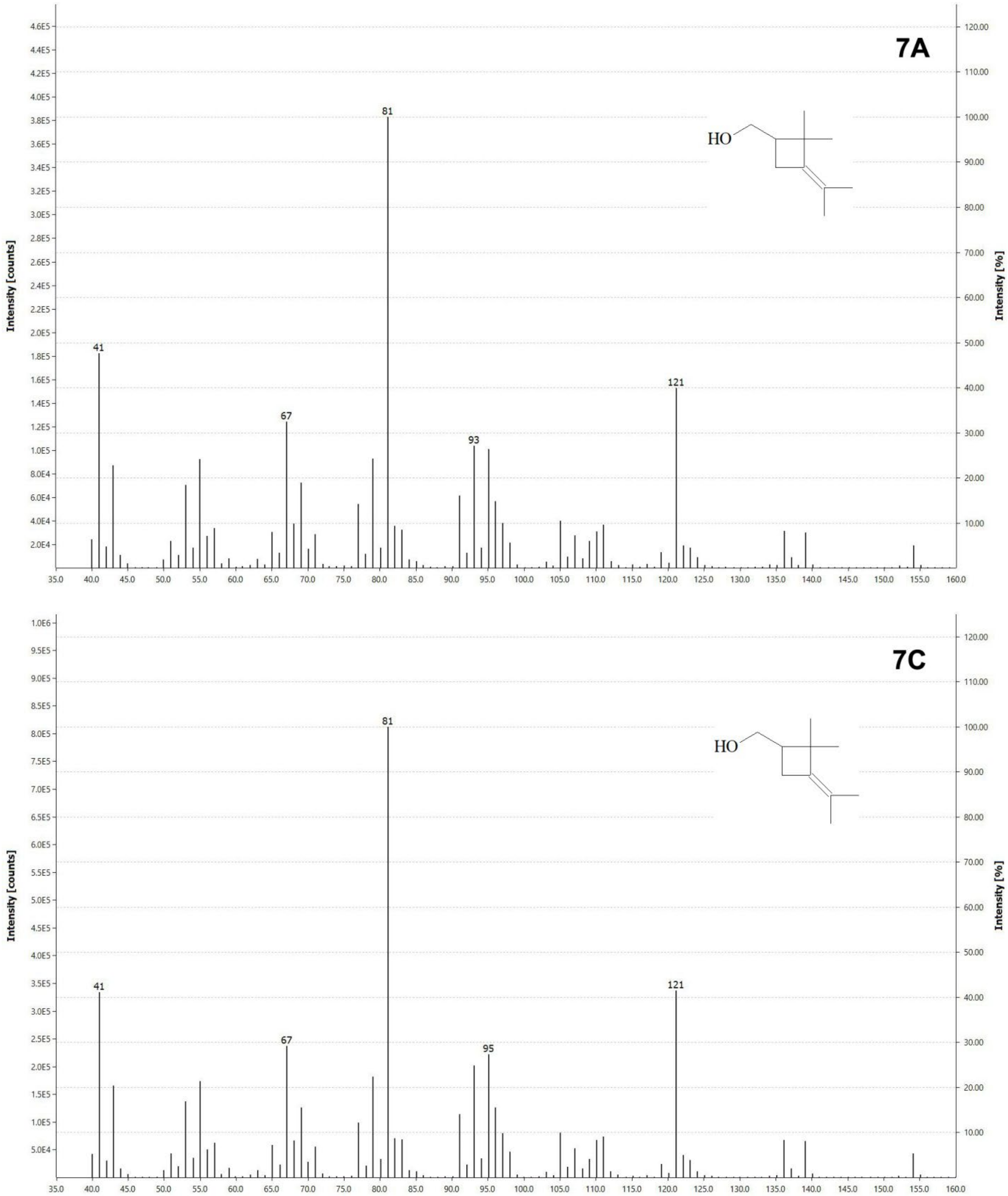

Figure S27: EI mass spectra of maconelliol generated by dephosphorylation of products of *trans*IDS5 (7A) and chemical synthesis (7C), related to Figure 3. For chromatograms, see Figure S25.
